# A Bio-Inspired Pursuit–Evasion Game and Fixed-Time Safety Control Method for USV Swarm Encirclement

**DOI:** 10.3390/biomimetics11100735

**Published:** 2026-10-14

**Authors:** Fengxiang Mao, Liang Xu, Jingjing Guo, Dongsong Zhang

**Affiliations:** School of Computer Science and Artificial Intelligence, Xinyang University, Xinyang 464000, China

**Keywords:** unmanned surface vehicle swarm, cooperative encirclement, dynamic game, fixed-time control, control barrier function

## Abstract

Unmanned Surface Vehicle (USV) swarms face significant challenges during dynamic target encirclement in complex marine environments, particularly regarding strong target maneuverability and strict safety constraints. To address these issues, this paper proposes a novel encirclement and capture method. The proposed approach integrates a bio-inspired pursuit-evasion dynamic game with fixed-time safety control. Drawing on the behavioral mechanism of dynamic cooperative blocking and escape space compression in group predation in nature, a multi-pursuer single-evader dynamic game model is established. The payoff functions of pursuers and the evader are constructed to characterize the strategic interaction between both sides, and adaptive cooperative encirclement strategies for the USV swarm are generated based on dynamic game equilibrium. To address environmental disturbances and the requirement of rapid response in complex marine environments, a fixed-time safety controller is designed. Taking the desired encirclement positions generated by the dynamic game as control objectives, a safety constraint mechanism is constructed by introducing control barrier functions, which achieves rapid convergence and cooperative collision avoidance of the USV swarm. Furthermore, the fixed-time convergence of position errors and the satisfaction of safety constraints for the control system are proven via Lyapunov stability theory. Finally, dynamic target encirclement numerical simulations under ocean current disturbances verify the effectiveness of the proposed method. The results demonstrate that the USV swarm can adapt to continuous target maneuvering, realize stable encirclement and capture, and satisfy safety constraints.

## 1. Introduction

With the rapid advancement of marine intelligent equipment technologies, Unmanned Surface Vehicles (USVs) exhibit extensive application prospects in marine environmental monitoring, target search, maritime patrol, and task execution in complex sea areas by virtue of their advantages, including autonomous navigation, remote control, and multi-platform cooperation [1,2,3]. Compared with single-platform operation modes, USV swarms rely on information interaction and cooperative decision-making among multiple agents to fully exploit the numerical superiority of platforms, thereby improving task execution efficiency and environmental adaptability [4]. Among relevant research directions, cooperative encirclement of dynamic targets integrates core technologies such as target tracking, path planning, formation control, and autonomous decision-making, which constitutes a vital research branch in the field of intelligent cooperation for USV swarms [5]. Nevertheless, numerous challenges remain to realize fast, stable, and safe encirclement of dynamic targets by multiple USVs under complex and dynamic marine environments [6].

Numerous group predation behaviors in nature, such as orca encirclement hunting and dolphin cooperative fish herding, exhibit superior characteristics of high distribution, self-organization, and collaborative decision-making. Multiple predators continuously compress the escape space of prey through role division, dynamic encirclement, and real-time strategy adjustment, thereby achieving efficient encirclement and capture with only local information interaction. This closed-loop process of “perception, decision-making, and encirclement” is highly consistent with the dynamic target encirclement and capture of USV swarms, providing a crucial biomimetic inspiration for USV cooperative encirclement operations [7]. Accordingly, USV encirclement and capture is essentially a biomimetic pursuit-evasion process, whose core problem can be abstracted as a multi-agent pursuit-evasion game.

On the one hand, pursuit USVs need to adjust their positions and velocities according to the target’s motion state and form an effective blockade zone via reasonable spatial distribution. On the other hand, the target vessel possesses autonomous maneuver capabilities and adopts escape strategies such as acceleration and steering based on the encirclement situation, rendering the entire encirclement process distinctly dynamically adversarial. In addition, wind, wave, and current disturbances, as well as uncertainties in USV dynamic models, exist in practical marine environments, which prevent the encirclement control system from acquiring accurate motion state information. Meanwhile, restricted by the bandwidth and transmission range of surface wireless communication, multiple USVs generally conduct cooperative decision-making merely via local information. Therefore, developing autonomous cooperation and dynamic encirclement control schemes for multiple USVs under limited communication conditions and complex environmental disturbances is an urgent research topic in the field of intelligent control for unmanned swarms [8].

Existing research on USV encirclement control mainly concentrates on containment control [9], cooperative formation control [10], and intelligent optimal control [11]. In terms of containment control, relevant studies design distributed control protocols to enable multiple unmanned platforms to gradually form containment structures around dynamic targets based on relative target positions. For instance, consensus theory-based containment control methods adjust platform motion states through information exchange between neighboring nodes [12], which deliver favorable stability in theoretical analysis. However, such methods generally rely on prior information of target velocity and position, and their encirclement performance is susceptible to complex target maneuvers and environmental disturbances. Moreover, some studies [13] introduce extended state observers and sliding mode control to estimate target motion states and compensate for unknown disturbances, enhancing system robustness, yet they suffer from complicated control parameter design and limited convergence speed.

For cooperative formation encirclement, leader-follower structures, virtual structures, and artificial potential field methods are widely applied to USV swarm control. The leader-follower method realizes formation maintenance by designating reference nodes, featuring a simple structure and convenient engineering implementation [14]; the virtual structure method predefines expected geometric relationships to sustain stable USV formations [15]. However, these approaches are constrained by fixed formation structures and struggle to adapt to variations in target motion states. When the target travels at high speed or abruptly changes its heading, fixed formation structures may reduce encirclement efficiency and even allow the target to break through the blockade. Additionally, although artificial potential field methods simultaneously account for target attraction and inter-platform collision avoidance constraints, they are prone to local minimum values, which pose risks of path planning failure in complex dynamic environments [16].

In addition to conventional containment and formation-based methods, recent studies have increasingly focused on reinforcement learning, adaptive consensus control, and robust nonlinear control for cooperative unmanned surface vehicle systems. Reinforcement learning-based methods enable multiple agents to learn cooperative encirclement and decision-making policies through interactions with dynamic environments, and have shown advantages in handling nonlinear system dynamics and uncertain target behaviors [6,10,11]. Adaptive consensus and distributed adaptive control methods can compensate for heterogeneous agent dynamics, communication variations, and uncertain model parameters, thereby improving cooperative formation performance under complex operating conditions [17,18]. Meanwhile, robust nonlinear control methods, including sliding-mode control, adaptive control, and disturbance-observer-based control, can improve tracking accuracy and disturbance rejection in the presence of model uncertainties and marine environmental disturbances [19,20].

Nevertheless, reinforcement learning-based approaches generally require considerable training data and their closed-loop stability and safety properties are difficult to guarantee explicitly. Adaptive consensus and robust nonlinear control methods mainly focus on the motion execution layer, whereas the strategic interaction between multiple pursuers and an actively evasive target is generally not explicitly incorporated into the control framework. Therefore, a unified encirclement framework that simultaneously considers adversarial decision-making, rapid convergence, disturbance rejection, and safety constraints is still required for dynamic target encirclement by USV swarms.

In recent years, an increasing number of studies have designed cooperative control strategies for unmanned systems by learning from group predation behaviors in nature. Predation behaviors including orca hunting, dolphin fish herding and wolf pack hunting embody the characteristics of distributed cooperation, self-organized formation and dynamic role assignment, offering novel research ideas for the cooperative control of unmanned swarms. Nevertheless, most existing studies on biomimetic encirclement focus primarily on the design of behavioral rules, lack rigorous analysis of optimal strategies, and can hardly guarantee system convergence and safety constraints. Therefore, integrating biomimetic cooperative mechanisms with dynamic game theory can further improve the adaptability and theoretical completeness of encirclement strategies. Obvious competitive relationships exist between targets and capture platforms during encirclement missions. Game theory can characterize the decision-making process among multiple agents from the perspective of strategic interaction, serving as an effective modeling tool for dynamic pursuit-evasion problems [21]. Some investigations adopt non-cooperative games to describe task conflicts among multiple agents and obtain cooperative strategies by solving the Nash equilibrium [22]. Other studies further introduce dynamic game theory, incorporating target motion and control inputs into the optimization framework to realize real-time strategy adjustment between pursuers and evaders [23]. Compared with traditional rule-based encirclement methods, dynamic games can fully account for variations in target behaviors and enhance the initiative and adaptability of encirclement strategies. However, most existing game-based methods concentrate on optimization at the decision-making layer, with insufficient consideration of fast convergence, safety constraints and disturbance rejection in the control execution process under complex marine environments. Consequently, the derived theoretical strategies cannot directly satisfy the practical motion control requirements of USVs.

On the other hand, fixed-time control theory has attracted widespread attention to enhance the rapid response capability of USV swarm control systems. Different from conventional asymptotically stable control methods, fixed-time control guarantees the convergence of system states within a predetermined time range independent of initial states, making it more suitable for unmanned systems with explicit task time constraints [19]. However, current fixed-time control methods primarily target single-platform trajectory tracking and formation maintenance, with limited research on multi-agent cooperative constraints, safe distance constraints, and adversarial target behaviors during dynamic target encirclement. Furthermore, USVs must simultaneously satisfy inter-platform collision avoidance, target tracking, and control input constraints during actual operation; stability design based solely on fixed-time theory cannot guarantee operational safety throughout the encirclement process.

Control Barrier Function (CBF) offers an effective solution to the aforementioned safety constraint issues [24]. By constructing constraint functions, CBF converts collision avoidance and regional limits into restrictions on control inputs, which can enhance control safety while ensuring system stability. Accordingly, integrating dynamic game decision mechanisms with fixed-time safety control methods can form a complete closed-loop control framework covering strategy generation to safe execution, boosting the dynamic target encirclement capability of USV swarms in complex environments.

Based on the above analysis, this paper proposes an encirclement method for USV swarms combining dynamic game and fixed-time safety control. First, a dynamic pursuit-evasion game model between the target vessel and multiple USVs is constructed, unifying target escape behaviors, cooperative constraints of pursuit vessels, and formation maintenance requirements into a dynamic optimization problem. Real-time encirclement strategies are obtained by solving game equilibria. Second, a fixed-time safety controller is designed to take the expected encirclement trajectories generated by dynamic games as control targets, enabling fast and stable tracking by USVs. Meanwhile, control barrier functions are introduced to construct a safety constraint mechanism, ensuring collision avoidance and consistent safe distance maintenance among multiple USVs during cooperation. Furthermore, a disturbance observation and compensation mechanism is developed to improve the robustness of the system under complex marine conditions, including wind, waves, and currents.

The main contributions of this paper are summarized as follows:

1. A bio-inspired dynamic game model is formulated for cooperative encirclement of a moving target by multiple USVs. The model incorporates pursuit and evasion strategies, cooperative encirclement objectives, and formation requirements into a pursuit–evasion payoff formulation. This formulation represents the strategic interaction between the pursuing vessels and the target and provides reference trajectories for encirclement control.
2. An encirclement controller combining fixed-time control and control barrier functions is developed. The fixed-time control component addresses tracking of the reference trajectories, while the control barrier functions impose inter-vessel safety-distance constraints. The convergence and safety properties are analyzed under the stated assumptions.
3. A disturbance-observer-based compensation mechanism is incorporated into the control framework to account for environmental disturbances and model uncertainties. The observer estimates the lumped disturbances online, and the resulting estimates are used for feedback compensation. Numerical simulations evaluate tracking accuracy, safety-constraint satisfaction, and control effort under ocean-current disturbances.

The remainder of this paper is organized as follows. Section 2 presents the USV dynamic model, preliminaries of graph theory, and formulation of the dynamic game problem. Section 3 designs the cooperative encirclement strategy based on the bionic dynamic game and analyzes the equilibrium solution process. Section 4 proposes the fixed-time safety controller and provides the stability proof. Section 5 verifies the effectiveness of the proposed method via simulation experiments. Section 6 concludes this paper and discusses future research directions.

## 2. Preliminaries and Problem Formulation

### 2.1. Graph Theory Preliminaries

Information exchange between USVs during swarm cooperative control can be described via graph theory [25]. Consider a multi-agent system composed of *N* USVs, whose communication topology is represented as a directed graph:

(1) $$\vec{G}=\left(V,E,A\right)$$

where V={1,2,⋯,N} denotes the set of USV nodes, E⊆V×V denotes the set of information interaction edges between USVs, and $A=\left[a_{ij}\right]\in R^{N\times N}$ denotes the adjacency matrix. If USV *i* can receive state information from USV *j*, then $a_{ij}>0$; otherwise, $a_{ij}=0$. This paper assumes that USVs do not exchange information with themselves, such that $a_{ii}=0$.

For the *i*-th USV, its neighbor node set is defined as:

(2) $$N_{i}=\left\{j\in V:a_{ij}>0\right\}$$

where $N_{i}$ represents the set of neighboring USVs from which the *i*-th USV can directly acquire state information. The degree matrix can be further defined based on the adjacency matrix:

(3) $$D=\mathrm{diag}(d_{1},d_{2},\ldots ,d_{N})$$

where

(4) $$d_{i}=\sum_{j=1}^{N}a_{ij}$$

denotes the in-degree of the *i*-th node. The Laplacian matrix corresponding to the communication topology is subsequently defined as [17]:

(5) L=D−A

where *L* characterizes information interaction within the USV swarm and serves as a fundamental basis for designing distributed cooperative control algorithms.

This paper adopts a local communication scheme among USVs, meaning each USV performs decision-making only using its own state, neighboring USV states, and target state information, without relying on centralized global control. To guarantee the completion of swarm cooperative tasks, the following assumption is made:

**Assumption** **1.** *The directed communication graph of USVs is connected, and at least one USV can directly obtain state information of the target vessel.*

This assumption ensures that target information can propagate throughout the entire USV swarm via the communication topology, enabling each USV to complete cooperative encirclement tasks using only local information.

### 2.2. Dynamic Game Theory

The USV swarm encirclement problem exhibits prominent pursuit-evasion adversarial characteristics. Pursuit USVs aim to reduce distances to the target and form stable containment configurations via coordinated motion, while the target vessel adjusts its motion state and control strategies to maximize escape probability. Accordingly, this problem can be formulated as a dynamic game process of multiple intelligent agents.

Consider a dynamic system consisting of *N* pursuit USVs and 1 evader target vessel, whose system state variable is expressed as:

(6) $$X={\left[x_{1},x_{2},\ldots ,x_{N},x_{T}\right]}^{T}$$

where $x_{i}$ denotes the state of the *i*-th pursuit USV and $x_{T}$ denotes the state of the target vessel.

The system dynamics are formulated as:

(7) $$\dot{X}=f\left(X,U\right)$$

where $U={\left[u_{1},u_{2},\ldots ,u_{N},u_{T}\right]}^{T}$ represents the control inputs of all participants, $u_{i}$ denotes the control input of the *i*-th pursuit USV, and $u_{T}$ denotes the control input of the target vessel.

For the *i*-th pursuit USV, the performance index function is defined as:

(8) $$J_{i}=\int_{0}^{T_{f}}L_{i}\left(X,U\right)dt$$

where

(9) $$L_{i}=q_{1}{∥p_{i}-p_{T}∥}^{2}+q_{2}{∥p_{i}-p_{i}^{d}∥}^{2}+q_{3}{∥u_{i}∥}^{2}$$

In the above expression, $p_{i}={\left[x_{i},y_{i}\right]}^{T}$ represents the position of the *i*-th USV, $p_{T}={\left[x_{T},y_{T}\right]}^{T}$ represents the target position, $p_{i}^{d}$ represents the desired encirclement position of the USV, and $q_{1},q_{2},q_{3}$ are positive weighting coefficients. The first term describes the requirement for the USV to approach the target, the second term ensures formation maintenance during encirclement, and the third term restricts energy consumption of control inputs.

The performance index of the evader target vessel is defined as:

(10) $$J_{T}=\int_{0}^{T_{f}}L_{T}\left(X,U\right)dt$$

where

(11) $$L_{T}=-r_{1}{∥p_{T}-p_{i}∥}^{2}+r_{2}{∥u_{T}∥}^{2}$$

In this formula, $r_{1}$ and $r_{2}$ denote weighting coefficients for the target’s payoff. Since the target vessel intends to expand distances from pursuit USVs, the first term takes a negative value to characterize its payoff from escape.

Thus, a dynamic game is formed between pursuit USVs and the target vessel:

(12) $$\min_{u_{i}}J_{i},\;i=1,2,\ldots ,N$$

(13) $$\max_{u_{T}}J_{T}$$

At the dynamic game equilibrium state, no participant can further optimize its own payoff by independently modifying its strategy, satisfying:

(14) $$J_{i}\left(u_{i}^{*},u_{-i}^{*}\right)\leq J_{i}\left(u_{i},u_{-i}^{*}\right)$$

where $u_{i}^{*}$ denotes the optimal control strategy of the *i*-th pursuit USV, and $u_{-i}^{*}$ denotes the set of optimal strategies of all participants except the *i*-th USV.

The Hamiltonian function is further defined as [26]:

(15) $$H=L\left(X,U\right)+\nabla V^{T}f\left(X,U\right)$$

where V(X) represents the system value function and ∇V denotes the gradient of the value function with respect to state variables.

The dynamic game equilibrium satisfies the Hamilton-Jacobi-Isaacs condition [27]:

(16) $$\frac{\partial H}{\partial u_{i}}=0,\;\frac{\partial H}{\partial u_{T}}=0$$

By solving the above equation, the optimal dynamic strategies of pursuit USVs and the evader target can be acquired.

### 2.3. Unmanned Surface Vehicle Dynamic Model

Practical USVs feature underactuation, strong nonlinearity, and environmental disturbances. This paper adopts a three-degree-of-freedom motion model to describe USV motion states [18]. The position and attitude of the *i*-th USV in the Earth-fixed coordinate frame are expressed as:

(17) $$\eta_{i}={\left[x_{i},y_{i},\psi_{i}\right]}^{T}$$

where $x_{i}$ and $y_{i}$ denote the longitudinal and transverse positions of the USV in the inertial frame, respectively, and $\psi_{i}$ denotes the USV yaw angle. The USV velocity state is defined as:

(18) $$\nu_{i}={\left[u_{i},v_{i},r_{i}\right]}^{T}$$

where $u_{i}$ denotes surge velocity, $v_{i}$ denotes sway velocity, and $r_{i}$ denotes yaw angular velocity in the body-fixed frame. The USV kinematic model is:

(19) $${\dot{\eta }}_{i}=R\left(\psi_{i}\right)\nu_{i}$$

where

(20) $$R\left(\psi_{i}\right)=\left[\begin{array}{ccc} \cos\psi_{i} & -\sin\psi_{i} & 0\\ \sin\psi_{i} & \cos\psi_{i} & 0\\ 0 & 0 & 1 \end{array}\right]$$

represents the rotation matrix transforming the body-fixed frame to the Earth-fixed frame.

**Figure 1:**
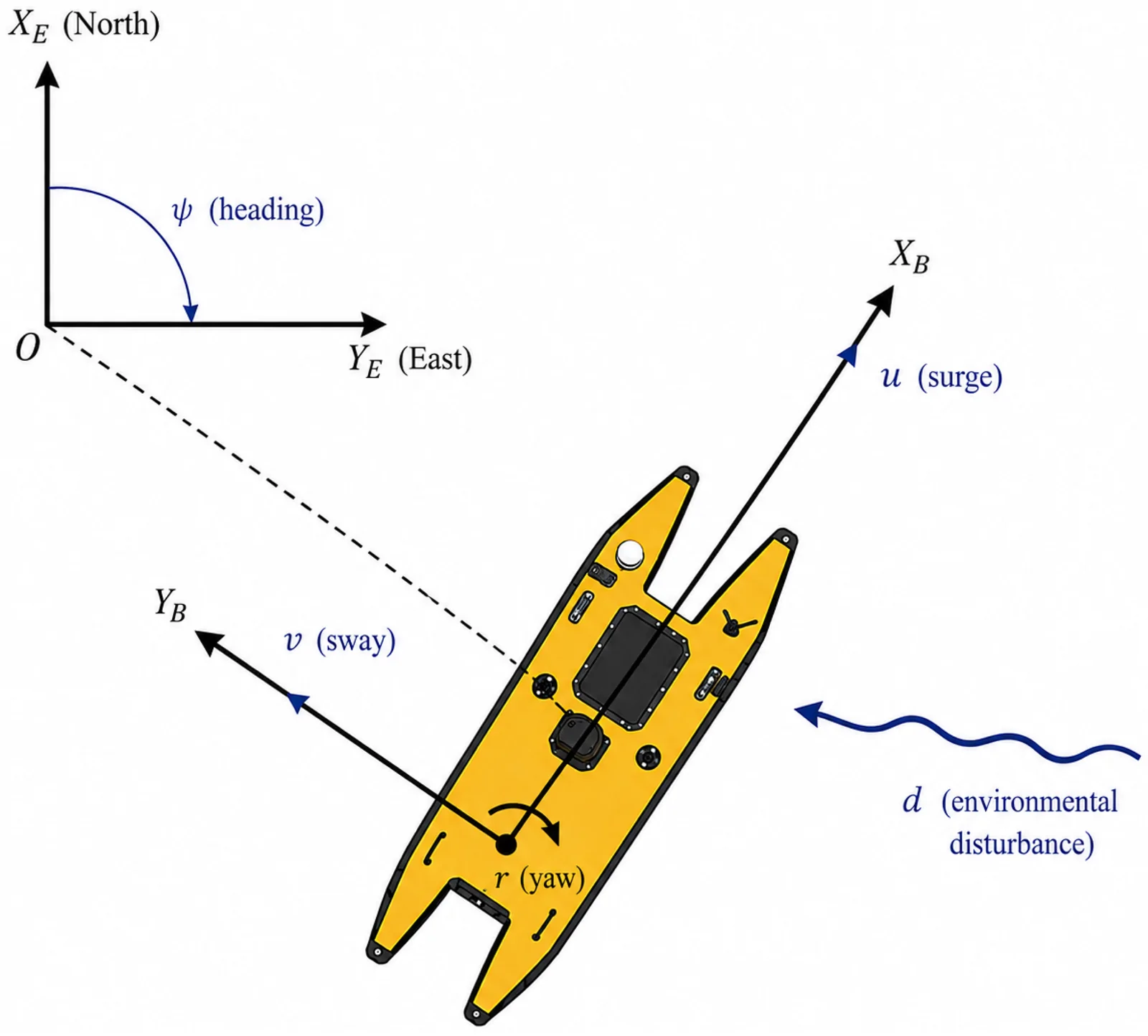
Schematic diagram of Unmanned Surface Vehicle (USV) reference coordinate system. The dashed line connects the origins of the earth-fixed and body-fixed coordinate systems, indicating the USV’s position in the earth-fixed frame.

As illustrated in Figure 1, the spatial motion of the USV is projected onto the 2D horizontal plane for the pursuit-evasion task. The USV dynamic model is formulated as [28]:

(21) $$M_{i}{\dot{\nu }}_{i}+C_{i}\left(\nu_{i}\right)\nu_{i}+D_{i}\left(\nu_{i}\right)\nu_{i}=\tau_{i}+d_{i}$$

where $M_{i}$ denotes the inertial matrix incorporating hull mass and added mass; $C_{i}\left(\nu_{i}\right)$ denotes the Coriolis and centrifugal force matrix; $D_{i}\left(\nu_{i}\right)$ denotes the hydrodynamic damping matrix; $\tau_{i}$ denotes the control input vector; and $d_{i}$ represents external environmental disturbances induced by wind, waves, and currents.

In practical physical terms, Equation (21) balances the forces acting on the USV. The left side of the equation represents the vessel’s inherent physical resistance to motion, including its inertia, rotational forces, and water drag. The right side represents the external forces dictating its movement, specifically the active thrust generated by its propellers ($\tau_{i}$) and the unpredictable pushes from ocean winds, waves, and currents ($d_{i}$).

The control input is defined as:

(22) $$\tau_{i}={\left[\tau_{ui},\tau_{ri}\right]}^{T}$$

where $\tau_{u,i}$ denotes the thrust input and $\tau_{r,i}$ denotes the steering control moment.

Assuming the hull is symmetric about the longitudinal axis with uniform mass distribution [20], the matrix *M* is diagonal, i.e., $M=\mathrm{diag}(m_{11},m_{22},m_{33})$. Equation (21) can be expanded and rewritten into a control-oriented dynamic model:

(23) $$\left\{\begin{array}{c} \dot{u}=\frac{m_{22}}{m_{11}}vr-\frac{d_{11}}{m_{11}}u+\frac{1}{m_{11}}\tau_{u}+\sigma_{u}\\ \dot{v}=-\frac{m_{11}}{m_{22}}ur-\frac{d_{22}}{m_{22}}v+\sigma_{v}\\ \dot{r}=\frac{m_{11}-m_{22}}{m_{33}}uv-\frac{d_{33}}{m_{33}}r+\frac{1}{m_{33}}\tau_{r}+\sigma_{r} \end{array}\right.$$

where $m_{ii}$ and $d_{ii}$ represent nominal hydrodynamic parameters; $\sigma_{u},\sigma_{v},\sigma_{r}$ denote lumped disturbance terms encompassing parametric uncertainties, high-order nonlinear terms, and external environmental interferences.

Accounting for model uncertainties and unknown disturbances, the USV dynamics are further rewritten as:

(24) $${\dot{\nu }}_{i}=f_{i}\left(\nu_{i}\right)+B_{i}\tau_{i}+d_{i}$$

where $f_{i}\left(\nu_{i}\right)$ denotes unknown nonlinear dynamics, $B_{i}$ denotes the control input matrix, and $d_{i}$ represents comprehensive disturbances.

### 2.4. Target Vessel Motion Model

The target vessel also adopts a three-degree-of-freedom motion model:

(25) $${\dot{\eta }}_{T}=R\left(\psi_{T}\right)\nu_{T}$$

where $\eta_{T}={\left[x_{T},y_{T},\psi_{T}\right]}^{T}$ denotes the position and heading state of the target vessel, and $\nu_{T}={\left[u_{T},v_{T},r_{T}\right]}^{T}$ denotes the target motion velocity. The target vessel control input is expressed as:

(26) $$u_{T}={\left[a_{T},\omega_{T}\right]}^{T}$$

where $a_{T}$ denotes the variation in target velocity and $\omega_{T}$ denotes the target yaw angular velocity.

Given the randomness and unpredictability of target motion, the target motion disturbance is assumed to satisfy:

(27) $$∥d_{T}∥\leq d_{Tmax}$$

where $d_{Tmax}$ denotes the upper bound of target disturbances.

### 2.5. Formulation of Encirclement Control Problem

This paper investigates the problem of cooperative encirclement of a single dynamic target by *N* USVs. To form an effective encirclement configuration, USVs are expected to be uniformly distributed around the target.

**Figure 2:**
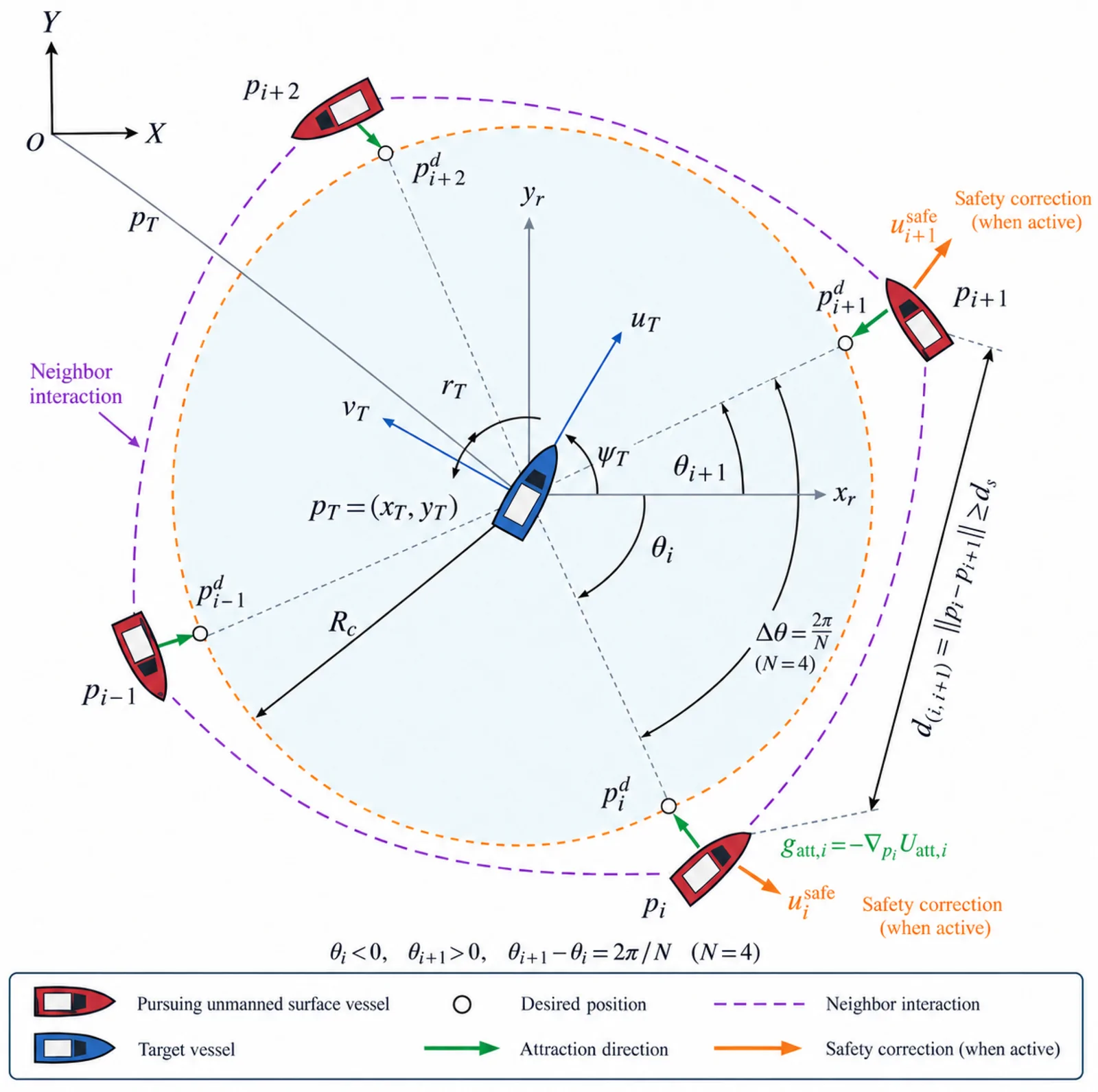
Swarm encirclement geometry and control interactions. Red and blue vessels denote pursuers and the target, respectively; open circles indicate desired positions. Green arrows show attraction directions, orange arrows indicate safety corrections, and purple dashed links represent neighbor interactions. The black dashed lines indicate the radial reference directions from the target to the desired encirclement positions. The orange dashed circle represents the desired encirclement circle with radius $R_{c}$.

As depicted in Figure 2, the encirclement objective requires the USV swarm to dynamically form a closed spatial constraint around the evasive target rather than maintaining a static geometric shape.The desired encirclement position of the *i*-th USV is defined as:

(28) $$p_{i}^{d}=p_{T}+R_{c}\left[\begin{array}{c} \cos\theta_{i}\\ \sin\theta_{i} \end{array}\right]$$

where $R_{c}$ denotes the desired encirclement radius and $\theta_{i}$ denotes the expected angle of the *i*-th USV in the encirclement formation.

The ideal encirclement state satisfies:

(29) $$∥p_{i}-p_{T}∥=R_{c}$$

and

(30) $$\theta_{i+1}-\theta_{i}=\frac{2\pi }{N}$$

meaning all USVs are uniformly distributed around the target. The encirclement position error is defined as:

(31) $$e_{i}=p_{i}-p_{i}^{d}$$

The control objective $\tau_{i}$ of this paper is to design a control law such that:

(32) $$\lim_{t\to T_{f}}e_{i}\left(t\right)=0$$

where $T_{f}$ denotes the fixed convergence time.

Meanwhile, collision avoidance among USVs must satisfy the safety constraint:

(33) $$∥p_{i}-p_{j}∥\geq d_{s}$$

where $d_{s}$ represents the minimum allowable safe distance between USVs.

Therefore, the problem addressed in this paper is formulated as follows: under conditions of dynamic target escape, marine environmental disturbances, and limited communication, design a dynamic game-based encirclement decision-making method combined with fixed-time safety control strategies to realize fast, stable, and safe encirclement of dynamic targets by USV swarms.

### 2.6. Basic Assumptions

To facilitate subsequent controller design, the following assumptions are proposed:

**Assumption** **2.** *Unknown nonlinear terms in USV dynamics are continuous and bounded, i.e., there exists a positive constant $f_{max}$ satisfying:*

(34) $$∥f_{i}\left(\nu_{i}\right)∥\leq f_{max}$$

**Assumption** **3.** *Marine environmental disturbances are bounded, i.e., there exists a positive constant $d_{max}$ satisfying:*

(35) $$∥d_{i}∥\leq d_{max}$$

**Assumption** **4.** *Pursuit USVs possess a speed advantage over the target vessel, i.e.:*

(36) $$v_{i}^{max}>v_{T}^{max}$$

*where $v_{i}^{max}$ and $v_{T}^{max}$ denote the maximum sailing speeds of pursuit USVs and the target vessel, respectively.*

**Remark** **1.** *We acknowledge that Assumption 4 imposes a strong condition that primarily serves to guarantee the strict mathematical proof of fixed-time trajectory tracking convergence. In practical marine scenarios where the target is equally agile or even faster ($v_{T}^{max}\geq v_{i}^{max}$), a single USV can hardly catch the target through pure speed pursuit. Under such circumstances, the proposed method relies on the numerical superiority and spatial distribution of the swarm rather than speed. By leveraging the dynamic game strategy, multiple USVs can predict the target’s escape tendency and cooperatively maneuver to optimal blocking positions ahead of time, progressively compressing the target’s available escape space. Exploring the theoretical capture conditions and topological encirclement guarantees for a faster evader will be a key focus of our future work.*

**Assumption** **5.** *The USV communication topology remains connected, and neighboring USVs can exchange necessary state information.*

## 3. Cooperative Encirclement Strategy Based on Bionic Dynamic Game

During the encirclement of dynamic targets by USV swarms, the target vessel actively adjusts its motion state according to the encirclement situation, rendering the entire encirclement process distinctly dynamically adversarial. Traditional methods relying on fixed formations or artificial rules only consider USV motion constraints and fail to describe the mutual influence between target escape behaviors and encirclement strategies. To resolve the above issue, this paper models the USV swarm encirclement process as a dynamic pursuit-evasion game problem, constructs strategic interaction relationships between pursuit USVs and the target vessel, solves the equilibrium state of the dynamic game, and generates real-time reference trajectories for USV encirclement.

In the proposed method, each pursuit USV acts as an independent decision-making agent, acquiring neighbor state information via local communication and adjusting motion strategies according to target states and self-task demands. Meanwhile, spatial distribution constraints among USVs are considered, and target tracking, formation maintenance, and collision avoidance requirements are integrated into the dynamic game performance index to achieve collaborative optimization from the task decision layer to the motion planning layer.

### 3.1. Construction of Bionic Pursuit-Evasion Dynamic Game Model

Inspired by the behavioral mechanism of “dynamic encirclement, cooperative space compression and escape direction blocking” during group prey herding by dolphins, this paper abstracts the USV encirclement process into a bionic pursuit-evasion game. The pursuer USVs correspond to predators, while the target vessel acts as the prey. Dynamic payoff functions are formulated to characterize the strategy evolution relationship between the two sides.

The biomimetic mechanism considered in this study does not directly imitate the motion trajectories of biological predators. Instead, it extracts three functional principles from natural group predation: dynamic blocking, cooperative spatial redistribution, and progressive compression of the prey’s escape space. Compared with traditional rule-based encirclement strategies, these mechanisms allow pursuers to continuously adjust their relative positions and cooperative roles according to the instantaneous escape behavior of the target, rather than maintaining a fixed geometric formation or following predetermined pursuit rules. This property is particularly important in marine pursuit–evasion scenarios, where the target may actively change its speed and heading and environmental disturbances may continuously alter the relative motion states of the participants.

As shown in Table 1, the key advantage of the biomimetic predation mechanism lies not in directly reproducing biological trajectories, but in extracting functional principles such as dynamic blocking, adaptive spatial redistribution, and progressive escape-space compression. Accordingly, these principles are incorporated into the dynamic game framework through pursuit-evasion payoff functions and adaptive encirclement-position generation, so that the target escape strategy and the cooperative blocking strategies of multiple pursuers are modeled as explicit strategic interactions rather than heuristic rules.

Consider a pursuit-evasion system consisting of *N* pursuit USVs and one dynamic target vessel. Based on the dynamic models established in Section 2, the overall system state is defined as:

(37) $$X={\left[p_{1},p_{2},\ldots ,p_{N},p_{T}\right]}^{T}$$

where $p_{i}={\left[x_{i},y_{i}\right]}^{T}$ denotes the position state of the *i*-th USV and $p_{T}={\left[x_{T},y_{T}\right]}^{T}$ denotes the target position state.

During encirclement tasks, USVs aim to approach the target as closely as possible while ensuring self-safety and forming a uniform containment configuration around the target. Accordingly, the objective function of the *i*-th USV is defined as:

(38) $$J_{i}=\int_{0}^{T_{f}}\left(Q_{i}+R_{i}\right)dt$$

where $Q_{i}$ denotes the task error cost and $R_{i}$ denotes the control energy consumption cost.

Further define:

(39) $$Q_{i}=\alpha_{1}{∥p_{i}-p_{T}∥}^{2}+\alpha_{2}{∥p_{i}-p_{i}^{d}∥}^{2}+\alpha_{3}{∥p_{i}-p_{j}∥}^{-2}$$

where the first term represents the distance constraint between the USV and the target vessel, which drives the pursuer USVs to approach the target and mimics the rapid advance of predators toward prey. The second term denotes the tracking error of the USV with respect to the desired encirclement position, which maintains the expected geometric configuration surrounding the target and simulates the uniform encirclement structure maintained by predator groups. The third term is the repulsive potential function among USVs to avoid excessive proximity between swarm members. Here, $p_{j}$ denotes the position state of the neighbor node of the *i*-th USV, and $\alpha_{1},\alpha_{2},\alpha_{3}$ are the corresponding weight coefficients.

Intuitively, this cost function simulates the natural instincts of a predator group. The first term acts as an attractive force driving the pursuer toward the prey. The second term acts as a structural constraint to maintain a coordinated hunting formation. The third term serves as a repulsive force, preventing the USVs from colliding with one another, much like predators maintaining safe spacing during a high-speed chase.

Building upon the standard pursuit-evasion game formulation introduced in Equation (9), the objective function for the proposed bionic encirclement task is augmented to account for inter-vessel safety. Specifically, the task error cost $Q_{i}$ defined in Equation (39) introduces an additional repulsive potential function term ($\alpha_{3}{∥p_{i}-p_{j}∥}^{-2}$) to ensure collision avoidance and maintain proper spatial distribution among swarm members, mirroring the cooperative mechanisms of biological predators.

The control energy consumption term is defined as:

(40) $$R_{i}=\beta_{i}{∥u_{i}∥}^{2}$$

where $\beta_{i}$ denotes the control weighting coefficient and $u_{i}$ denotes the control input of the *i*-th USV.

For the target vessel, its objective is to maximize distances from pursuit USVs while limiting its own control energy consumption under motion constraints. Therefore, the target payoff function is defined as:

(41) $$J_{T}=\int_{0}^{T_{f}}\left(\gamma_{1}{∥p_{T}-p_{i}∥}^{2}-\gamma_{2}{∥u_{T}∥}^{2}\right)dt$$

where $\gamma_{1},\gamma_{2}>0$ denote the weighting coefficients for the target’s escape payoff, $p_{T}={\left[x_{T},y_{T}\right]}^{T}$ denotes the target position, $p_{i}={\left[x_{i},y_{i}\right]}^{T}$ denotes the position of the *i*-th pursuit USV, and $u_{T}$ denotes the target control input. The first term indicates that the target gains a higher payoff by expanding distances from pursuit USVs, while the second term restricts excessive control inputs inconsistent with practical motion constraints.

Thus, a dynamic game is formed between the USV swarm and the target vessel:

(42) $$\min_{u_{i}}J_{i},\;i=1,\ldots ,N$$

(43) $$\max_{u_{T}}J_{T}$$

Equation (42) describes that pursuit USVs minimize task costs by optimizing their own control strategies, while Equation (43) describes that the target vessel improves escape payoff by adjusting its motion strategy.

### 3.2. Solution of Dynamic Game Equilibrium Strategies

Due to strategic coupling between USVs and the target vessel, optimizing the strategy of a single participant independently cannot yield globally optimal results. Accordingly, dynamic game theory is adopted to solve the equilibrium strategies of all participants.

The system value function is defined as:

(44) $$V\left(X\right)=\min_{u_{1},\ldots ,u_{N}}\max_{u_{T}}J\left(X,U\right)$$

The Hamiltonian function is constructed as:

(45) $$H=L\left(X,U\right)+\frac{\partial V}{\partial X}f\left(X,U\right)$$

where L(X,U) denotes the instantaneous system cost function and f(X,U) denotes the system state transition function.

The dynamic game equilibrium satisfies:

(46) $$\frac{\partial H}{\partial u_{i}}=0$$

(47) $$\frac{\partial H}{\partial u_{T}}=0$$

Based on Equations (46) and (47), the optimal control strategies of pursuit USVs and the target vessel can be derived:

(48) $$u_{i}^{*}=-\frac{1}{2}R_{i}^{-1}B_{i}^{T}\frac{\partial V}{\partial p_{i}}$$

(49) $$u_{T}^{*}=\frac{1}{2}R_{T}^{-1}B_{T}^{T}\frac{\partial V}{\partial p_{T}}$$

where $R_{i}$ and $R_{T}$ denote the control weighting matrices of pursuit USVs and the target vessel, respectively, and $B_{i}$, $B_{T}$ denote the control input matrices.

The distinction between the proposed dynamic game formulation and a basic artificial potential field controller lies in how the control strategies are determined. A basic artificial potential field controller generates control inputs directly from the spatial gradient of an instantaneous potential function. In the proposed formulation, the target-approaching, formation-tracking, and inter-vessel repulsion terms in Equation (39) are incorporated into the cumulative pursuit objective in Equation (38), while Equation (41) describes the target’s evasion objective. Equations (44)–(49) incorporate the pursuit and evasion actions together with their effects on the system dynamics. Consequently, the strategies in Equations (48) and (49) depend on value-function gradients that characterize cumulative performance under the pursuit–evasion interaction, rather than directly on the spatial gradient of the instantaneous cost. This formulation connects the encirclement objectives with the dynamic interaction between the pursuers and the target, and is integrated with the subsequent fixed-time tracking controller and safety constraints.

Since complete global state information cannot be acquired in practical USV systems, a distributed dynamic game framework is adopted, where strategy updates rely solely on neighbor information.

The local state of the *i*-th USV is defined as:

(50) $$X_{i}=\left[p_{i},p_{T},p_{j}\right]$$

where $p_{j}$ denotes the state of neighboring nodes of the *i*-th USV.

A distributed strategy update law is designed as:

(51) $$u_{i}\left(k+1\right)=u_{i}\left(k\right)-\lambda_{i}\nabla J_{i}$$

where $\lambda_{i}$ denotes the strategy update step size, and $\nabla J_{i}$ denotes the gradient of the cumulative performance index with respect to the control strategy of the *i*-th vessel. With the other participants’ current strategies held fixed during this update, Equation (51) represents a descent step for that vessel’s objective. Evaluating this gradient requires accounting for how its control strategy affects subsequent states and accumulated costs through the system dynamics, given the other pursuers’ and the target’s strategies. It should therefore be distinguished from the spatial gradient of the instantaneous attraction–repulsion cost in Equation (39).

Through iterative updates, the system state gradually converges to the dynamic game equilibrium:

(52) $$\lim_{k\to \infty }u_{i}\left(k\right)=u_{i}^{*}$$

Thereby obtaining optimal encirclement strategies satisfying target motion constraints and swarm coordination requirements.

### 3.3. Dynamic Encirclement Formation Generation Based on Game Equilibrium

Traditional encirclement methods adopt fixed circular formations:

(53) $$p_{i}^{d}=p_{T}+R_{c}{\left[\cos\theta_{i},\sin\theta_{i}\right]}^{T}$$

Nevertheless, when the target moves at high speed or changes its heading, a fixed encirclement structure can hardly block the escape directions of the target effectively. In nature, predators do not maintain a regular circular formation all the time; instead, they continuously adjust their blocking positions according to the prey’s escape direction. Accordingly, this paper adopts the outputs of the dynamic game to tune the encirclement angle of each USV in real time so as to realize a biomimetic adaptive encirclement configuration.

The target motion direction is defined as:

(54) $$\theta_{T}=\mathrm{atan}2\left({\dot{y}}_{T},{\dot{x}}_{T}\right)$$

A dynamic adjustment angle is introduced based on the target velocity direction:

(55) $$\theta_{i}^{d}=\theta_{i}^{0}+k_{\theta }\theta_{T}$$

where $\theta_{i}^{0}$ denotes the initial distribution angle of the USV and $k_{\theta }$ denotes the direction adjustment coefficient.

Accordingly, the desired position of the *i*-th USV is redefined as:

(56) $$p_{i}^{d}=p_{T}+R_{c}\left[\begin{array}{c} \cos\theta_{i}^{d}\\ \sin\theta_{i}^{d} \end{array}\right]$$

Equation (56) enables the encirclement formation to dynamically adapt to variations in target motion states.

It is important to note that Equations (54)–(56) constitute the explicit mathematical basis for the proposed cooperative mechanism. Rather than assigning heterogeneous roles, the collaboration among the USVs is mathematically defined as adaptive spatial redistribution. By coupling the desired formation angles ($\theta_{i}^{d}$) directly with the target’s instantaneous heading ($\theta_{T}$), the pursuers dynamically shift their geometric distribution to collaboratively block the target’s escape directions.

### 3.4. Distributed Encirclement Path Generation

To ensure USVs smoothly reach their desired encirclement positions, the path tracking error is defined as:

(57) $$e_{i}^{p}=p_{i}-p_{i}^{d}$$

The desired velocity is designed as:

(58) $$v_{i}^{d}=-k_{p}e_{i}^{p}+{\dot{p}}_{i}^{d}$$

where $k_{p}$ denotes the path tracking gain.

The desired heading angle is further calculated from the direction of the desired velocity:

(59) $$\psi_{i}^{d}=\mathrm{atan}2\left(v_{iy}^{d},v_{ix}^{d}\right)$$

The magnitude of the desired velocity is:

(60) $$u_{i}^{d}=\sqrt{{\left(v_{ix}^{d}\right)}^{2}+{\left(v_{iy}^{d}\right)}^{2}}$$

Equations (58)–(60) generate reference motion trajectories for USVs, which serve as input signals for the subsequent fixed-time safety controller.

### 3.5. Stability Analysis of Dynamic Game Encirclement

**Theorem** **1.** *If the performance functions of the dynamic pursuit-evasion game satisfy continuous differentiability, the performance function of each pursuit USV is strictly convex with respect to its own control variable, and the payoff function of the target vessel is strictly concave with respect to its own control variable, then the dynamic pursuit-evasion game admits at least one equilibrium strategy.*

**Proof.**  According to Equations (38)–(40), the performance function of each pursuit USV consists of state error terms, formation constraint terms, collision avoidance potential functions, and control energy consumption terms. The first three terms depend solely on system states, while the control-input-related term takes the form $R_{i}=\beta_{i}{∥u_{i}∥}^{2}$. The second derivative of the pursuit USV performance function with respect to its own control variable is:

(61) $$\frac{\partial^{2}J_{i}}{\partial u_{i}^{2}}=2\beta_{i}I>0$$

where *I* denotes the identity matrix. Since $\beta_{i}>0$, the performance function is strictly convex with respect to the self-control variable. Meanwhile, the state error term, formation maintenance term, and collision avoidance potential function are all continuous, rendering $J_{i}$ continuously differentiable with respect to $u_{i}$.

The target vessel payoff function in Equation (41) satisfies:

(62) $$\frac{\partial^{2}J_{T}}{\partial u_{T}^{2}}=-2\gamma_{2}I<0$$

Therefore, the target payoff function satisfies the strict concavity condition with respect to its own control variable $u_{T}$, meaning the target possesses a unique maximization strategy.

Furthermore, based on the existence theory of dynamic non-cooperative game equilibria, at least one Nash equilibrium strategy exists when the strategy space of each participant is a non-empty compact convex set, the performance function is continuous with respect to the self-strategy variable, and convexity or concavity conditions are satisfied.

Thus, equilibrium control strategies exist between pursuit USVs and the target vessel:

(63) $$U^{*}=\left[u_{1}^{*},\ldots ,u_{N}^{*},u_{T}^{*}\right]$$

where $u_{i}^{*}$ denotes the optimal control strategy of the *i*-th pursuit USV and $u_{T}^{*}$ denotes the optimal escape strategy of the target vessel.

This completes the proof. □

## 4. Design of Cooperative Encirclement Controller Based on Fixed-Time Safety Control

The dynamic game approach proposed in Section 3 constructs a pursuit-evasion decision model between the USV swarm and dynamic targets and derives desired encirclement trajectories satisfying task requirements. Nevertheless, USV dynamic systems exhibit nonlinearity, underactuation, and model uncertainty, while wind, wave, and current disturbances exist in marine environments. Consequently, the desired trajectories generated by dynamic games cannot be directly adopted as control inputs, necessitating the design of high-performance motion controllers to enable fast and accurate tracking of desired encirclement positions by USVs.

Conventional asymptotically stable control methods only guarantee ultimate state convergence without providing explicit convergence time bounds. In dynamic target encirclement tasks, task completion time directly determines encirclement success rate, highlighting the demand for control schemes with fast convergence capabilities. Additionally, collision avoidance among multiple USVs during cooperative motion cannot be guaranteed by trajectory tracking error convergence alone.

To address the above limitations, this paper proposes a safe encirclement control method integrating fixed-time control, disturbance observation compensation, and control barrier functions. Fixed-time control theory ensures USVs track game-generated desired encirclement trajectories within a preset time window; a disturbance observer estimates and compensates for unknown marine disturbances online; control barrier functions impose safety constraints to prevent inter-USV collisions.

### 4.1. Control Objectives

The bionic dynamic game generates encirclement strategies, and the fixed-time control realizes rapid strategy execution. Thus, a two-layer control framework of “biomimetic decision-making and safe execution” is established. The desired encirclement position of the *i*-th USV derived from dynamic game equilibrium strategies is defined as:

(64) $$p_{i}^{d}={\left[x_{i}^{d},y_{i}^{d}\right]}^{T}$$

where $p_{i}^{d}$ denotes the desired position generated by dynamic game equilibrium strategies.

Given the underactuated nature of USVs that cannot directly control lateral motion, a cascaded control structure consisting of an outer position loop and an inner velocity loop is adopted. First, the desired heading angle required for the USV to realize the expected motion direction is calculated from the desired encirclement trajectory:

(65) $$\psi_{i}^{d}=\mathrm{atan}2\left({\dot{y}}_{i}^{d},{\dot{x}}_{i}^{d}\right)$$

where $\psi_{i}^{d}$ denotes the desired heading angle required for the USV to follow the expected moving direction.

The position tracking error of the USV is defined as:

(66) $$e_{i}=p_{i}-p_{i}^{d}$$

where $e_{i}={\left[e_{xi},e_{yi}\right]}^{T}$ denotes the deviation between the actual position and the desired encirclement position.

The heading error is defined as:

(67) $$e_{\psi_{i}}=\psi_{i}-\psi_{i}^{d}$$

Considering the periodicity of heading angles, the heading error is normalized to restrict its range:

(68) $$e_{\psi_{i}}=\mathrm{wrap}\left(\psi_{i}-\psi_{i}^{d}\right)$$

where wrap(·) denotes the angle normalization function that limits the heading error within [−π,π].

Accordingly, the control objectives of this section are formulated as:

(69) $$\lim_{t\to T_{f}}e_{i}\left(t\right)=0$$

subject to

(70) $$\lim_{t\to T_{f}}e_{\psi_{i}}\left(t\right)=0$$

i.e., position and heading errors of USVs converge synchronously within a fixed time $T_{f}$ to realize stable encirclement of dynamic targets while satisfying safety distance constraints.

### 4.2. Design of Fixed-Time Position–Heading Controller

Fixed-time stability theory is adopted to design the position controller and improve the response speed of USV encirclement. Although conventional asymptotically stable control guarantees ultimate convergence, its convergence time depends on initial errors, making it unsuitable for encirclement tasks involving rapid target maneuvers.

A fixed-time nonlinear error function is defined as:

(71) $$\Phi \left(e_{i}\right)=c_{1}{\left|e_{i}\right|}^{\alpha }\mathrm{sign}\left(e_{i}\right)+c_{2}{\left|e_{i}\right|}^{\beta }\mathrm{sign}\left(e_{i}\right)$$

where $c_{1},c_{2}>0$ denote the control gains; α,β are fixed-time control exponents satisfying 0<α<1,β>1. The term with exponent α accelerates convergence under small errors, while the term with exponent β accelerates error decay under large initial deviations. sign(x) denotes the sign function:

(72) $$\mathrm{sign}\left(x\right)=\left\{\begin{array}{cc} 1, & \mathrm{if}\;x>0\\ 0, & \mathrm{if}\;x=0\\ -1, & \mathrm{if}\;x<0 \end{array}\right.$$

The desired velocity vector is designed as:

(73) $$v_{i}^{d}={\dot{p}}_{i}^{d}-\Phi \left(e_{i}\right)$$

where $v_{i}^{d}$ denotes the desired velocity vector of the USV.

Equation (73) yields:

(74) $${\dot{e}}_{i}={\dot{p}}_{i}-v_{i}^{d}=-\Phi \left(e_{i}\right)$$

Thus, the position error satisfies the fixed-time stability condition under ideal conditions.

Given the dynamic constraints on practical USV motion, the desired velocity needs to be further decomposed into desired surge speed and desired heading angle:

(75) $$\left\{\begin{array}{c} u_{i}^{d}=\sqrt{{\left({\dot{x}}_{i}^{d}\right)}^{2}+{\left({\dot{y}}_{i}^{d}\right)}^{2}}\\ \psi_{i}^{d}=\mathrm{atan}2\left({\dot{y}}_{i}^{d},{\dot{x}}_{i}^{d}\right) \end{array}\right.$$

where $u_{i}^{d}$ is the desired surge velocity of the USV, and $\psi_{i}^{d}$ denotes the desired heading angle of the USV.

Equation (75) converts the desired encirclement trajectories output from the dynamic game layer into desired velocity and heading commands for USV motion control. Consistent with Assumption 2 in Section 2, sway velocity $v_{i}$ of USVs is dissipative and bounded; thus, high-order influences of lateral velocity on position control are neglected, and desired trajectory tracking is realized via adjustments to longitudinal speed and heading angle.

To achieve fixed-time convergence of heading errors, a fixed-time heading error function is defined:

(76) $$\Phi_{\psi }\left(e_{\psi i}\right)=c_{3}{\left|e_{\psi i}\right|}^{\alpha_{\psi }}\mathrm{sign}\left(e_{\psi i}\right)+c_{4}{\left|e_{\psi i}\right|}^{\beta_{\psi }}\mathrm{sign}\left(e_{\psi i}\right)$$

where $c_{3},c_{4}>0$ denote the heading control gains and $\alpha_{\psi },\beta_{\psi }$ denote fixed-time control exponents for heading satisfying $0<\alpha_{\psi }<1,\beta_{\psi }>1$.

From the heading error dynamics:

(77) $${\dot{e}}_{\psi i}=r_{i}-{\dot{\psi }}_{i}^{d}$$

The desired yaw angular velocity is designed as:

(78) $$r_{i}^{d}={\dot{\psi }}_{i}^{d}-\Phi_{\psi }\left(e_{\psi i}\right)$$

where $r_{i}^{d}$ denotes the desired yaw angular velocity of the USV and ${\dot{\psi }}_{i}^{d}$ denotes the time derivative of the desired heading angle.

Equation (77) yields:

(79) $${\dot{e}}_{\psi i}=-\Phi_{\psi }\left(e_{\psi i}\right)+\left(r_{i}-r_{i}^{d}\right)$$

The yaw angular velocity error is defined as:

(80) $$z_{\psi i}=r_{i}-r_{i}^{d}$$

Thus:

(81) $${\dot{e}}_{\psi i}=-c_{3}{\left|e_{\psi i}\right|}^{\alpha_{\psi }}\mathrm{sign}\left(e_{\psi i}\right)-c_{4}{\left|e_{\psi i}\right|}^{\beta_{\psi }}\mathrm{sign}\left(e_{\psi i}\right)+z_{\psi i}$$

Heading errors can be guaranteed to converge within a fixed time via the subsequent dynamic controller.

### 4.3. Disturbance Observer Design

In the USV dynamic model (21) presented in Section 2, $d_{i}$ represents unknown disturbances induced by wind, waves, and currents. Consistent with Assumption 3 in Section 2, marine environmental disturbances are bounded, i.e., a positive constant $d_{\max}$ exists such that $∥d_{i}∥\leq d_{\max}$. Meanwhile, parametric perturbations and hydrodynamic coefficient uncertainties exist in USV models. Unknown nonlinear dynamics and external disturbances are unified as comprehensive disturbances:

(82) $$\Delta_{i}=M_{i}^{-1}\left[-C_{i}\left(\nu_{i}\right)\nu_{i}-D_{i}\left(\nu_{i}\right)\nu_{i}+d_{i}\right]$$

The USV dynamic model is further rewritten as:

(83) $${\dot{\nu }}_{i}=M_{i}^{-1}\tau_{i}+\Delta_{i}$$

where $\Delta_{i}$ denotes unknown nonlinear terms jointly generated by model uncertainties and environmental disturbances.

Since accurate acquisition of $\Delta_{i}$ is infeasible in complex marine environments, a disturbance observer is designed to estimate it online.

**Lemma** **1.** *Consider the USV dynamic model with lumped disturbances given by Equation (83). Suppose Assumption 3 holds, and the time derivative of the disturbance is bounded by $∥{\dot{\Delta }}_{i}∥\leq {\bar{\Delta }}_{i}$. If the disturbance observer is designed as follows:*

(84) $${\hat{\Delta }}_{i}=z_{i}+\lambda_{i}\nu_{i}$$

(85) $${\dot{z}}_{i}=-\lambda_{i}\left(M_{i}^{-1}\tau_{i}+{\hat{\Delta }}_{i}\right)$$

*where ${\hat{\Delta }}_{i}$ denotes the estimated composite disturbance, $z_{i}$ is the auxiliary state variable of the observer, and $\lambda_{i}>0$ represents the observer gain, then the disturbance estimation error ${\tilde{\Delta }}_{i}=\Delta_{i}-{\hat{\Delta }}_{i}$ is uniformly ultimately bounded provided that $\lambda_{i}>1/2$.*

**Proof.**  The disturbance estimation error is defined as:

(86) $${\tilde{\Delta }}_{i}=\Delta_{i}-{\hat{\Delta }}_{i}$$

Differentiating Equation (86):

(87) $${\dot{\tilde{\Delta }}}_{i}={\dot{\Delta }}_{i}-{\dot{\hat{\Delta }}}_{i}$$

Differentiating Equation (84):

(88) $${\dot{\hat{\Delta }}}_{i}={\dot{z}}_{i}+\lambda_{i}{\dot{\nu }}_{i}$$

Substituting Equations (83) and (85) into Equation (88):

(89) $${\dot{\hat{\Delta }}}_{i}=-\lambda_{i}\left(M_{i}^{-1}\tau_{i}+{\hat{\Delta }}_{i}\right)+\lambda_{i}\left(M_{i}^{-1}\tau_{i}+\Delta_{i}\right)$$

Simplifying the expression:

(90) $${\dot{\hat{\Delta }}}_{i}=\lambda_{i}\left(\Delta_{i}-{\hat{\Delta }}_{i}\right)=\lambda_{i}{\tilde{\Delta }}_{i}$$

Thus:

(91) $${\dot{\tilde{\Delta }}}_{i}={\dot{\Delta }}_{i}-\lambda_{i}{\tilde{\Delta }}_{i}$$

Consistent with Assumption 3 in Section 2, the time derivative of environmental disturbances satisfies:

(92) $$∥{\dot{\Delta }}_{i}∥\leq {\bar{\Delta }}_{i}$$

A Lyapunov function is selected for stability analysis [29]:

(93) $$V_{\Delta }=\frac{1}{2}{\tilde{\Delta }}_{i}^{T}{\tilde{\Delta }}_{i}$$

Differentiating the Lyapunov function:

(94) $${\dot{V}}_{\Delta }={\tilde{\Delta }}_{i}^{T}{\dot{\Delta }}_{i}-\lambda_{i}{\tilde{\Delta }}_{i}^{T}{\tilde{\Delta }}_{i}$$

Applying Young’s inequality [30]:

(95) $${\tilde{\Delta }}_{i}^{T}{\dot{\Delta }}_{i}\leq \frac{1}{2}{∥{\tilde{\Delta }}_{i}∥}^{2}+\frac{1}{2}{∥{\dot{\Delta }}_{i}∥}^{2}$$

Therefore:

(96) $${\dot{V}}_{\Delta }\leq -\left(\lambda_{i}-\frac{1}{2}\right){∥{\tilde{\Delta }}_{i}∥}^{2}+\frac{1}{2}{\bar{\Delta }}_{i}^{2}$$

According to Lyapunov stability theory, if $\lambda_{i}>1/2$, ${\dot{V}}_{\Delta }<0$ is strictly guaranteed whenever $∥{\tilde{\Delta }}_{i}∥>{\bar{\Delta }}_{i}/\sqrt{2\lambda_{i}-1}$. This implies that the disturbance estimation error ${\tilde{\Delta }}_{i}$ converges to a bounded compact set $\Omega_{\tilde{\Delta }}=\left\{{\tilde{\Delta }}_{i}:∥{\tilde{\Delta }}_{i}∥\leq {\bar{\Delta }}_{i}/\sqrt{2\lambda_{i}-1}\right\}$. Consequently, by selecting a sufficiently large observer gain $\lambda_{i}$, the ultimate bound of the estimation error can be made arbitrarily small. The disturbance observer can thus effectively compensate for unknown model terms and marine disturbances, ensuring the robustness of the control system. □

### 4.4. Design of Fixed-Time Dynamic Control Law

The desired velocity state derived in Section 4.2 is:

(97) $$\nu_{i}^{d}={\left[u_{i}^{d},0,r_{i}^{d}\right]}^{T}$$

where $\nu_{i}^{d}$ denotes the desired velocity state vector of the *i*-th USV, $u_{i}^{d}$ denotes the desired longitudinal speed, $r_{i}^{d}={\dot{\psi }}_{i}^{d}$ denotes the desired yaw angular velocity, and the lateral velocity component is set to zero in accordance with the motion characteristics of underactuated USVs. This vector converts game-generated desired encirclement trajectories into executable dynamic control commands, laying a foundation for subsequent fixed-time dynamic controller design.

The velocity tracking error is defined as:

(98) $$z_{i}=\nu_{i}-\nu_{i}^{d}$$

where $z_{i}$ denotes the error between the actual velocity state and the desired velocity state.

Differentiating Equation (98):

(99) $${\dot{z}}_{i}={\dot{\nu }}_{i}-{\dot{\nu }}_{i}^{d}$$

Substituting Equation (83):

(100) $${\dot{z}}_{i}=M_{i}^{-1}\tau_{i}+\Delta_{i}-{\dot{\nu }}_{i}^{d}$$

To realize fixed-time convergence, a nonlinear fixed-time function is defined:

(101) $$\Phi \left(z_{i}\right)=k_{1}{\left|z_{i}\right|}^{m}\mathrm{sign}\left(z_{i}\right)+k_{2}{\left|z_{i}\right|}^{n}\mathrm{sign}\left(z_{i}\right)$$

where $k_{1},k_{2}>0$ denote the control gains; exponents satisfy 0<m<1,n>1. The power term with exponent less than 1 enhances convergence performance under small errors, while the power term with exponent greater than 1 accelerates adjustment under large initial errors.

The fixed-time trajectory tracking control input is designed as:

(102) $$\tau_{i}^{0}=M_{i}\left[-{\hat{\Delta }}_{i}+{\dot{\nu }}_{i}^{d}-\Phi \left(z_{i}\right)\right]$$

where $\tau_{i}^{0}$ denotes the fixed-time trajectory tracking control input.

Substituting Equation (102) into Equation (100):

(103) $${\dot{z}}_{i}=-\Phi \left(z_{i}\right)+{\tilde{\Delta }}_{i}$$

i.e.,:

(104) $${\dot{z}}_{i}=-k_{1}{\left|z_{i}\right|}^{m}\mathrm{sign}\left(z_{i}\right)-k_{2}{\left|z_{i}\right|}^{n}\mathrm{sign}\left(z_{i}\right)+{\tilde{\Delta }}_{i}$$

When the disturbance estimation error satisfies:

(105) $${\tilde{\Delta }}_{i}\to 0$$

the system satisfies:

(106) $${\dot{z}}_{i}=-k_{1}{\left|z_{i}\right|}^{m}\mathrm{sign}\left(z_{i}\right)-k_{2}{\left|z_{i}\right|}^{n}\mathrm{sign}\left(z_{i}\right)$$

Therefore, the velocity error converges within a fixed time.

### 4.5. Safety Constraint Design Based on Control Barrier Functions

Fixed-time control guarantees fast tracking performance of USVs but cannot inherently satisfy inter-USV safe distance constraints during swarm encirclement. Therefore, control barrier functions are introduced to enforce safety constraints.

The safety function between the *i*-th and *j*-th USVs is defined as:

(107) $$h_{ij}={∥p_{i}-p_{j}∥}^{2}-d_{s}^{2}$$

where $d_{s}$ denotes the minimum allowable safe distance between USVs. $h_{ij}\geq 0$ indicates that the safety constraint is satisfied between the two USVs.

In accordance with control barrier function theory, the following inequality must hold:

(108) $${\dot{h}}_{ij}+\gamma h_{ij}\geq 0$$

where γ>0 denotes the adjustment parameter for safety constraints.

Differentiating Equation (107):

(109) $${\dot{h}}_{ij}=2{\left(p_{i}-p_{j}\right)}^{T}\left({\dot{p}}_{i}-{\dot{p}}_{j}\right)$$

Safety constraints are converted into restrictions on control inputs:

(110) $$A_{ij}\tau_{i}+b_{ij}\geq 0$$

where $A_{ij}=\frac{\partial h_{ij}}{\partial p_{i}}\frac{\partial p_{i}}{\partial \tau_{i}}$ characterizes the influence of control inputs on the time derivative of the safety function.

A safety correction control term is designed as:

(111) $$\tau_{i}^{s}=-\rho_{i}\nabla h_{ij}$$

where $\rho_{i}>0$ denotes the safety gain.

Combining Equations (102) and (111), the final control input of the USV is designed as:

(112) $$\tau_{i}=\tau_{i}^{0}+\tau_{i}^{s}$$

where $\tau_{i}^{0}$ denotes the fixed-time trajectory tracking control input and $\tau_{i}^{s}$ denotes the collision avoidance safety correction input. This control structure balances encirclement tracking performance and inter-USV safe distance maintenance throughout swarm motion.

### 4.6. Stability Proof

Lyapunov stability theory is adopted to verify the effectiveness of the proposed fixed-time safety controller for dynamic target encirclement by USV swarms. Three core control objectives are validated: first, position and heading tracking errors of USVs converge within fixed time to track game-generated desired encirclement trajectories rapidly; second, velocity errors remain stable and convergent under model uncertainties and marine environmental disturbances; third, control barrier functions maintain safety distance constraints during swarm motion.

**Theorem** **2.** *Under Assumptions 2–5 presented in Section 2, applying the control law (112) to the USV dynamic system (21) ensures fixed-time convergence of closed-loop position, heading, and velocity errors to zero. Meanwhile, the safety constraint set defined by control barrier functions remains positively invariant, and the USV swarm consistently satisfies inter-vessel safe distance constraints.*

**Proof.**  First, a comprehensive error state is defined:

(113) $$\chi_{i}={\left[e_{i}^{T},e_{\psi_{i}},z_{i}^{T}\right]}^{T}$$

where $e_{i}$ denotes USV position tracking error, $e_{\psi_{i}}$ denotes heading tracking error, and $z_{i}$ denotes velocity tracking error.

A Lyapunov function is selected:

(114) $$V_{i}=\frac{1}{2}e_{i}^{T}e_{i}+\frac{1}{2}e_{\psi_{i}}^{2}+\frac{1}{2}z_{i}^{T}z_{i}$$

The first term characterizes position error energy, the second term characterizes heading error energy, and the third term characterizes velocity error energy. Since $V_{i}\geq 0$ and $V_{i}=0$ if and only if $e_{i}=0$, $e_{\psi_{i}}=0$, $z_{i}=0$, Equation (114) satisfies positive definiteness.

(1) Stability Analysis of Position Errors

Based on the fixed-time position controller designed in Section 4.2:

(115) $${\dot{e}}_{i}=-c_{1}{\left|e_{i}\right|}^{\alpha }\mathrm{sign}\left(e_{i}\right)-c_{2}{\left|e_{i}\right|}^{\beta }\mathrm{sign}\left(e_{i}\right)$$

where $c_{1},c_{2}$ are position control gains, while α,β denote fixed-time control exponents.

The Lyapunov term corresponding to position errors in Equation (114) is:

(116) $$V_{ei}=\frac{1}{2}e_{i}^{T}e_{i}$$

Its time derivative is:

(117) $${\dot{V}}_{ei}=e_{i}^{T}{\dot{e}}_{i}$$

Substituting Equation (115) into Equation (117):

(118) $${\dot{V}}_{ei}=-c_{1}e_{i}^{T}{\left|e_{i}\right|}^{\alpha }\mathrm{sign}\left(e_{i}\right)-c_{2}e_{i}^{T}{\left|e_{i}\right|}^{\beta }\mathrm{sign}\left(e_{i}\right)$$

Since $e_{i}^{T}\mathrm{sign}\left(e_{i}\right)=∥e_{i}∥$, we have:

(119) $${\dot{V}}_{ei}\leq -c_{1}{∥e_{i}∥}^{1+\alpha }-c_{2}{∥e_{i}∥}^{1+\beta }$$

Given 0<α<1, β>1, position errors satisfy fixed-time stability conditions.

(2) Stability Analysis of Heading Errors

The Lyapunov function for heading errors is:

(120) $$V_{\psi i}=\frac{1}{2}e_{\psi i}^{2}$$

Differentiating:

(121) $${\dot{V}}_{\psi i}=e_{\psi i}{\dot{e}}_{\psi i}$$

Substituting the heading controller (81) derived in Section 4.2:

(122) $${\dot{V}}_{\psi i}\leq -c_{3}{\left|e_{\psi i}\right|}^{1+\alpha_{\psi }}-c_{4}{\left|e_{\psi i}\right|}^{1+\beta_{\psi }}+e_{\psi i}z_{\psi i}$$

where $e_{\psi i}z_{\psi i}$ denotes the coupling term between heading error and angular velocity error.

(3) Stability Analysis of Velocity Errors

The Lyapunov term corresponding to velocity errors is:

(123) $$V_{zi}=\frac{1}{2}z_{i}^{T}z_{i}$$

Differentiating:

(124) $${\dot{V}}_{zi}=z_{i}^{T}{\dot{z}}_{i}$$

Substituting Equation (104):

(125) $${\dot{V}}_{zi}=-k_{1}{∥z_{i}∥}^{m+1}-k_{2}{∥z_{i}∥}^{n+1}+z_{i}^{T}{\tilde{\Delta }}_{i}$$

Based on the disturbance observer design in Section 4.3, the disturbance estimation error satisfies ${\tilde{\Delta }}_{i}\to 0$. Therefore:

(126) $$z_{i}^{T}{\tilde{\Delta }}_{i}\leq \varepsilon_{i}$$

where $\varepsilon_{i}$ stands for the upper bound of the disturbance estimation error. Therefore:

(127) $${\dot{V}}_{zi}\leq -k_{1}{∥z_{i}∥}^{m+1}-k_{2}{∥z_{i}∥}^{n+1}+\varepsilon_{i}$$

(4) Comprehensive Stability Analysis

The time derivative of the overall Lyapunov function (114) is:

(128) $${\dot{V}}_{i}={\dot{V}}_{ei}+{\dot{V}}_{\psi i}+{\dot{V}}_{zi}$$

Combining Equations (119), (122) and (127):

(129) $${\dot{V}}_{i}\leq -a_{1}V_{i}^{\mu }-a_{2}V_{i}^{\nu }+\varepsilon_{i}$$

where $a_{1},a_{2}>0$ denote integrated control gains; μ,ν denote fixed-time convergence exponents satisfying 0<μ<1,ν>1; $\varepsilon_{i}\to 0$ denotes the upper bound of integrated disturbance errors, which approaches zero as the disturbance estimation error converges. Thus:

(130) $${\dot{V}}_{i}\leq -a_{1}V_{i}^{\mu }-a_{2}V_{i}^{\nu }$$

In accordance with fixed-time stability theory, the system convergence time satisfies:

(131) $$T_{f}\leq \frac{1}{a_{1}\left(1-\mu \right)}+\frac{1}{a_{2}\left(\nu -1\right)}$$

Therefore:

(132) $$\lim_{t\to T_{f}}V_{i}\left(t\right)=0$$

i.e.,

(133) $$\lim_{t\to T_{f}}\left(e_{i},e_{\psi i},z_{i}\right)=0$$

This proves that USVs realize tracking convergence of position, heading, and velocity states within fixed time.

(5) Proof of Safety Constraints

Based on Equation (107) and control barrier function theory, the safety set:

(134) $$\Omega_{s}=\left\{x:h_{ij}\geq 0\right\}$$

is positively invariant.

Thus:

(135) $$h_{ij}\left(t\right)\geq 0$$

is always satisfied, i.e.,

(136) $$∥p_{i}-p_{j}∥\geq d_{s}$$

meaning the distance between any two USVs consistently exceeds the minimum safe distance $R_{s}$, eliminating collision risks during swarm motion.

In summary, the proposed USV swarm encirclement method combining dynamic game and fixed-time safety control guarantees fixed-time stability of the closed-loop system while satisfying inter-vessel safety distance constraints throughout the encirclement process. The theorem is proven. □

## 5. Numerical Simulations

To validate the effectiveness of the proposed encirclement method based on dynamic games and fixed-time safety control, numerical simulations are carried out on the MATLAB platform. The simulation environment runs on the Windows 11 operating system, equipped with an Intel Core i9-14900K CPU (3.2 GHz main frequency), 32 GB RAM and an NVIDIA GeForce RTX 4070 graphics card with 12 GB video memory. All algorithms are programmed with MATLAB R2023b using double-precision floating-point arithmetic. The total simulation duration is set to 500 s with a time step of 0.1 s.

Dynamic encirclement simulations under ocean current disturbances are conducted in this paper. In the simulation setup, the target vessel acts as an evasive agent that adjusts its motion according to its own reward function, while pursuit USVs derive optimal encirclement strategies via the dynamic game model and rapidly track the desired encirclement positions using the fixed-time safety controller.

### 5.1. Parameter Settings

The well-known CyberShip model referenced in existing literature is adopted for simulation [28]. Its length L=1.255m and beam W=0.29m are consistent with standard experimental vessels. The key model parameters are listed as follows:

(1) Inertia matrix coefficients: $m_{11}=25.8\;\mathrm{kg}$, $m_{22}=33.8\;\mathrm{kg}$, $m_{33}=2.76\;\mathrm{kg}\cdot m^{2}$.
(2) Linear damping coefficients: $d_{11}=12\;\mathrm{kg}/s$, $d_{22}=17\;\mathrm{kg}/s$, $d_{33}=0.5\;\mathrm{kg}\cdot m^{2}/s$.

The simulation scenario is configured with four USVs cooperatively encircling one target vessel. The initial position of the target is set as: $P_{T}\left(0\right)=\left(0,0\right)$, and the initial positions of the USVs are assigned as follows:

$$\left\{\begin{array}{c} P_{1}=\left(-120,100\right)\\ P_{2}=\left(-120,-100\right)\\ P_{3}=\left(120,-100\right)\\ P_{4}=\left(0,-100\right) \end{array}\right.$$

The fixed-time safety control scheme proposed in Section 4 is adopted for the USV controllers. The fixed-time parameters, safety constraint parameters and control input limit parameters are summarized in Table 2.

The weight coefficients for the dynamic game payoff functions are set in Table 3.

A composite marine disturbance term is introduced to replicate real-world ocean current, wave and random turbulence effects on USV kinematics. The disturbance model is formulated as

(137) $$\vec{d}\left(t\right)=d_{c}\left(t\right)+d_{w}\left(t\right)+d_{r}\left(t\right)$$

where $d_{c}\left(t\right)$ denotes steady ocean current disturbance, $d_{w}\left(t\right)$ denotes periodic wave disturbance, and $d_{r}\left(t\right)$ denotes stochastic marine disturbance.

The steady ocean current component is configured as

(138) $${\vec{d}}_{c}=V_{c}\left[\begin{array}{c} \cos\beta_{c}\\ \sin\beta_{c} \end{array}\right]$$

where $V_{c}$ represents ocean current velocity components, and $\beta_{c}$ denotes the ocean current direction. In this paper, we set $V_{c}=0.25$, $\beta_{c}=\frac{\pi }{6}$.

The periodic wave disturbance takes the sinusoidal form

(139) $${\vec{d}}_{w}=A_{w}\left[\begin{array}{c} \sin(\omega_{w}t)\\ \cos(\omega_{w}t) \end{array}\right]$$

where $A_{w}=0.12$, $\omega_{w}=0.08$.

The random marine disturbance term is defined as

(140) $${\vec{d}}_{r}=A_{r}\left[\begin{array}{c} \xi_{x}\\ \xi_{y} \end{array}\right]$$

where $\xi_{x},\xi_{y}$ follows the standard normal distribution and $A_{r}=0.02$.

**Figure 3:**
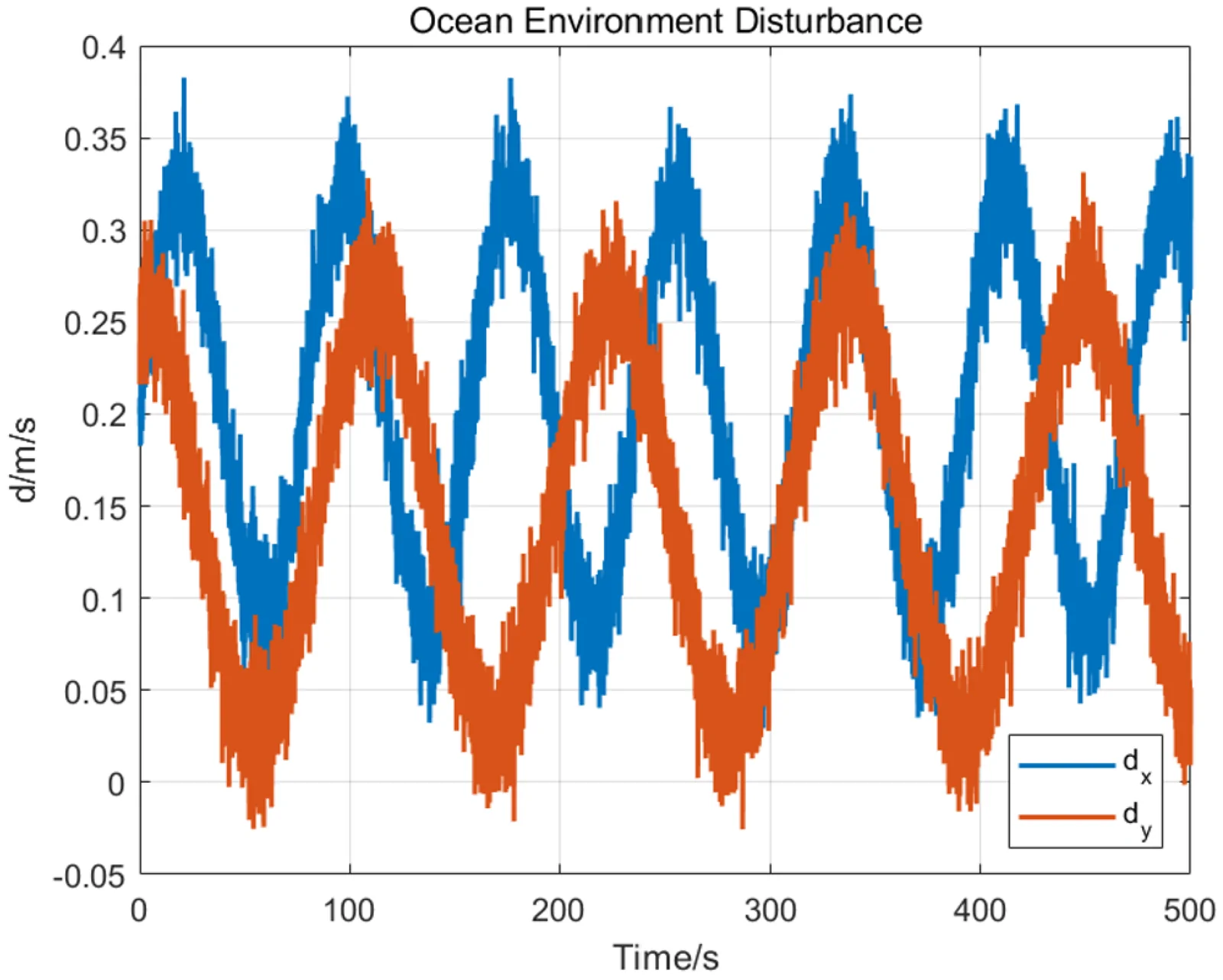
Schematic Diagram of Ocean Current Disturbances.

The overall disturbance magnitude ∥d(t)∥<0.5m/s satisfies the boundedness condition stated in Assumption 2. The time-domain curve of ocean current disturbances is illustrated in Figure 3.

### 5.2. Simulation Analysis

Dynamic target encirclement simulations under ocean current disturbances are implemented to verify the robustness and efficacy of the proposed integrated method. Throughout the simulation, the target vessel continuously modulates its motion to maximize escape rewards, while each pursuit USV updates its desired encirclement positions in real time via dynamic game iteration and achieves safe, fast tracking through the fixed-time safety control framework.

**Figure 4:**
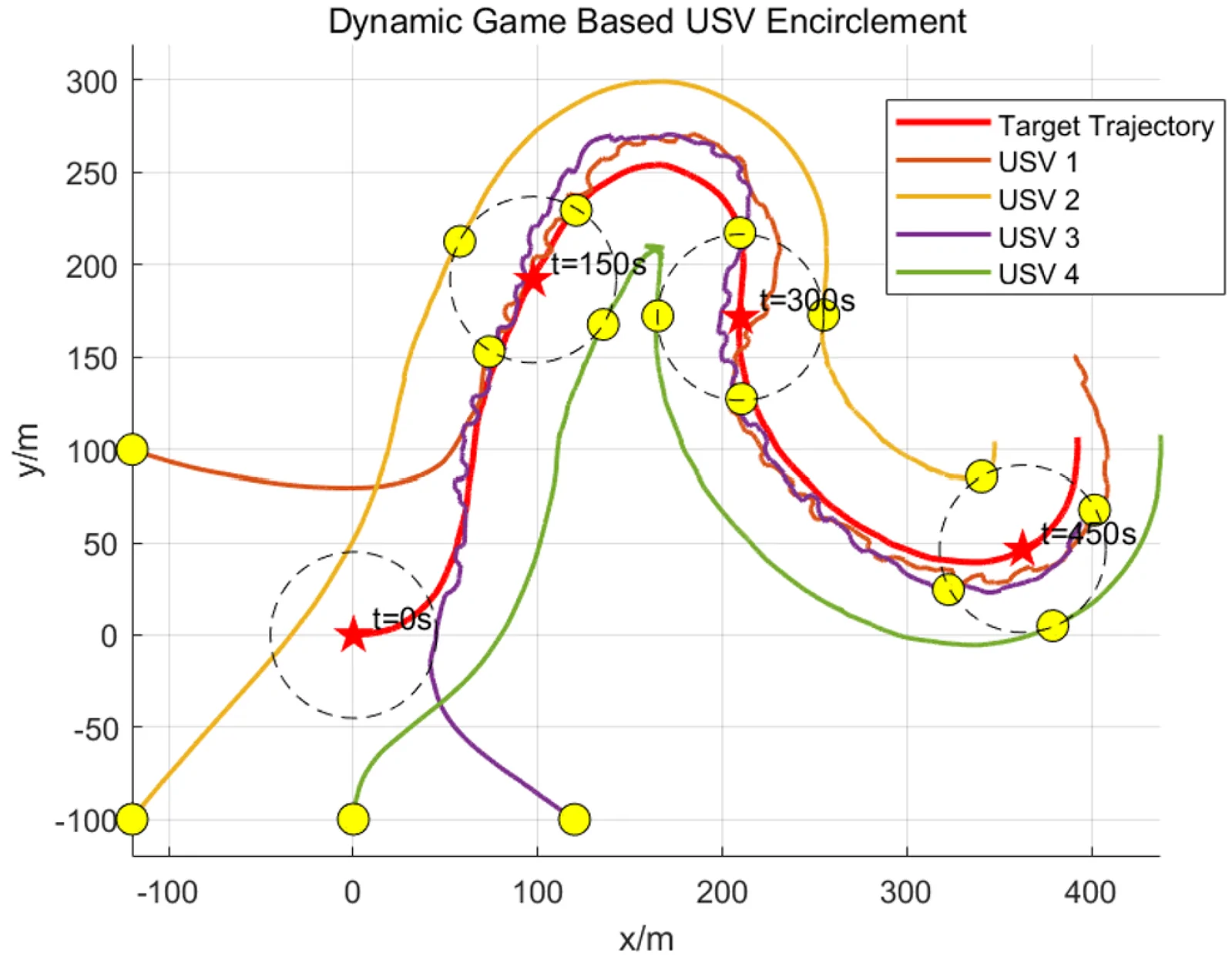
Trajectories of the Pursuers and Evader During the Encirclement Process. Yellow circles and red stars mark the positions of the pursuers and evader, respectively, at t=0, 150, 300, and 450 s. Black dashed circles indicate the desired encirclement circles centered on the evader at these times.

The trajectory plot in Figure 4 visualizes the swarm encirclement process under ocean current interference. At the initial instant, all four USVs are far away from the target, with the maximum initial separation distance exceeding 150 m. As the dynamic game strategies iterate continuously, each USV adjusts its trajectory according to the target’s motion trend and gradually converges toward the encirclement zone.

The trajectory evolution reveals distinct stages: from 0 s to 100 s, USVs rapidly close the distance to the target and complete preliminary position adjustment; from 100 s to 150 s, the swarm dynamically rearranges and forms a stable closed formation surrounding the target; after 150 s, the relative motion between the target and USVs maintains steady encirclement geometry. Marked snapshots at 150 s, 300 s and 450 s demonstrate that the target remains confined at the geometric center of the USV formation. Even at 450 s, the target cannot break through the encirclement zone formed by four USVs, which proves that the dynamic game strategy adapts to sustained target maneuvers and enables long-term stable containment. Meanwhile, the encirclement trajectories exhibit distinct biomimetic cooperative characteristics. Multiple USVs can autonomously adjust their encirclement angles and continuously shrink the escape space of the target, which is consistent with the group predation behaviors observed in nature.

**Figure 5:**
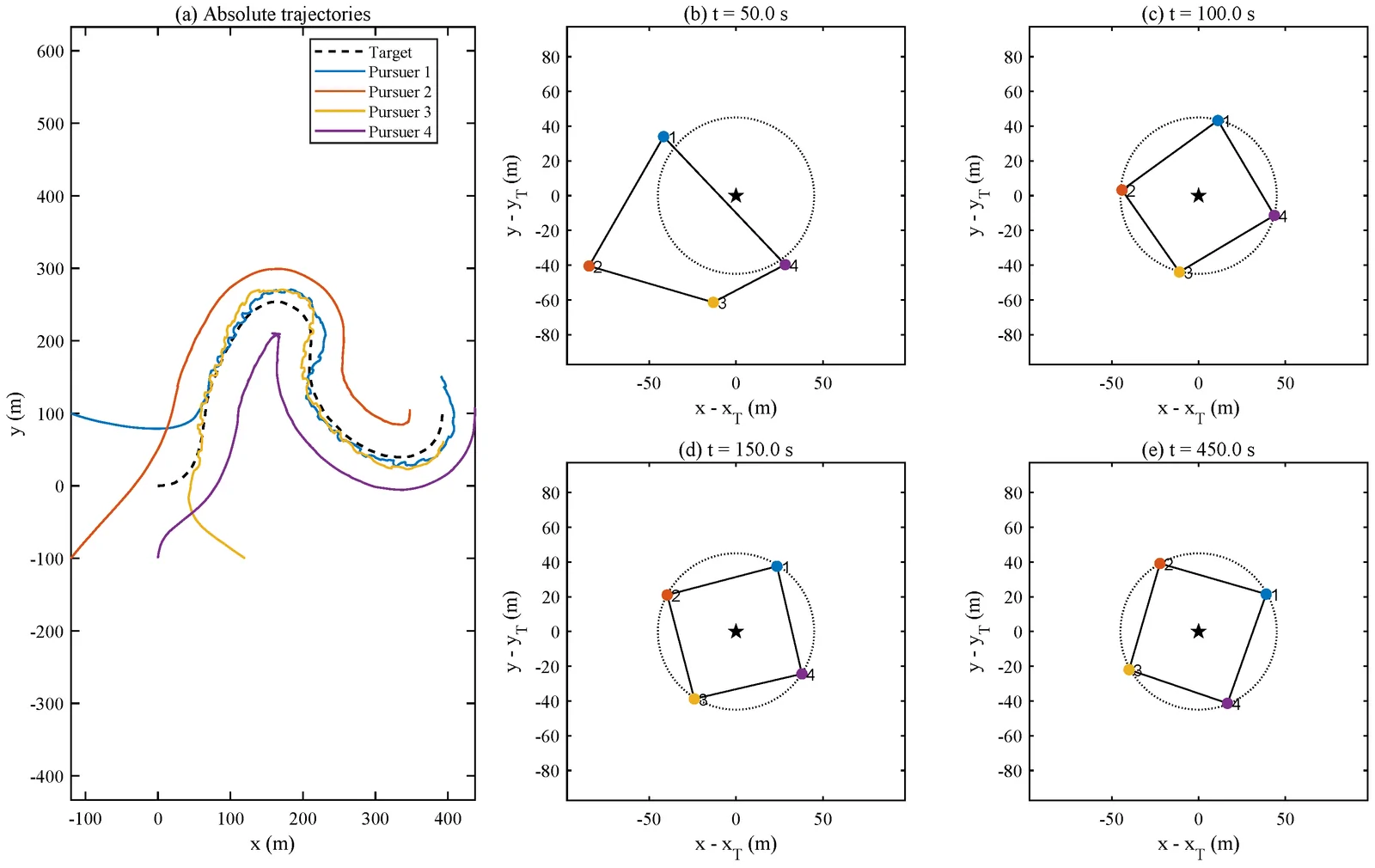
Encirclement trajectories and representative configurations: (**a**) absolute trajectories; (**b**–**e**) target-centered snapshots at 50, 100, 150, and 450s. Stars denote the target. Colored circular markers denote the positions of the pursuing unmanned surface vehicles, with numbers identifying individual pursuers and colors matching their trajectories in panel (**a**). Dotted circles indicate the desired encirclement radius of 45m, and solid polygons outline the pursuer configurations.

To further illustrate the evolution of the encirclement geometry, Figure 5 presents the absolute trajectories and target-centered configurations at four representative instants. At 50s, the pursuers are still adjusting their relative positions. By the snapshot at 100s, the four pursuers are distributed around the target near the desired circle. The configurations at 150s and 450s also exhibit a nearly square encirclement geometry, with the target located inside the polygon formed by the pursuers. These results illustrate the formation adjustment and encirclement maintenance during target maneuvering.

**Figure 6:**
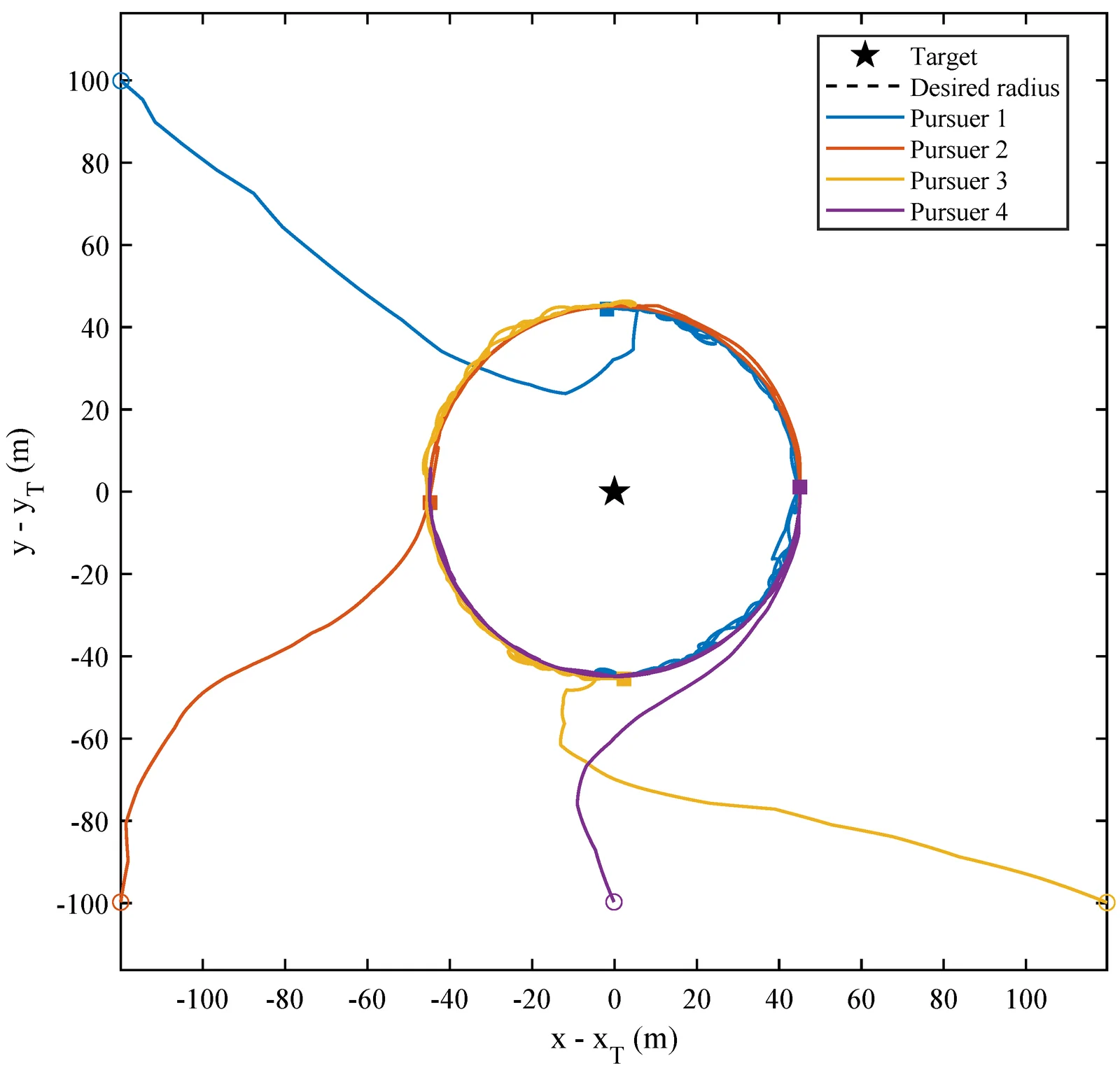
Target -relative trajectories of the pursuing unmanned surface vehicles. The dashed circle indicates the desired encirclement radius of 45m. Open circles and filled squares denote the first and last recorded positions, respectively.

Figure 6 further presents the pursuer trajectories relative to the target. After the initial approach, the relative trajectories concentrate around the desired circle with a radius of 45m. Despite changes in the target heading and environmental disturbances, the pursuers adjust their angular positions while remaining close to the prescribed encirclement radius. Together with the snapshots in Figure 5, these results demonstrate the ability to establish and maintain the desired encirclement geometry in the simulated maneuvering-target scenario.

**Figure 7:**
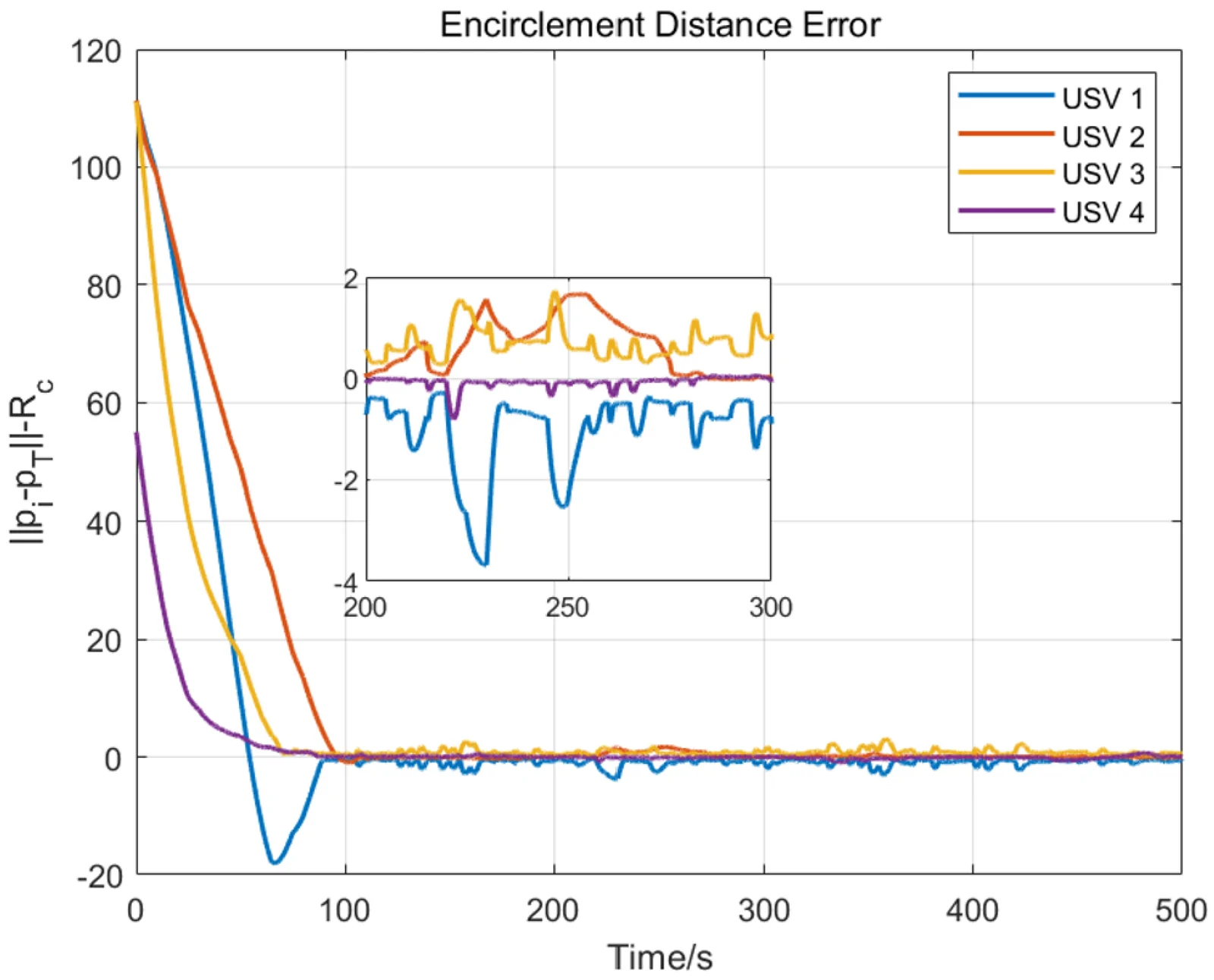
Variation Curves of Encirclement Distance Error.

Figure 7 plots the time-varying encirclement distance error curves. At the initial stage, the maximum distance error reaches approximately 100 m. Driven by the fixed-time controller, the error decays rapidly. USV4 converges to the stable error band within roughly 40 s due to its short initial distance to the target, while USV1, USV2 and USV3 finish formation adjustment within 80–100 s. After 100 s, the steady-state error fluctuates within ±2 m. The partial enlarged view shows transient fluctuations smaller than 4 m induced by ocean currents and target maneuvers, which decay rapidly without sustained divergence.

**Figure 8:**
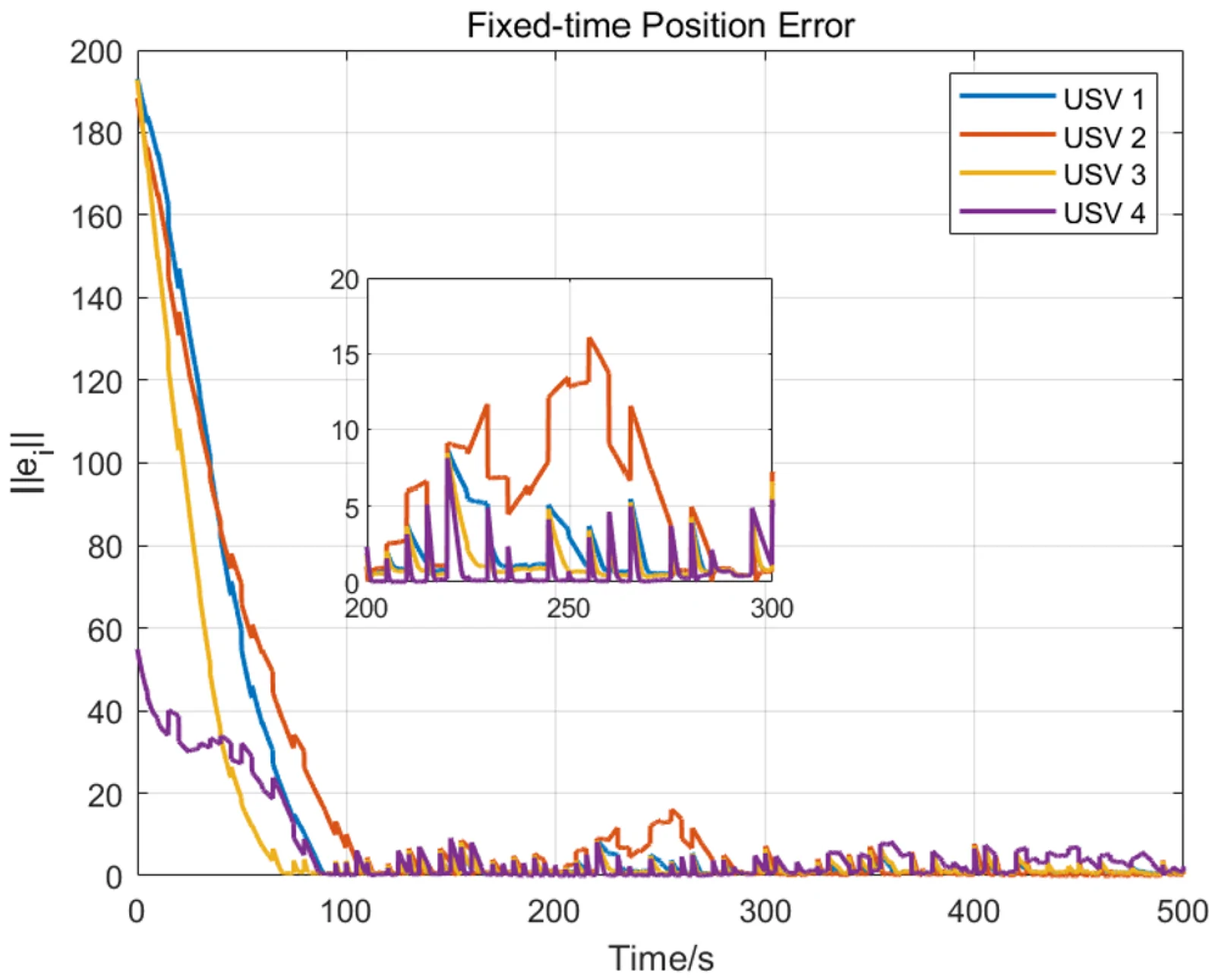
Variation Curves of Position Tracking Errors for USVs.

Figure 8 presents the position tracking error curves of each USV. The maximum initial position error approaches 190m, yet over 90% of the error diminishes within the first 80s. After 100s, the tracking error is confined within 10m, with steady-state fluctuations limited to 5m. USV2 exhibits moderate transient deviations caused by abrupt target heading changes, but no unbounded error growth is observed. The results validate the fast convergence and strong anti-disturbance capability of the fixed-time control scheme for trajectories generated by dynamic games.

**Figure 9:**
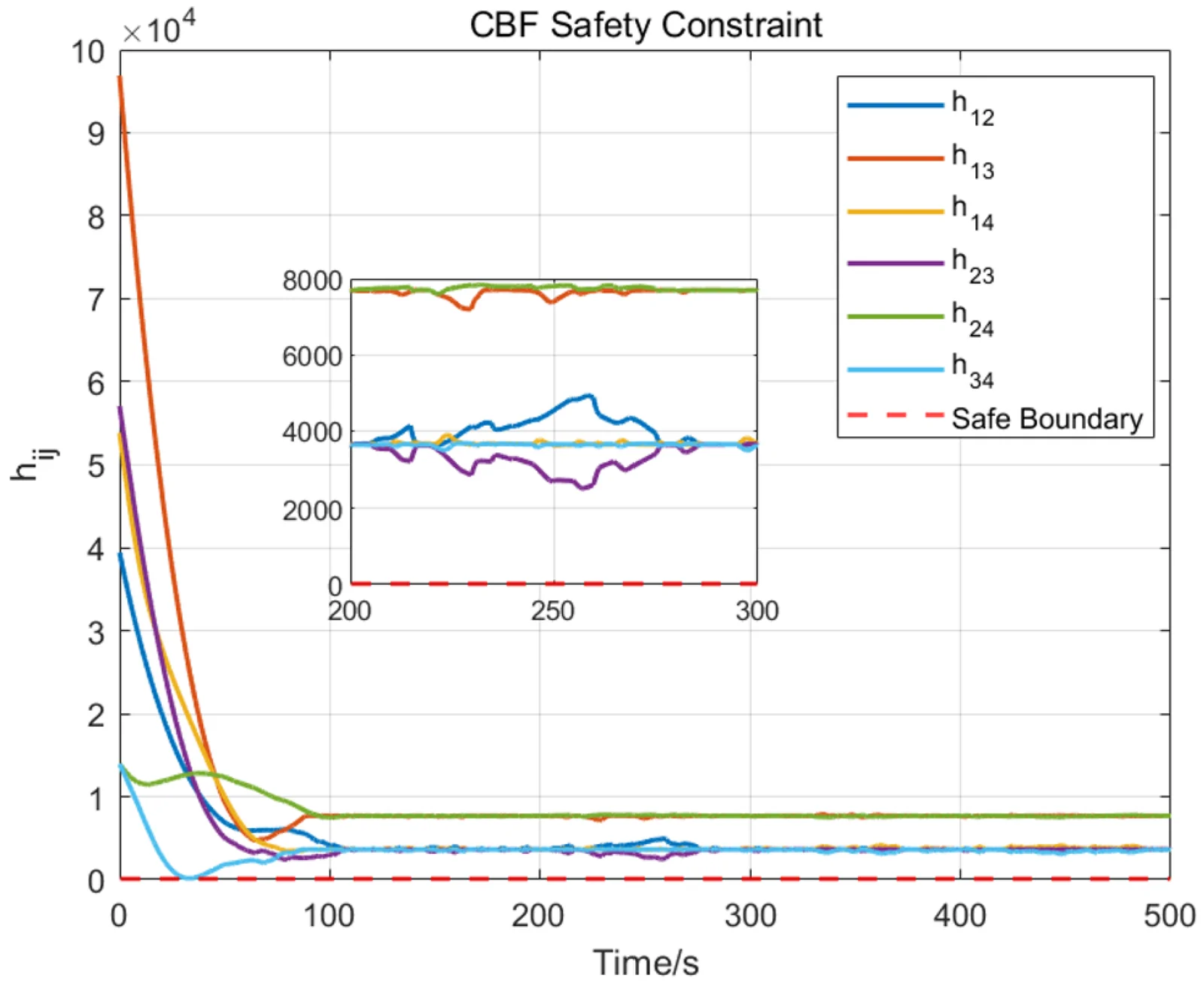
Variation Curves of Safety Constraints.

Figure 9 illustrates the variation curves of safety constraints based on control barrier functions. It can be seen that all safety functions remain positive throughout the simulation. At the initial stage, the USVs are widely distributed, resulting in relatively high values of each safety function, with the maximum safety margin exceeding $8\times 10^{4}$. As the USVs gradually approach the target and form an encirclement formation, the safety functions decrease and tend to be stable. In the steady stage, the safety functions between USVs are maintained within the range of $3\times 10^{3}\;∼\;8\times 10^{3}$, which means the actual distances are always larger than the preset safe distance. The partial enlarged view shows that during 200–300s, the safety functions fluctuate to a certain extent under the influence of target maneuvering and ocean current disturbances. However, the safety functions never approach zero or become negative. The results verify that the control barrier function can effectively guarantee the motion safety of the USV swarm.

**Figure 10:**
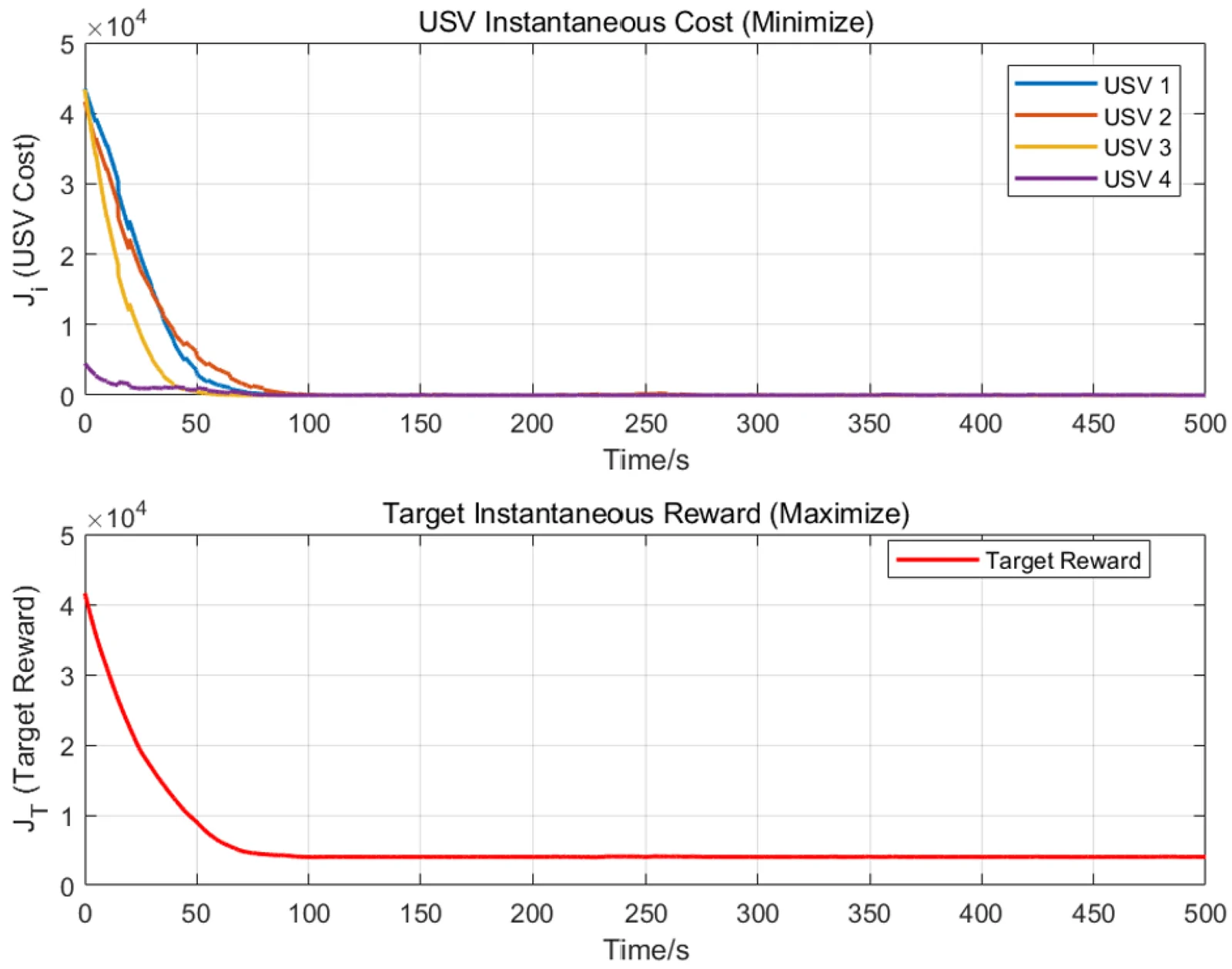
Variation Curves of USV Pursuit Payoff and Target Escape Payoff.

Figure 10 presents the variation curves of pursuit payoff for USVs and escape payoff for the target. For the pursuing USVs, the instantaneous cost function maintains a high value at the initial stage, with a maximum value of approximately $4\times 10^{4}$. As the USVs gradually approach the target and complete formation adjustment, the cost declines rapidly. After roughly 80s, the cost drops to a small range and remains stable thereafter. This indicates that the dynamic game strategy can effectively reduce the position error and control consumption during the USV encirclement process.

For the target vessel, its payoff function is approximately $4.2\times 10^{4}$ at the initial stage. The target improves its escape payoff by adjusting the motion direction. With the continuous strategy adjustment of the pursuing USVs, the target payoff gradually decreases and stabilizes near $4\times 10^{3}$. At the beginning, the target elevates its escape payoff by tuning the heading. As the pursuing USVs constantly update their strategies, the target payoff gradually converges, which demonstrates that a dynamic game equilibrium is formed between the target and the pursuing USVs. Once the encirclement pattern is established, the target can no longer increase its escape payoff. The above results reveal that the proposed method achieves encirclement not merely via trajectory tracking. Instead, it constructs a dynamic strategy interaction between pursuers and evaders, enabling the USVs to actively adjust the encirclement scheme according to variations in target behaviors.

**Figure 11:**
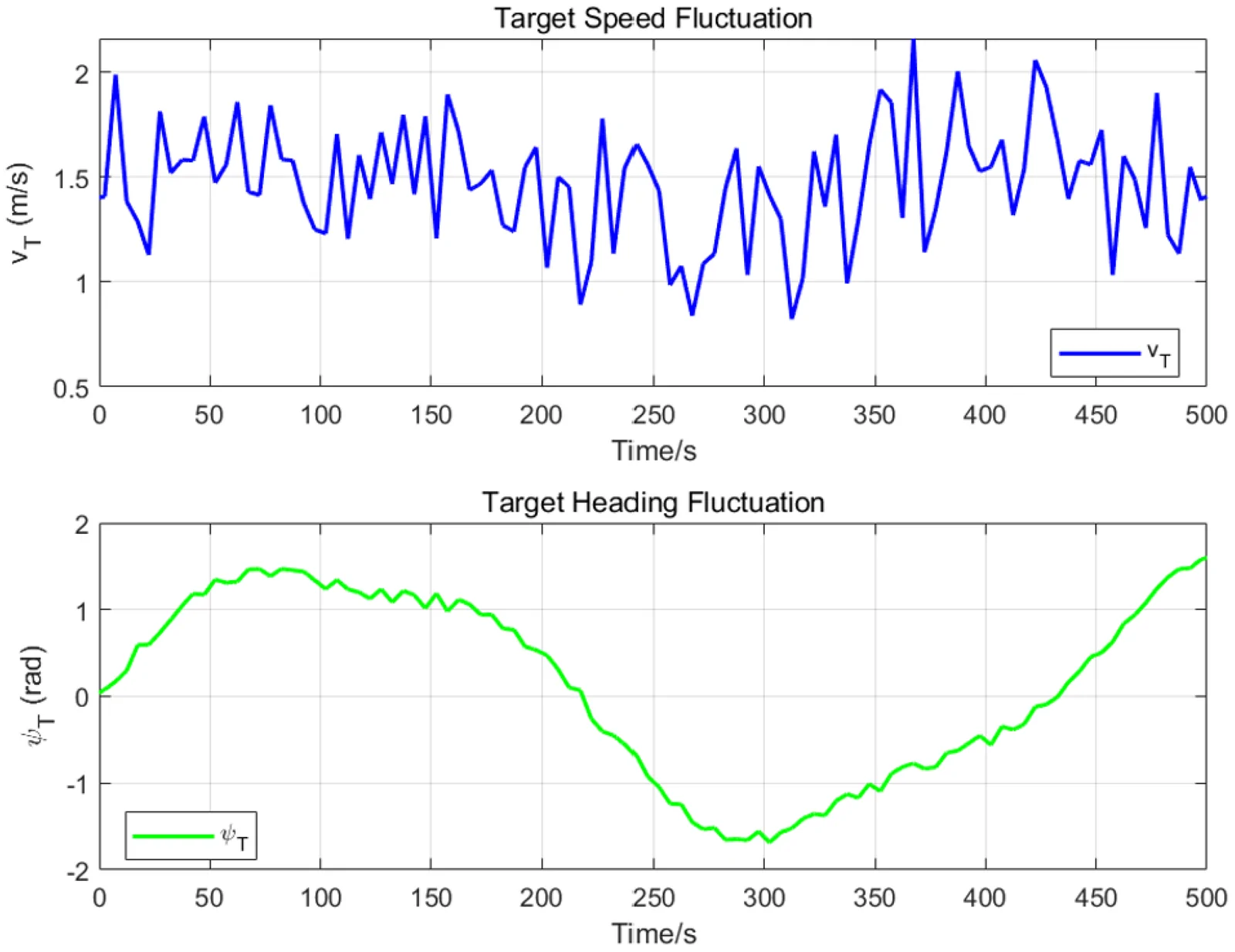
Variation Curves of Target Velocity and Heading.

Figure 11 displays the target’s time-varying speed and heading profiles. The target velocity fluctuates between 0.8m/s and 2.2m/s, while its heading angle varies within the range of −1.7rad to 1.6rad over the full simulation duration. These curves verify that the target executes continuous, unplanned evasive maneuvers rather than following a predetermined fixed path. Nevertheless, the USV swarm predicts the target’s motion tendency via dynamic game iteration and maintains effective spatial confinement. As shown in the trajectory diagram, the target fails to escape the encirclement perimeter despite frequent velocity and heading adjustments. Target maneuvers only induce mild rearrangement of the USV formation without breaking the overall containment structure.

Collectively, the dynamic encirclement simulation under ocean current disturbances leads to the following conclusions: the proposed method achieves stable swarm containment against continuously maneuvering targets and persistent environmental turbulence. The USV swarm establishes effective encirclement within approximately 100s, with steady-state distance errors controlled within ±2m; all safety functions maintain positive values throughout the process, and the payoffs of both pursuers and evaders converge to equilibrium levels. These outcomes verify the validity and strong robustness of the combined dynamic game and fixed-time safety control framework in complex marine environments.

### 5.3. Comparison with Baseline Methods

#### 5.3.1. Evaluation Metrics

To evaluate the encirclement performance against a maneuvering target, the proposed method is compared with consensus formation control, leader–follower control, an artificial potential field method, nonlinear encirclement, and predictive encirclement. All methods are evaluated using identical encirclement criteria in terms of encirclement establishment time, steady-state radial error, minimum inter-vessel distance, safety constraint satisfaction rate, encirclement retention rate, and control effort.

Let *N* denote the number of USVs, Δt the sampling interval, and $t_{k}=k\Delta t$ the sampling instants, where k=0,…,K−1. The positions of USV *i* and the target are denoted by $p_{i}\left(t_{k}\right)$ and $p_{T}\left(t_{k}\right)$, respectively. For the desired encirclement radius $R_{c}$, the radial error is defined as

$$e_{r,i}\left(t_{k}\right)=∥p_{i}\left(t_{k}\right)-p_{T}\left(t_{k}\right)∥-R_{c}.$$

*Encirclement establishment time:* Encirclement is evaluated using both radial accuracy and geometric enclosure. A sampling instant satisfies the encirclement condition when all radial errors lie within the prescribed tolerance and the target is strictly inside the convex hull of the USV positions:

$$C_{k}=\left\{\begin{array}{cc} 1, & \begin{array}{c} \max_{1\leq i\leq N}\left|e_{r,i}\left(t_{k}\right)\right|\leq \varepsilon_{r},\\ p_{T}\left(t_{k}\right)\in \mathrm{int}\mathrm{conv}\left\{p_{1}\left(t_{k}\right),\ldots ,p_{N}\left(t_{k}\right)\right\}, \end{array}\\ 0, & \mathrm{otherwise}. \end{array}\right.$$

Here, $\varepsilon_{r}$ is the radial tolerance, and intconv{·} denotes the interior of the convex hull. To exclude transient entry into the encirclement region, the condition must remain satisfied for a holding duration $T_{h}$. With $L=T_{h}/\Delta t$, the establishment time is defined as

$$k_{\mathrm{enc}}=\min\left\{k\in \left\{0,\ldots ,K-1-L\right\}:C_{k+\ell }=1,\;\ell =0,\ldots ,L\right\},\;T_{\mathrm{enc}}=t_{k_{\mathrm{enc}}}.$$

The primary criterion uses $\varepsilon_{r}=5\;m$ and $T_{h}=10\;s$. The reported establishment time is the beginning of the first qualifying interval, while confirmation occurs at $T_{\mathrm{enc}}+T_{h}$. If no qualifying interval exists during the simulation, establishment is reported as not achieved. An additional strict criterion uses a radial tolerance of 2m and a holding duration of 20s, with the corresponding establishment time denoted by $T_{\mathrm{enc}}^{\mathrm{strict}}$.

*Steady-state radial root-mean-square error:* The final 100s, corresponding to 400–500s, is selected as the steady-state evaluation interval. Let $K_{\mathrm{ss}}$ denote its sampling indices. The steady-state radial root-mean-square error is

$$E_{\mathrm{ss}}=\sqrt{\frac{1}{N|K_{\mathrm{ss}}|}\sum_{k\in K_{\mathrm{ss}}}\sum_{i=1}^{N}e_{r,i}^{2}\left(t_{k}\right)}.$$

A smaller value indicates more accurate regulation of the encirclement radius. The instantaneous radial root-mean-square error and maximum absolute radial error are also calculated to characterize the transient response:

$$E_{r}\left(t_{k}\right)=\sqrt{\frac{1}{N}\sum_{i=1}^{N}e_{r,i}^{2}\left(t_{k}\right)},\;E_{\max}\left(t_{k}\right)=\max_{1\leq i\leq N}\left|e_{r,i}\left(t_{k}\right)\right|.$$

*Minimum inter-vessel distance and safety satisfaction rate:* The minimum inter-vessel distance is evaluated over all USV pairs and sampling instants:

$$d_{\min}=\min_{\begin{array}{c} 0\leq k\leq K-1\\ 1\leq i<j\leq N \end{array}}∥p_{i}\left(t_{k}\right)-p_{j}\left(t_{k}\right)∥.$$

For the safety distance $d_{s}$, the safety satisfaction rate is defined as the percentage of sampling instants at which every pairwise distance satisfies the safety requirement:

$$S_{\mathrm{safe}}=\frac{100\%}{K}\sum_{k=0}^{K-1}I\left[\min_{i<j}∥p_{i}\left(t_{k}\right)-p_{j}\left(t_{k}\right)∥\geq d_{s}\right],$$

where I[·] denotes the indicator function. The safety distance is set to $d_{s}=20\;m$, and safety performance is reported separately from encirclement establishment.

*Encirclement retention rate:* The ability to maintain encirclement after its first establishment is quantified by

$$R_{\mathrm{enc}}=\frac{100\%}{K-k_{\mathrm{enc}}}\sum_{k=k_{\mathrm{enc}}}^{K-1}C_{k}.$$

This metric represents the percentage of sampling instants satisfying the encirclement condition from the first establishment time to the end of the simulation. It is not applicable when encirclement is not established.

*Control effort:* The squared magnitude of the applied, saturated control command $u_{i}\left(t_{k}\right)$ is integrated over the simulation:

$$J_{u}=\Delta t\sum_{k=0}^{K-2}\sum_{i=1}^{N}{∥u_{i}\left(t_{k}\right)∥}^{2}.$$

The control input in this comparison is a velocity command; therefore, $J_{u}$ has units of $m^{2}/s$ and quantifies cumulative command effort. The normalized control effort and root-mean-square command magnitude are

$${\bar{J}}_{u}=\frac{J_{u}}{NTu_{\max}^{2}},\;u_{\mathrm{RMS}}=\sqrt{\frac{J_{u}}{NT}},$$

where T=(K−1)Δt and $u_{\max}$ is the command magnitude limit. The normalized effort expresses the actual effort as a fraction of that obtained if all USVs operated at the maximum command magnitude throughout the simulation.

Control effort is additionally evaluated over 0–150s and 150–500s to examine its temporal distribution. The command saturation rate is defined as the percentage of USV–control-step combinations for which the unsaturated command exceeds the prescribed limit:

$$P_{\mathrm{sat}}=\frac{100\%}{N(K-1)}\sum_{k=0}^{K-2}\sum_{i=1}^{N}I\left[∥u_{i,\mathrm{raw}}\left(t_{k}\right)∥>u_{\max}\right].$$

#### 5.3.2. Comparison Settings

Five representative baseline implementations are considered: consensus formation control, leader–follower control, an artificial potential field method, nonlinear encirclement, and predictive encirclement. The control structures are motivated by the consensus formation framework discussed in [17], the leader–follower coordination framework discussed in [25], the potential-field hunting approach discussed in [14], the nonlinear approach mapping and angular-spacing-dependent speed modulation in [15], and the trajectory-prediction-based cooperative hunting framework in [5], respectively.

The consensus baseline combines individual reference-position errors with neighboring error differences. The leader–follower baseline assigns a target-related reference position to the leader and regulates the relative formation offsets of the followers. The artificial potential field baseline combines radial attraction toward the desired encirclement circle with angular repulsion among the USVs. The nonlinear encirclement baseline adopts the nonlinear approach mapping and angular-spacing-dependent speed modulation, supplemented with relative formation-angle regulation for the tracking-encirclement task considered here. The predictive encirclement baseline follows the trajectory-prediction, Apollonius ambush-point, U-shaped formation, and surrounding-and-shrinking mechanisms. The numerical parameters are selected for the present simulation setting, with their roles determined by the corresponding control structures.

All six methods use identical initial USV positions and target initial conditions. Specifically, the target’s evasive maneuvers and the complex marine environment configurations are kept consistent with the previous simulation setup presented in Section 5.1. The common simulation parameters are summarized in Table 4.

#### 5.3.3. Comparison of Encirclement Performance

**Figure 12:**
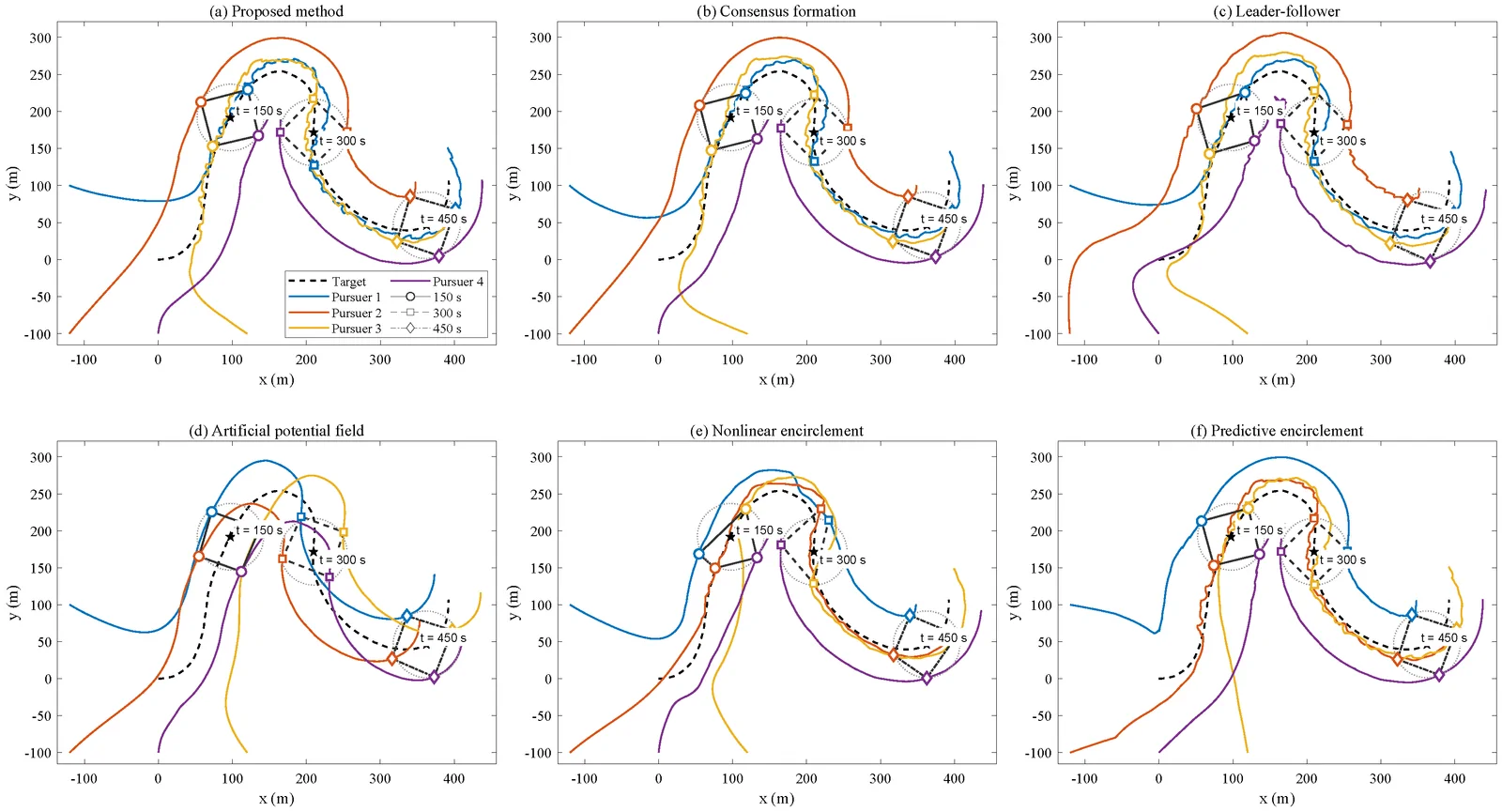
Trajectory comparison of the proposed method and five baselines, with encirclement configurations at 150s, 300s, and 450s.

Figure 12 compares the trajectories of the six methods and their encirclement configurations at 150s, 300s, and 450s. The target positions, polygons formed by the vehicles, and desired encirclement circles illustrate both geometric enclosure and radial tracking. The proposed method maintains a formation close to the desired radius as the target continues to maneuver. Predictive encirclement also maintains a stable surrounding formation after its initial approach and formation adjustment. Consensus formation, leader–follower control, and the artificial potential field method exhibit larger radial deviations during portions of the target motion. Nonlinear encirclement shows reduced inter-vessel separation and uneven spatial distribution during some intervals.

Table 5 summarizes encirclement establishment time, establishment time under the strict criterion, steady-state radial error, safety performance, and encirclement retention.

The proposed method enters its first qualifying encirclement interval at 88.9s and completes the required 10s holding interval at 98.9s, achieving the shortest establishment time among the six methods. Consensus formation, the artificial potential field method, nonlinear encirclement, and predictive encirclement establish encirclement at 110.5s, 92.3s, 119.5s, and 137.2s, respectively. Relative to these four baselines, the proposed method reduces establishment time by 19.55%, 3.68%, 25.61%, and 35.20%, respectively. Under the evaluated scenario and parameter settings, the leader–follower baseline does not simultaneously satisfy the radial tolerance and convex-hull enclosure conditions for a continuous 10s interval.

The proposed method achieves a steady-state radial root-mean-square error of 0.533m. Compared with consensus formation, leader–follower control, the artificial potential field method, and nonlinear encirclement, the reductions are 86.50%, 91.70%, 85.37%, and 64.07%, respectively. Predictive encirclement achieves a smaller steady-state radial error of 0.358m, indicating that its predicted reference supports accurate radial tracking in this scenario. The proposed method establishes encirclement earlier while maintaining the encirclement condition throughout the subsequent motion. Table 6 summarizes the relative changes.

**Figure 13:**
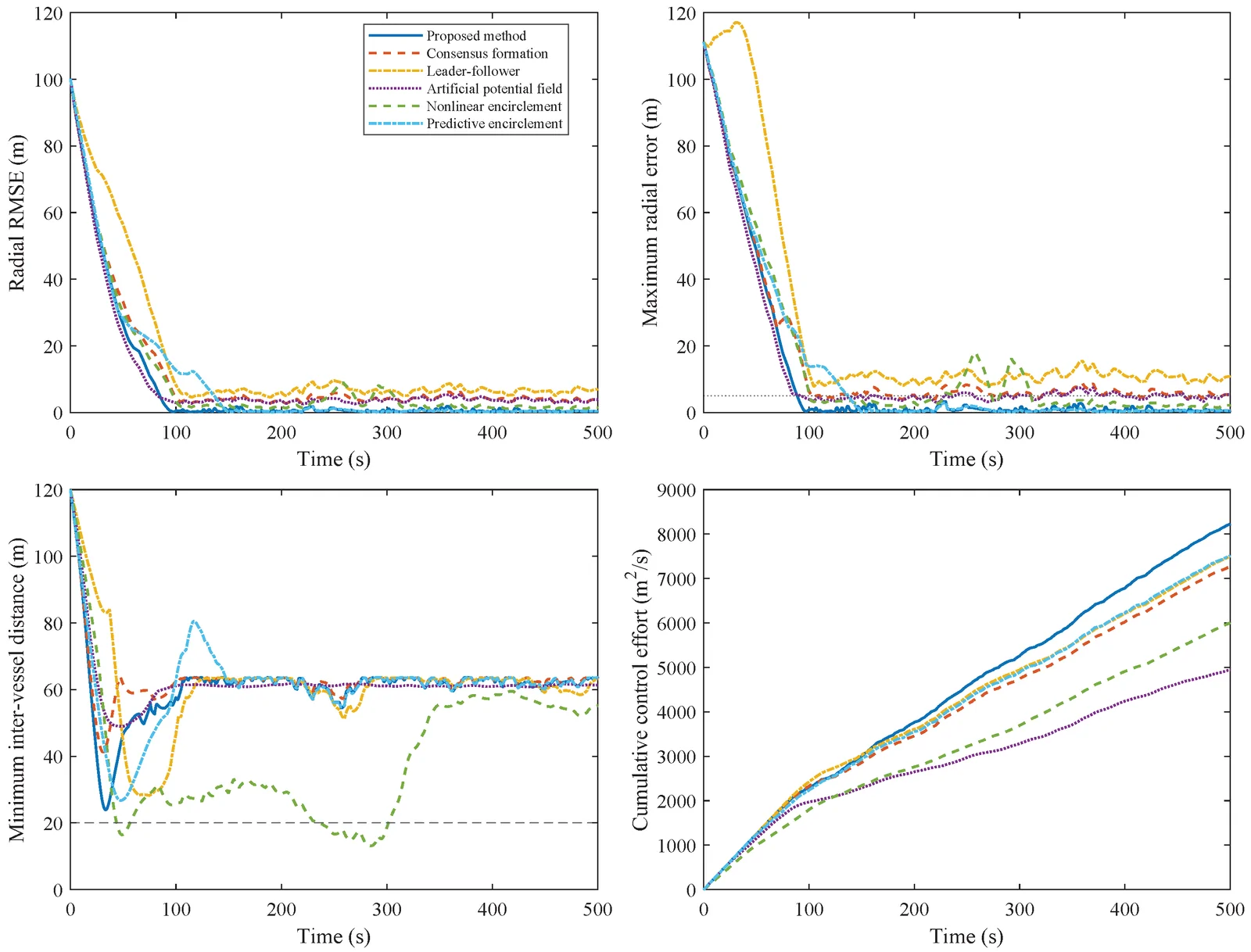
Comparison of radial root-mean-square error, maximum absolute radial error, minimum inter-vessel distance, and cumulative control effort. The black dashed horizontal line in the lower-left panel indicates the minimum safety distance of 20m.

Figure 13 shows the evolution of radial root-mean-square error, maximum absolute radial error, minimum inter-vessel distance, and cumulative control effort. The proposed method rapidly reduces radial errors during the initial approach and maintains a low error level during subsequent target maneuvers and ocean-current disturbances. Its minimum inter-vessel distance is 23.927m, providing a minimum margin of 3.927m above the prescribed 20m safety distance. The safety constraint satisfaction rate is 100%.

Consensus formation, leader–follower control, the artificial potential field method, and predictive encirclement also achieve a safety constraint satisfaction rate of 100%. In contrast, the minimum inter-vessel distance of nonlinear encirclement decreases to 13.122m, and its safety constraint satisfaction rate is 83.92%. This implementation therefore fails to maintain the prescribed separation during some intervals. These results show the importance of evaluating geometric enclosure and inter-vessel safety separately.

Encirclement retention further distinguishes the methods. Both the proposed method and predictive encirclement achieve a retention rate of 100%, maintaining the radial tolerance and geometric enclosure conditions from their first establishment times to the end of the simulation. The corresponding retention rates of consensus formation, the artificial potential field method, and nonlinear encirclement are 22.18%, 61.03%, and 80.32%, respectively. These methods establish encirclement but subsequently leave the prescribed encirclement state during portions of the remaining simulation.

**Figure 14:**
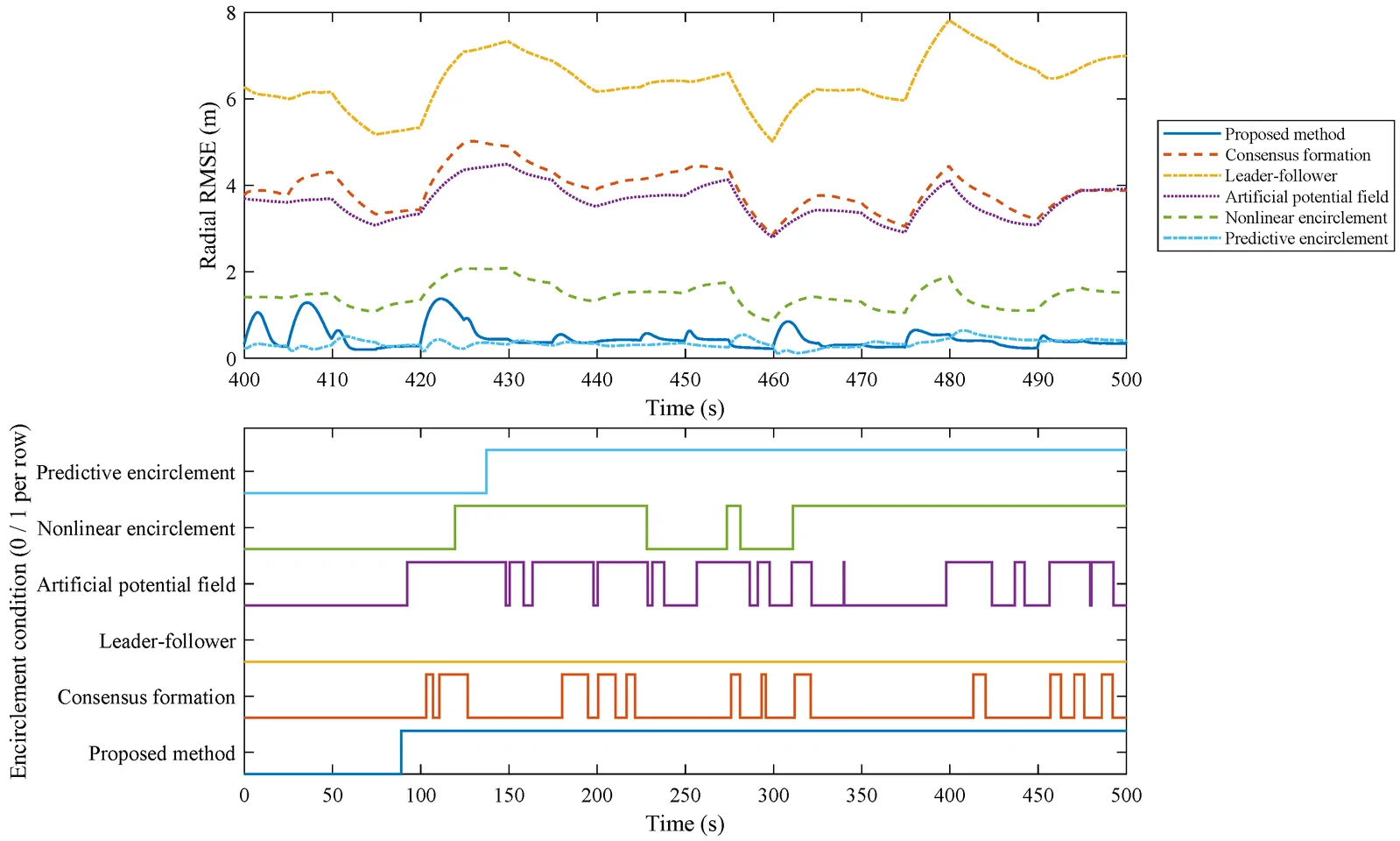
Radial root-mean-square errors over 400–500s and encirclement states over the full simulation horizon.

Figure 14 presents the radial errors over 400–500s and the encirclement states over the full simulation horizon. The proposed method and predictive encirclement maintain small steady-state radial errors and uninterrupted encirclement after establishment. When the tolerance is tightened to 2m and the holding duration is increased to 20s, the proposed method enters its first qualifying interval at 92.9s. Predictive encirclement and nonlinear encirclement meet this stricter criterion at 144.1s and 455.7s, respectively, whereas the other three baselines do not meet it during the simulation. Under the evaluated conditions, the proposed method therefore establishes and sustains an encirclement formation satisfying the prescribed accuracy requirements earlier.

#### 5.3.4. Control Effort Analysis

Table 7 reports total control effort, normalized control effort, root-mean-square command magnitude, and command saturation rate.

The proposed method has a total control effort of $8228.396\;m^{2}/s$, a root-mean-square command magnitude of 2.028m/s, and a command saturation rate of 47.54%. Its total control effort is 13.20%, 9.74%, 66.19%, 37.02%, and 9.46% higher than that of consensus formation, leader–follower control, the artificial potential field method, nonlinear encirclement, and predictive encirclement, respectively. These values indicate stronger control action in the evaluated scenario.

To examine the temporal distribution of control effort, Table 8 separates the total effort into the common intervals of 0–150s and 150–500s.

The interval-specific results show that the higher control effort of the proposed method also occurs during sustained tracking. Because the target continues to move after encirclement establishment, the vehicles must continuously adjust their positions, and cumulative control effort continues to increase. Over 150–500s, the proposed method applies $5230.420\;m^{2}/s$ of control effort, exceeding that of the five baselines and reflecting its sustained use of stronger control commands.

The combined performance and effort results quantify the associated trade-offs. Relative to consensus formation, the proposed method increases control effort by 13.20% while reducing steady-state radial error by 86.50% and improving encirclement retention from 22.18% to 100%. Relative to nonlinear encirclement, it increases control effort by 37.02% while reducing establishment time by 25.61%, improving retention from 80.32% to 100%, and increasing safety constraint satisfaction from 83.92% to 100%. The artificial potential field method requires the lowest control effort but exhibits a larger steady-state radial error and an encirclement retention rate of 61.03%.

Predictive encirclement achieves both a safety constraint satisfaction rate and an encirclement retention rate of 100%, together with a smaller steady-state radial error and lower control effort. Compared with this baseline, the proposed method uses 9.46% more control effort while reducing establishment time from 137.2s to 88.9s, establishing sustained encirclement 48.3s earlier. It also reduces establishment time under the strict criterion from 144.1s to 92.9s. These results characterize the differences among the evaluated implementations in establishment speed, steady-state accuracy, and control effort. In the tested scenario, the proposed method establishes encirclement rapidly and subsequently maintains the prescribed encirclement and inter-vessel safety requirements throughout the remaining target motion.

#### 5.3.5. Comparative Evaluation Under Varying Conditions

To examine performance beyond a single scenario, additional comparative simulations are conducted by varying the number of pursuit USVs, the target-to-pursuer maximum speed ratio, the communication delay, and the packet-loss probability. The proposed method is compared with the five baseline implementations described in the preceding comparison settings: consensus formation, leader–follower control, artificial potential fields, nonlinear encirclement, and predictive encirclement. Within each trial, all methods share the same initial positions, target trajectory, ocean-current disturbances, communication events, speed limits, and evaluation criteria.

(1) **Simulation settings and statistical evaluation**

The reference condition uses four pursuit USVs, a target-to-pursuer maximum speed ratio of 0.6, zero communication delay, and no packet loss. Four groups of comparisons are constructed by independently varying the number of pursuit vessels over {4,6,8}, the maximum speed ratio over {0.4,0.6,0.8}, the communication delay over {0,0.2,0.5}s, and the packet-loss probability over {0,10,30}%. The same reference condition is reused in these four groups, yielding nine distinct conditions. Each condition is evaluated through 20 randomized trials for each of the six methods, resulting in 1080 simulation runs.

The desired encirclement radius is fixed at 45m for all swarm sizes, and the minimum inter-vessel safety distance is 20m. The maximum control speed of each pursuit USV is 2.5m/s. Each simulation lasts 500s with an integration step of 0.1s. The pursuit USVs initially occupy an arc on one side of the target, with nominal bearings ranging from 100° to 260° and a nominal distance of 150m from the target. Random perturbations include radial offsets within ±5m, a common angular offset within ±5°, and individual angular offsets within ±2°. Paired random seeds are used across methods and conditions. Within each trial, the target speed profile is scaled to attain the prescribed maximum speed ratio, while its heading profile and the ocean-current realization are retained.

In the communication tests, target position and heading information and individual USV positions are broadcast every 0.1s. Communication impairment is implemented using a fixed delivery delay or independent Bernoulli packet losses across sources and transmission times. A lost broadcast packet is unavailable to all receivers, and the most recently received information is retained until a new packet arrives. Local positioning and inter-USV relative ranging within 40m remain available. The proposed method’s kinematic safety layer jointly enforces inter-USV safety and individual speed constraints through a centralized computation, with an internal safety buffer of 0.02m. This safety coordination is not subjected to the broadcast impairments. The communication tests therefore evaluate the effects of delayed or missing target and formation information under an available local safety-sensing and coordination channel.

The encirclement criterion is consistent with the preceding comparisons: all radial errors must remain within 5m, the target must lie strictly inside the convex hull of the pursuit USVs, and these conditions must persist for at least 10s. The establishment time is the beginning of the first qualifying interval. Steady-state radial root-mean-square error is evaluated over the final 100s. Control effort is calculated from the actual applied velocity commands over the complete simulation interval, and its normalization is explained below.

**Figure 15:**
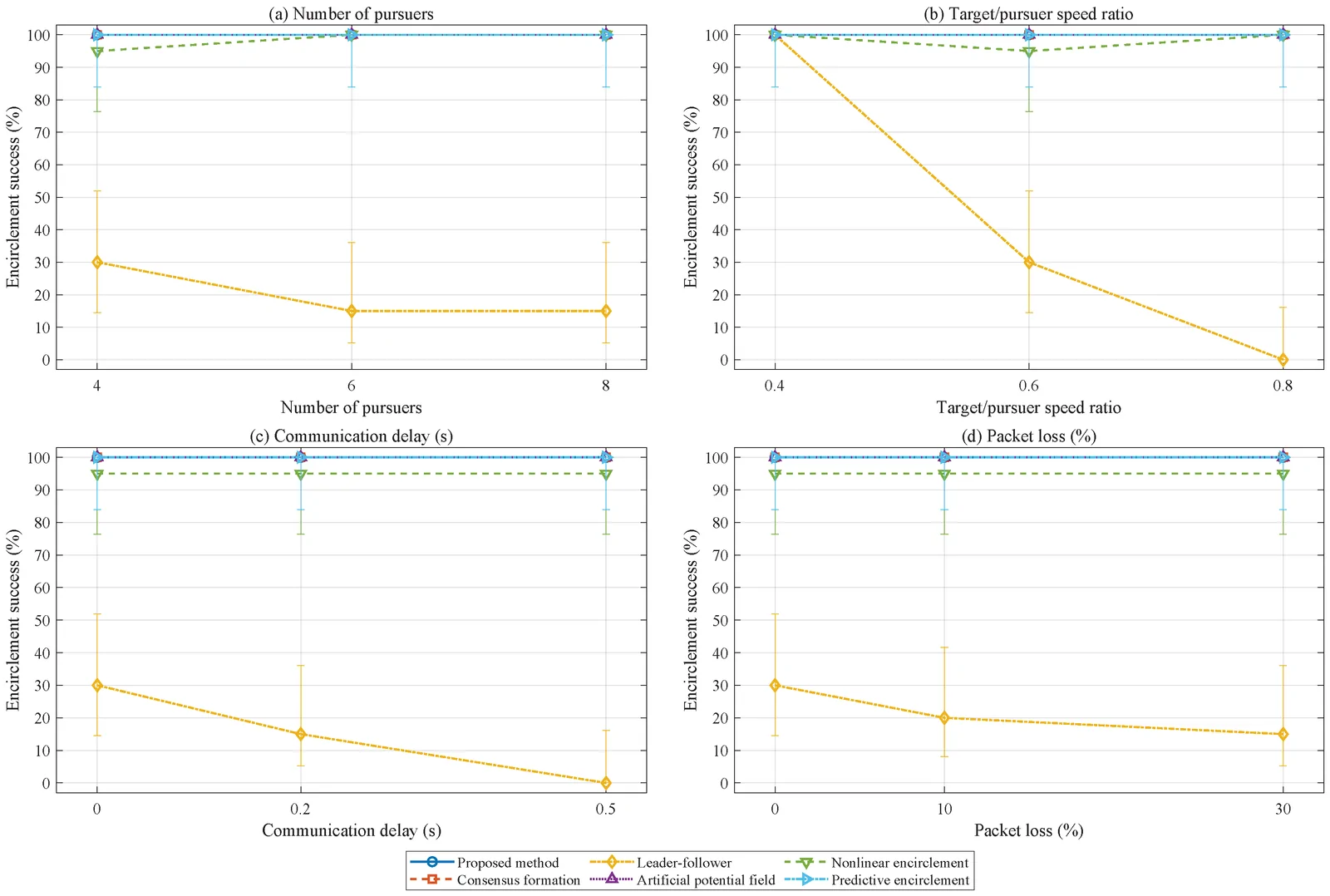
Encirclement success rates under different operating conditions: (**a**) number of pursuit USVs; (**b**) target-to-pursuer maximum speed ratio; (**c**) communication delay; and (**d**) packet-loss probability. Error bars represent 95% Wilson confidence intervals based on 20 trials per condition.

**Figure 16:**
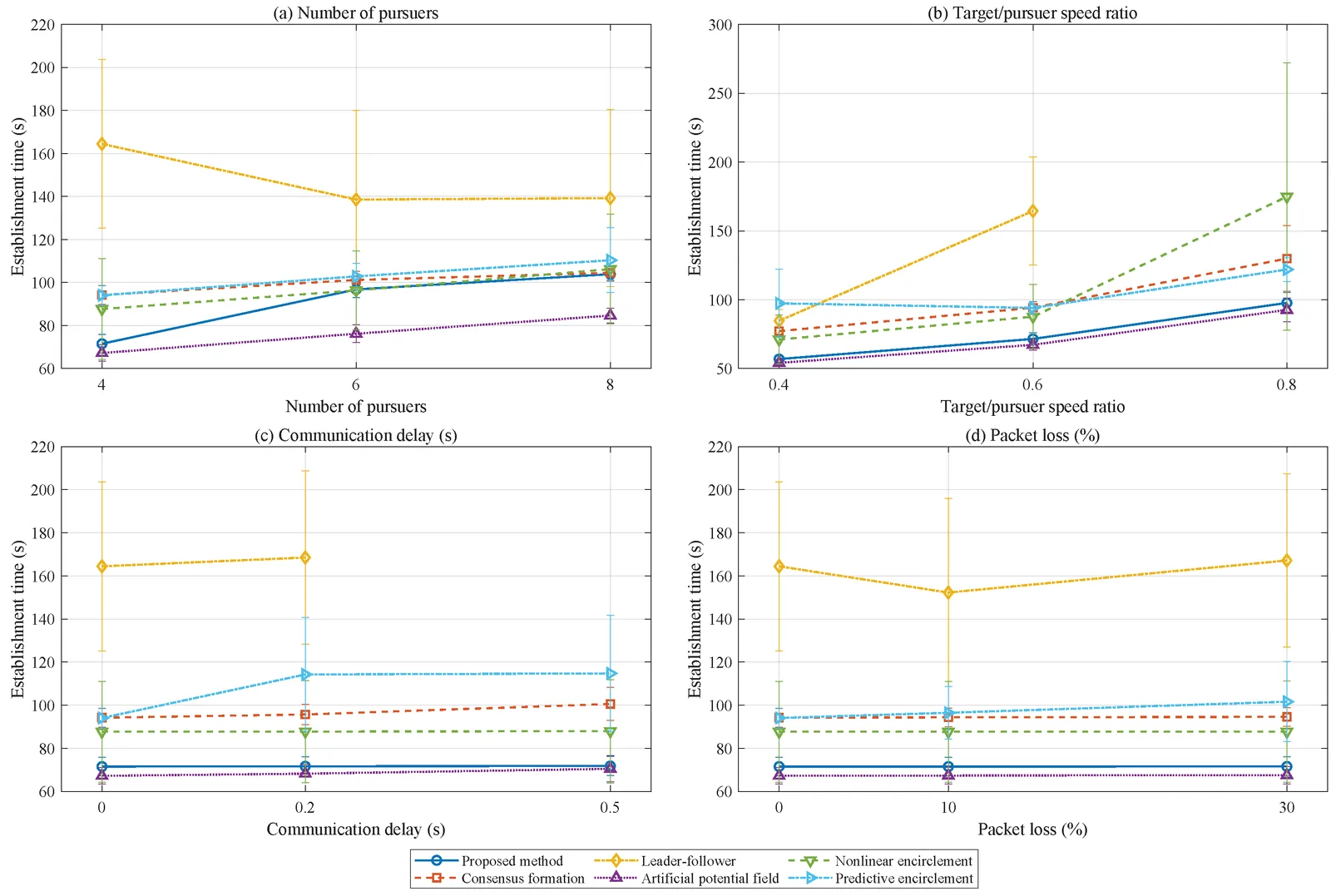
Encirclement establishment times under different operating conditions: (**a**) number of pursuit vessels; (**b**) target-to-pursuer maximum speed ratio; (**c**) communication delay; and (**d**) packet-loss probability. Data points and error bars represent means and sample standard deviations over successful trials. No establishment-time value is reported when all trials fail to establish encirclement.

**Figure 17:**
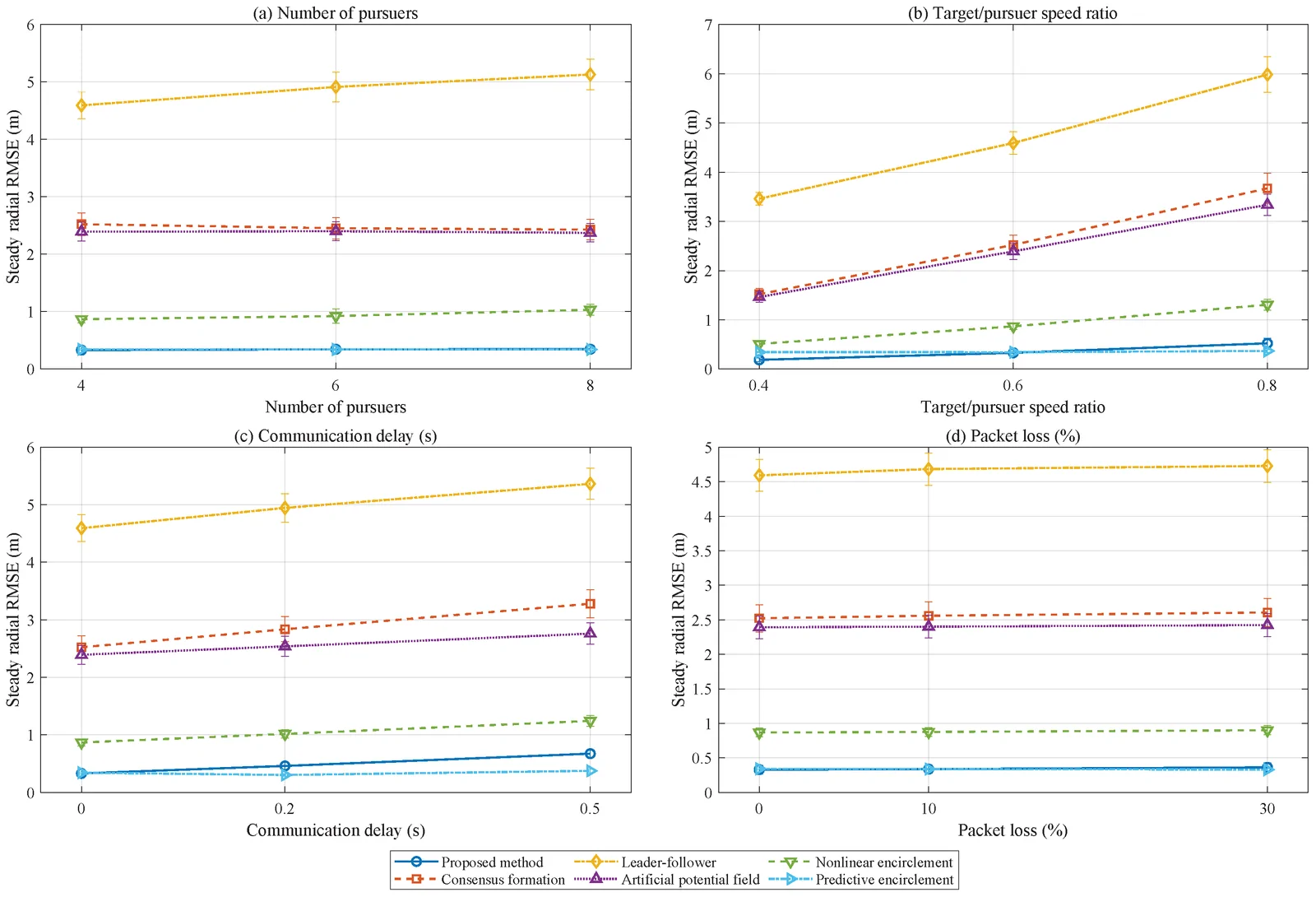
Steady-state radial root-mean-square errors under different operating conditions: (**a**) number of pursuit USVs; (**b**) target-to-pursuer maximum speed ratio; (**c**) communication delay; and (**d**) packet-loss probability. Data points and error bars represent means and sample standard deviations across all 20 trials.

**Figure 18:**
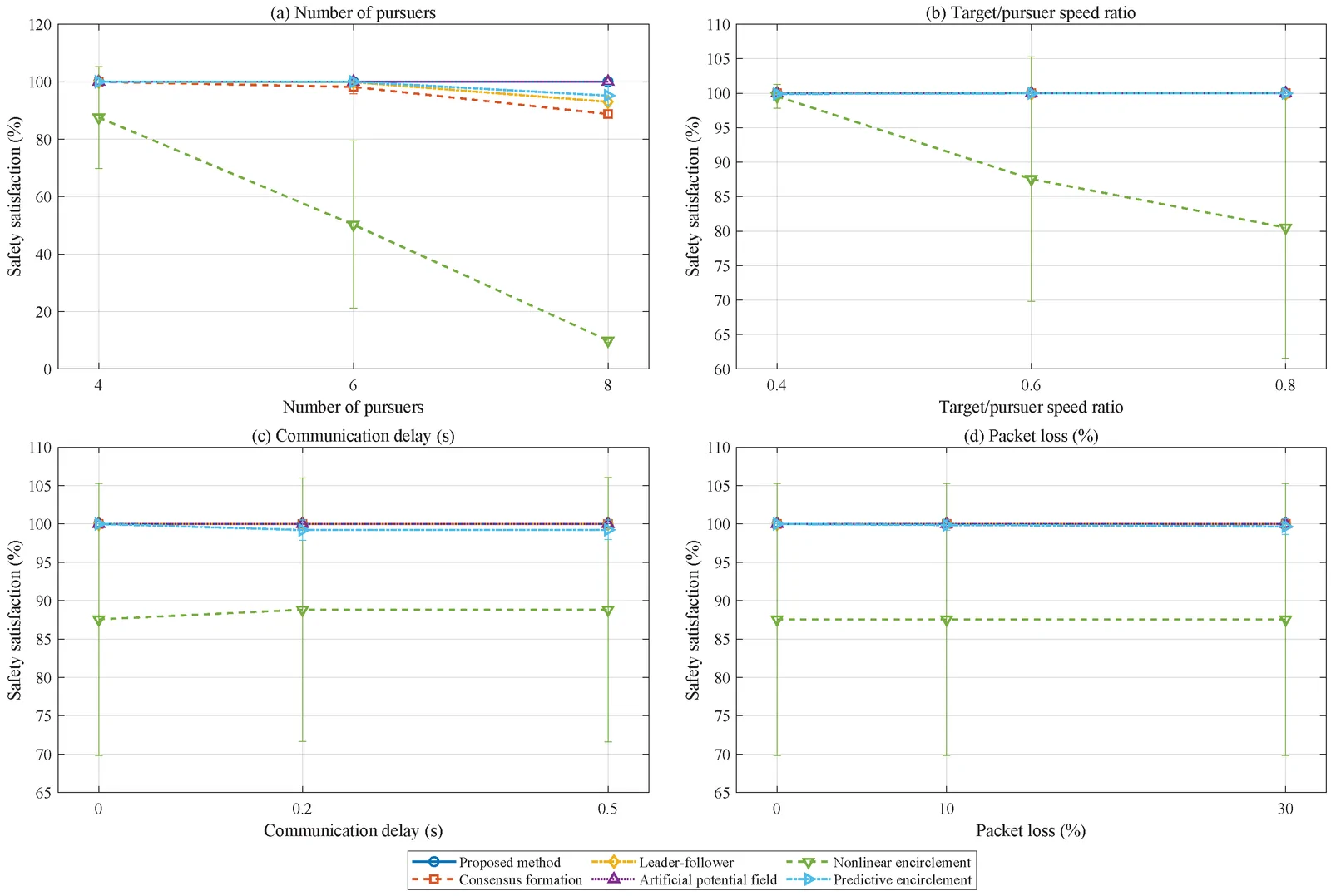
Sampled safety-constraint satisfaction rates under different operating conditions: (**a**) number of pursuit USVs; (**b**) target-to-pursuer maximum speed ratio; (**c**) communication delay; and (**d**) packet-loss probability. Data points and error bars represent means and sample standard deviations across all 20 trials.

**Figure 19:**
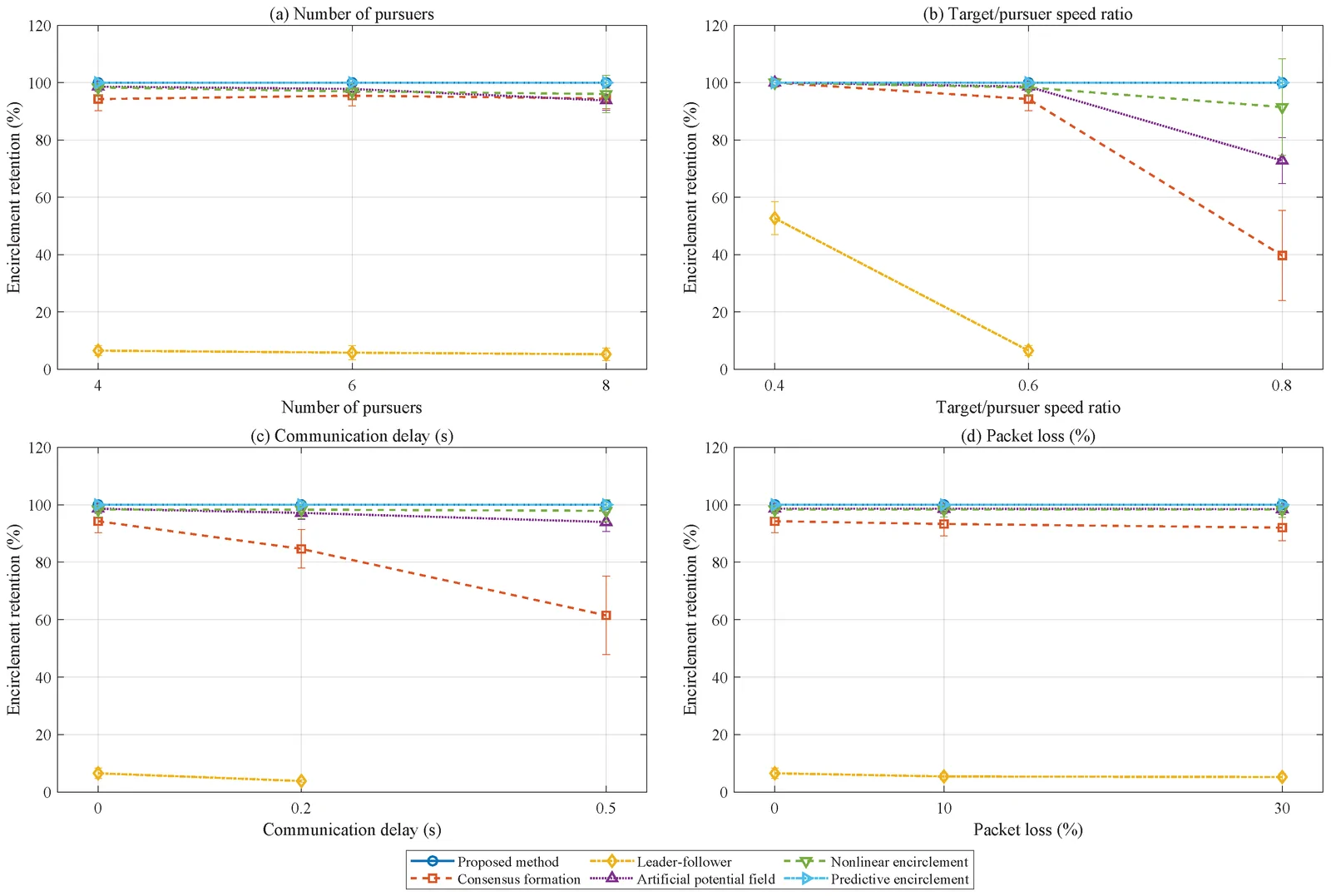
Encirclement retention rates under different operating conditions: (**a**) number of pursuit USVs; (**b**) target-to-pursuer maximum speed ratio; (**c**) communication delay; and (**d**) packet-loss probability. Data points and error bars represent means and sample standard deviations over trials that establish encirclement. No retention value is reported when all trials fail to establish encirclement.

**Figure 20:**
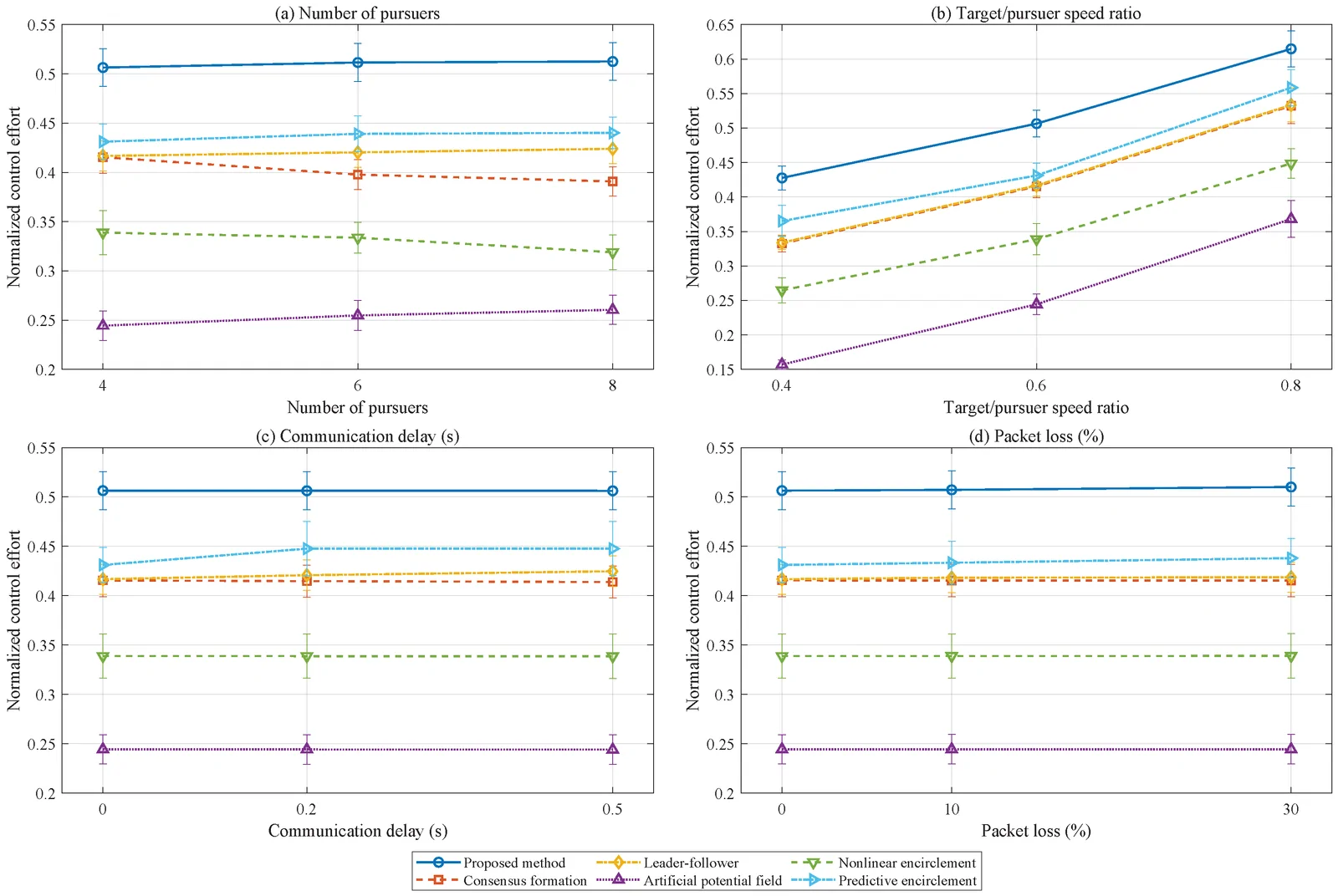
Normalized control effort under different operating conditions: (**a**) number of pursuit vessels; (**b**) target-to-pursuer maximum speed ratio; (**c**) communication delay; and (**d**) packet-loss probability. Data points and error bars represent means and sample standard deviations across all 20 trials.

Figure 15 reports the encirclement success rates with 95% Wilson confidence intervals. Figure 16, Figure 17, Figure 18, Figure 19 and Figure 20 report means and sample standard deviations. Establishment time and retention statistics include only trials in which encirclement is established; unsuccessful trials are not assigned artificial establishment times. Error, safety, and control-effort statistics include all trials. Safety satisfaction measures the percentage of sampled times at which all inter-vessel distances satisfy the 20m requirement. In addition, the minimum separation within each integration interval is evaluated analytically from the relative motion segments.

Table 9 summarizes the proposed method’s performance. All 20 trials under each condition achieve encirclement, with 100% retention and sampled safety satisfaction. The inter-sample checks also identify no safety violations in these trials.

(2) **Effect of swarm size**

Panels (a) of Figure 15, Figure 16, Figure 17, Figure 18, Figure 19 and Figure 20 show the effects of increasing the number of pursuit USVs. As the swarm size increases from four to eight, the proposed method’s mean establishment time increases from 71.52 to 103.88s, while its steady-state radial root-mean-square error changes only from 0.330 to 0.345m. Encirclement success and retention remain at 100% for all three swarm sizes, indicating that accurate and sustained encirclement is maintained within the tested range.

With the encirclement radius fixed at 45m, increasing the number of USVs reduces the desired neighboring separation and introduces more interactions during formation establishment. The mean activation rates of the proposed method’s safety correction are 0.525% and 9.981% in the six- and eight-USV cases, respectively. The corresponding worst-case inter-sample minimum distances are 20.0215 and 20.0200m, satisfying the 20m safety requirement. These results show that the safety layer intervenes when USVs approach one another while allowing the formation-tracking task to proceed.

Table 10 provides a detailed comparison for the eight-USV condition. The proposed method achieves a mean establishment time of 103.88s, which is 5.86% shorter than that of predictive encirclement. Their radial errors are similar, whereas their proportions of trials satisfying the safety requirement throughout the complete simulation are 100% and 30%, respectively. Compared with the artificial potential field method, the proposed method reduces radial root-mean-square error by 85.45% and increases retention from 93.81% to 100%. The artificial potential field method establishes encirclement more quickly. These comparisons identify differences in establishment speed, formation accuracy, and sustained safe encirclement.

(3) **Effect of the target-to-pursuer speed ratio**

Panels (b) of Figure 15, Figure 16, Figure 17, Figure 18, Figure 19 and Figure 20 show that increasing the target speed raises the difficulty of establishing and maintaining encirclement. As the maximum speed ratio increases from 0.4 to 0.8, the proposed method’s mean establishment time increases from 56.88 to 97.68s, and its radial root-mean-square error increases from 0.187 to 0.523m. Nevertheless, encirclement success, retention, and safety satisfaction remain at 100% under all three tested speed ratios.

At a speed ratio of 0.8, consensus formation and artificial potential fields still establish encirclement, but their mean retention rates decrease to 39.68% and 72.82%, respectively. The leader–follower method fails to establish encirclement in all 20 trials. Both the proposed method and predictive encirclement retain encirclement throughout the post-establishment period. The proposed method’s mean establishment time is 19.91% shorter than the 121.96s achieved by predictive encirclement. Predictive encirclement produces a smaller radial error of 0.368m, compared with 0.523m for the proposed method. Thus, the proposed method provides faster establishment while maintaining sustained encirclement and inter-vessel safety under the tested higher-speed condition.

(4) **Effects of communication delay and packet loss**

Panels (c) of Figure 15, Figure 16, Figure 17, Figure 18, Figure 19 and Figure 20 illustrate the effects of fixed communication delay. Increasing the delay from 0 to 0.5s changes the proposed method’s mean establishment time from 71.52 to 71.80s, an increase of only 0.28s. Its radial root-mean-square error increases from 0.330 to 0.674m, reflecting the tracking mismatch associated with delayed target information. Despite this increase, encirclement success, retention, and safety satisfaction remain at 100%.

At a delay of 0.5s, consensus formation yields a radial root-mean-square error of 3.279m and a retention rate of 61.49%, while the leader–follower method fails to establish encirclement. Predictive encirclement maintains a lower radial error but requires a mean establishment time of 114.73s. The proposed method reduces this time by 37.42%, indicating faster encirclement establishment together with sustained formation retention under the tested delay.

Panel (d) shows that independent random packet losses have a smaller effect on the proposed method. As the packet-loss probability increases from 0% to 30%, its mean establishment time changes from 71.52 to 71.55s, and its radial root-mean-square error increases from 0.330 to 0.363m. Encirclement success, retention, and safety satisfaction remain at 100%. With the 0.1s broadcast interval and retention of the latest received packet, the mean age of target information is approximately 0.043s at 30% packet loss. This is substantially shorter than the 0.5s fixed-delay condition and helps explain the smaller performance change observed in the packet-loss tests.

(5) **Control effort analysis**

To facilitate comparisons across different swarm sizes, Figure 20 presents the normalized control effort. This quantity is calculated from the control effort defined in the preceding experiments as

$${\bar{J}}_{u}=\frac{J_{u}}{NTv_{\max}^{2}},\;J_{u}=\int_{0}^{T}\sum_{i=1}^{N}{∥u_{i}\left(t\right)∥}^{2}\;dt,$$

where *N* is the number of pursuit USVs, *T* is the simulation duration, $v_{\max}$ is the maximum admissible control speed, and $u_{i}\left(t\right)$ is the control velocity actually applied to USV *i*. The original control effort $J_{u}$ has units of $m^{2}/s$, whereas ${\bar{J}}_{u}$ is dimensionless. It represents the squared control speed, averaged over time and vessels, relative to the squared speed limit. Here, T=500s and $v_{\max}=2.5\;m/s$. The calculation therefore uses the same underlying control-effort measure as the preceding experiments, with normalization introduced to account for differences in swarm size.

As shown in Figure 20a, increasing the number of pursuit USVs from four to eight raises the proposed method’s mean normalized control effort from 0.5064 to 0.5125, an increase of approximately 1.21%. The corresponding mean total control efforts are 6330.08 and 12813.54 $m^{2}/s$, respectively. This indicates that the increase in total control effort is mainly associated with the larger number of vessels, while average control expenditure per vessel changes only slightly.

Figure 20b shows that increasing the maximum speed ratio from 0.4 to 0.8 raises the proposed method’s mean normalized control effort from 0.4277 to 0.6148. This trend reflects the greater control demand associated with tracking a faster target. As illustrated in Figure 20c, the mean normalized control effort remains approximately 0.506 as the communication delay increases from 0 to 0.5s. Figure 20d shows that increasing the packet-loss probability from 0% to 30% raises this quantity from 0.5064 to 0.5101, an increase of approximately 0.72%. Together with the tracking-error and retention results, these observations indicate relatively stable control expenditure under the tested communication conditions.

The proposed method requires greater control effort than some baseline methods. In the eight-vessel case, the mean normalized control efforts of the proposed method and the artificial potential field method are 0.5125 and 0.2605, respectively. Their corresponding radial root-mean-square errors are 0.345 and 2.372m, and their retention rates are 100% and 93.81%. These results characterize a trade-off between control expenditure and encirclement performance: the proposed method combines greater control expenditure with more accurate and sustained encirclement while satisfying the inter-vessel safety requirement.

### 5.4. Ablation Study with an Actively Evading Target

#### 5.4.1. Experimental Settings

An ablation study is conducted to examine the contributions of game-based decision-making, fixed-time control, and CBF-based safety control. The full implementation is compared with three variants, each removing or replacing one component while retaining the parameters of the remaining components. The experimental configurations are summarized in Table 11.

The initial USV positions, desired encirclement radius, safety distance, command magnitude limit, and ocean-current parameters are inherited from the baseline comparison. The simulation duration is 500s, and the sampling interval is 0.1s. The desired encirclement radius and safety distance are 45m and 20m, respectively, while the USV command magnitude limit is 2.5m/s. In the variant without fixed-time control, the linear feedback gain is selected to match the feedback command magnitude of the fixed-time control law at a componentwise error of 1m.

To evaluate closed-loop encirclement under adversarial conditions, the target actively adjusts its heading and speed according to the current pursuer positions and their motion history. Its escape policy considers separation from the pursuers and its own motion effort, subject to speed, acceleration, and turning-rate limits. The target speed ranges from 1.0 to 1.8m/s, with a maximum acceleration of $0.15\;m/s^{2}$ and a maximum turning rate of 0.12rad/s. All configurations share the same target escape policy, initial states, and ocean-current sequence. Because the target responds to the encirclement situation, its realized trajectory is generated separately by each closed-loop interaction.

The evaluation metrics and calculation procedures are consistent with those used in the baseline comparison, including encirclement establishment time, steady-state radial RMSE, steady-state position RMSE, minimum inter-vessel distance, safety satisfaction rate, encirclement retention rate, and control effort. The primary establishment criterion uses a radial tolerance of 5m and a holding duration of 10s. A stricter criterion uses a tolerance of 2m and a holding duration of 20s. Steady-state errors are evaluated over 400–500s.

#### 5.4.2. Encirclement Performance

**Figure 21:**
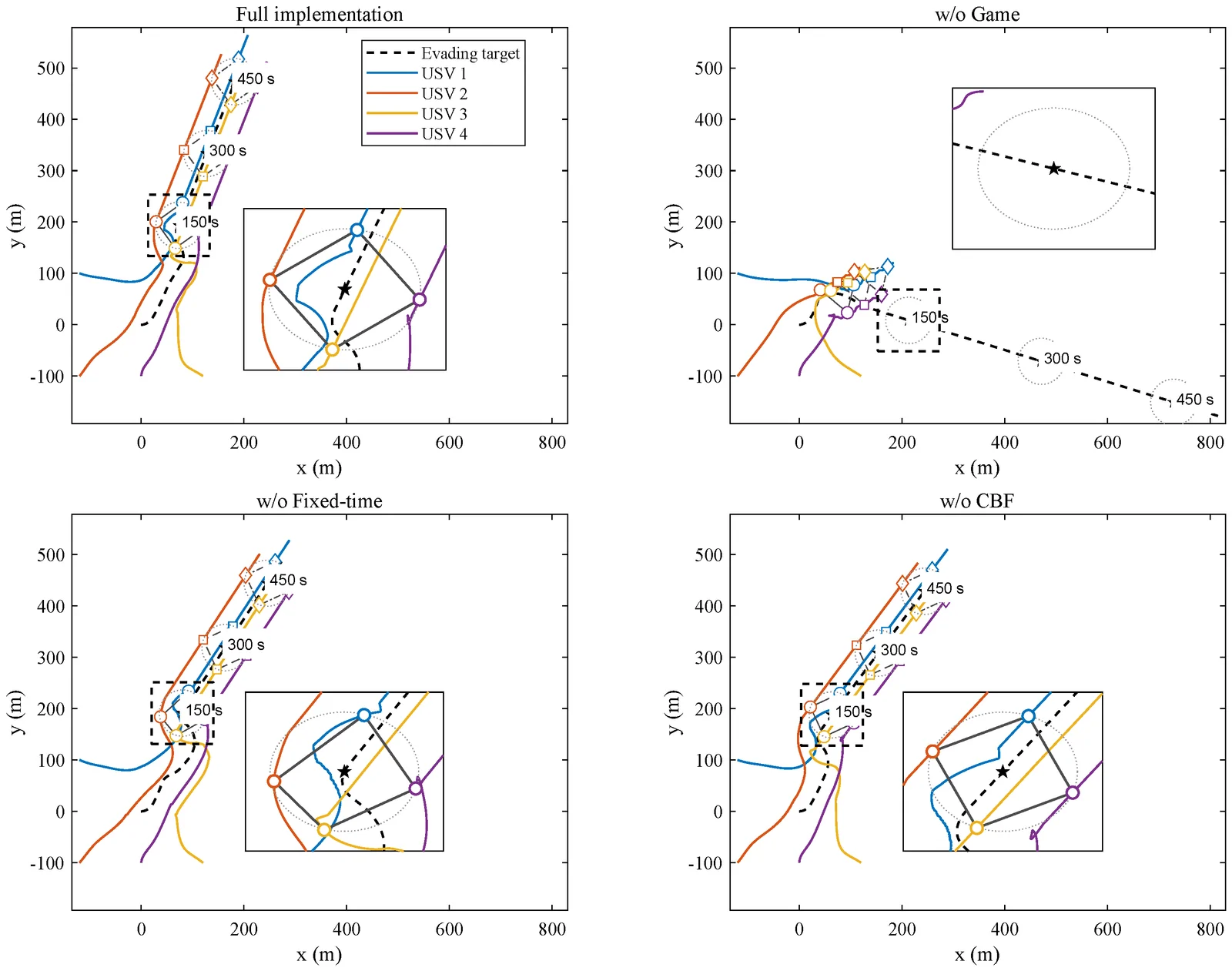
Trajectories and encirclement configurations at 150s, 300s, and 450s for the ablation study with an actively evading target. Black stars denote the target positions. Colored circles, squares, and diamonds denote the USV positions at 150s, 300s, and 450s, respectively, with colors matching the corresponding trajectories.

Figure 21 presents the trajectories and representative encirclement configurations. The quantitative results are summarized in Table 12. The full implementation and the variants without fixed-time control and without CBF establish encirclement and maintain the prescribed encirclement condition until the end of the simulation. The variant without game-based decision-making does not achieve the establishment criterion in this run.

The full implementation enters its first qualifying encirclement interval at 113.3s and completes the required 10s holding interval at 123.3s. Its steady-state radial and position RMSEs are 0.205m and 0.290m, respectively, and its encirclement retention rate is 100%. These results show that the implementation maintains the prescribed encirclement relationship while the target actively adjusts its motion. With the radial tolerance tightened to 2m and the holding duration increased to 20s, the full implementation enters a qualifying interval at 131.7s.

Replacing fixed-time control with linear feedback increases the establishment time to 126.5s and the strict establishment time to 148.6s. Relative to this variant, the full implementation reduces these times by 10.43% and 11.37%, respectively. It also reduces the steady-state radial RMSE from 0.273m to 0.205m, corresponding to approximately 24.89%, calculated from the unrounded values. The steady-state position RMSE decreases from 0.386m to 0.290m. These results indicate improved response speed and tracking accuracy with fixed-time control in the evaluated closed-loop experiment.

The variant without game-based decision-making does not establish encirclement, with steady-state radial and position RMSEs of 594.690m and 639.920m, respectively.

#### 5.4.3. Safety Constraints and Control Effort

**Figure 22:**
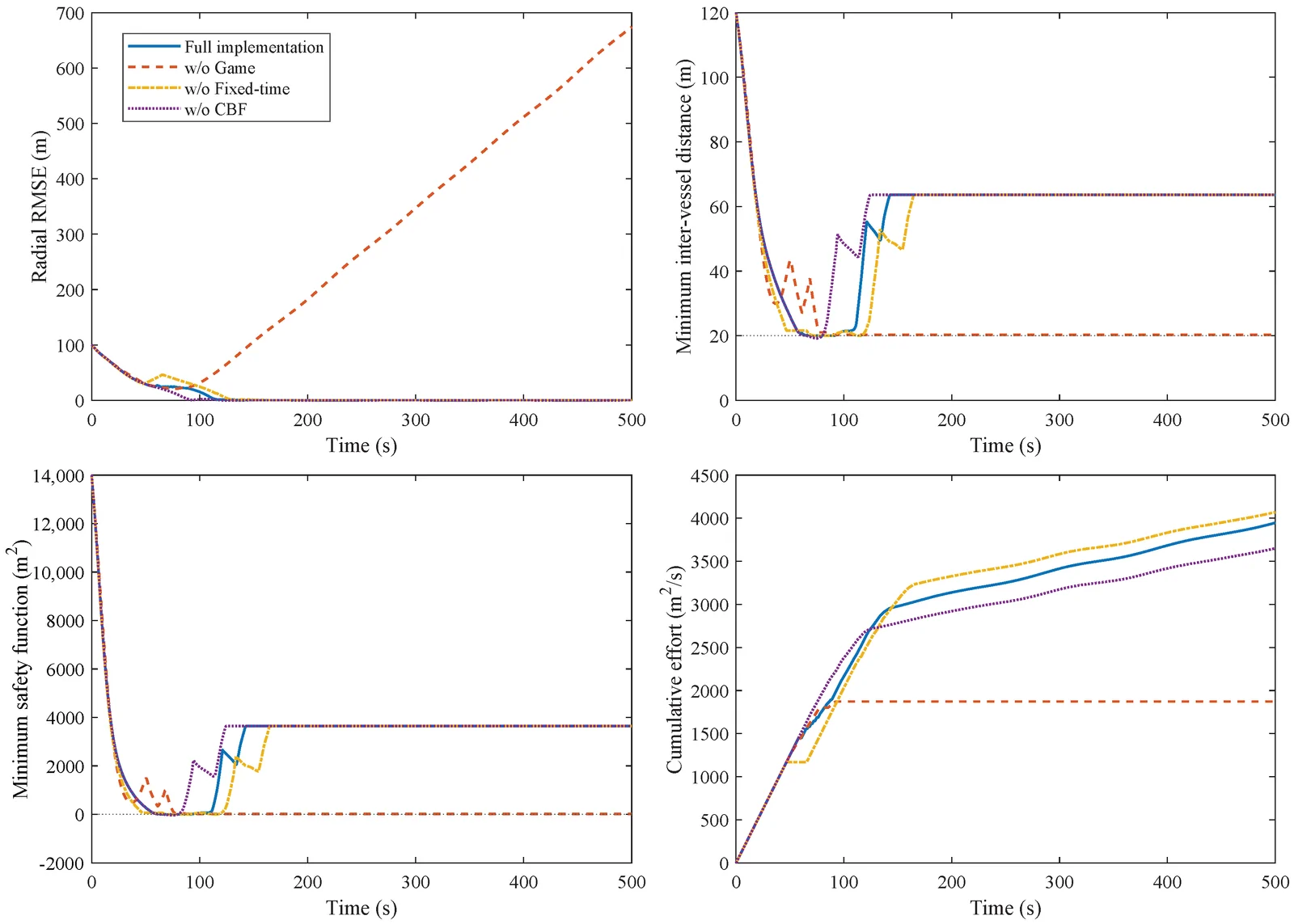
Comparison of encirclement errors, inter-vessel distances, and cumulative control effort for the ablation configurations. The black horizontal reference lines indicate the minimum safety distance of 20m in the upper-right panel and the zero safety-function threshold in the lower-left panel.

Figure 22 presents the encirclement errors, inter-vessel distances, and cumulative control effort. The control-effort statistics are reported in Table 13.

The full implementation has a control effort of $3946.5\;m^{2}/s$, approximately 3.04% lower than that of the variant without fixed-time control. Together with the establishment times and steady-state errors, this result shows that the full implementation achieves faster encirclement and more accurate tracking with lower cumulative control effort in this run. Although the variant without game-based decision-making applies less control effort, it does not complete the encirclement task; its lower effort therefore does not indicate higher task efficiency.

Removing CBF-based safety control reduces the establishment time to 88.9s and the control effort to $3649.9\;m^{2}/s$. However, the minimum inter-vessel distance decreases to 19.203m, below the prescribed 20m safety distance. Its minimum safety-function value is $-31.233\;m^{2}$, and its safety satisfaction rate decreases to 97.141%. Thus, the proximity penalty in formation planning and the nominal controller alone do not consistently maintain the required inter-vessel separation.

The full implementation maintains a minimum inter-vessel distance of 20.001m and achieves a safety satisfaction rate of 100%. Relative to the variant without CBF, its control effort increases by approximately 8.13%, and its establishment time increases by 24.4s. These differences characterize the control and time requirements associated with satisfying the safety constraints. The full implementation simultaneously achieves the prescribed radial accuracy, sustained encirclement, and inter-vessel safety at the sampled instants.

**Figure 23:**
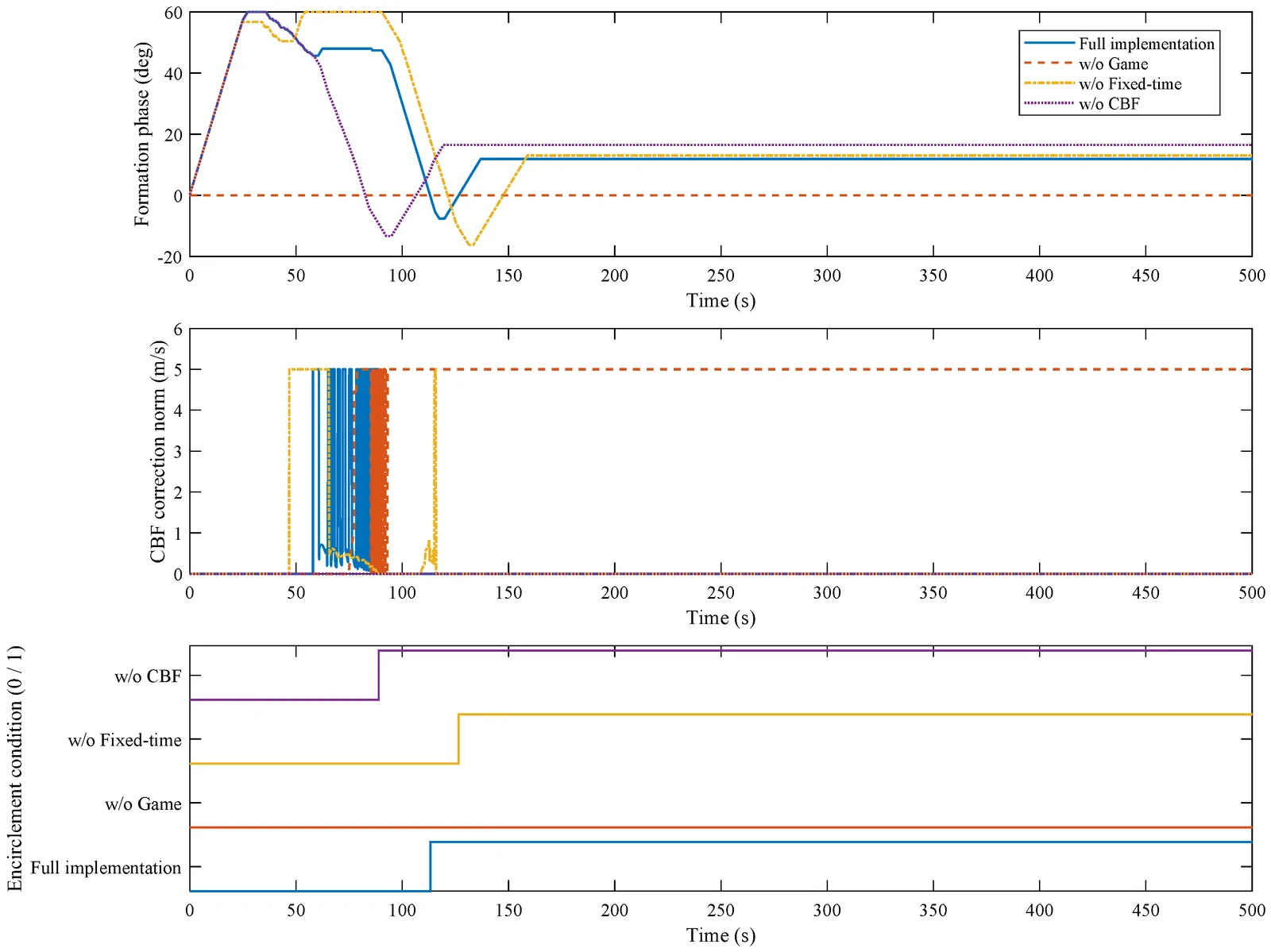
Formation decision adjustments, safety-control intervention, and encirclement states during the ablation experiments.

Encirclement retention and safety satisfaction describe different task requirements. Although the variant without CBF achieves an encirclement retention rate of 100%, the retention criterion includes only the radial tolerance and geometric enclosure of the target. It does not include the inter-vessel safety-distance requirement. Consequently, both metrics are needed to assess the quality of task completion. Figure 23 illustrates the formation decision adjustments, safety-control intervention, and encirclement states during the ablation experiments.

The ablation results demonstrate that the full implementation combines encirclement accuracy, sustained enclosure, and safety-constraint satisfaction under active target evasion. The configuration with fixed-time control exhibits faster establishment, smaller steady-state errors, and lower control effort, while retaining CBF-based safety control satisfies the distance requirement violated by the configuration without CBF.

### 5.5. Monte Carlo Validation of Tracking Encirclement

#### 5.5.1. Simulation Settings and Statistical Results

To evaluate the reliability of tracking encirclement under variations in initial conditions and random disturbances, 100 Monte Carlo trials were conducted. The target motion, encirclement reference configuration, and ocean-current settings followed the first simulation example without comparative methods, while the controller parameters and simulation duration remained unchanged. Each coordinate of the initial position of each USV was independently perturbed within ±20m of its nominal value, with the initial inter-vessel distances constrained to be at least 20m. The initial target heading and speed were uniformly sampled from [−π/6,π/6]rad and [1.2,1.6]m/s, respectively. Different random seeds were used to generate target-motion perturbations and random ocean-current disturbances. This experiment employed reference positions rotating with the target heading and evaluated the tracking-encirclement performance of fixed-time control combined with CBF safety filtering.

The performance metrics and calculation procedures were consistent with those used in the preceding comparative experiments. The standard encirclement criterion required the absolute radial error of every USV to remain within 5m and the target to remain strictly inside the convex hull formed by the USVs for at least 10s. The beginning of the first qualifying interval was defined as the encirclement establishment time, while its confirmation time occurred after the required holding interval. The strict criterion reduced the radial-error tolerance to 2m and extended the holding interval to 20s. Steady-state radial and position RMSEs were evaluated over 400–500s. Safety satisfaction and control effort were evaluated over the entire simulation, while encirclement retention was calculated using the standard criterion after establishment. Continuous metrics were summarized using their means, standard deviations, and ranges across the 100 trials. A 95% Wilson confidence interval was additionally reported for the encirclement success rate.

As shown in Table 14, all 100 trials successfully established encirclement under both the standard and strict criteria, yielding success rates of 100% and a corresponding 95% Wilson confidence interval of [96.30%,100%]. After establishment, every trial continuously satisfied the standard encirclement criterion, resulting in 100% encirclement retention. All trials also achieved 100% safety satisfaction throughout the simulation. These results demonstrate reliable encirclement establishment, sustained containment of the target, and safe inter-vessel separation within the tested ranges of initial conditions and random disturbances.

**Figure 24:**
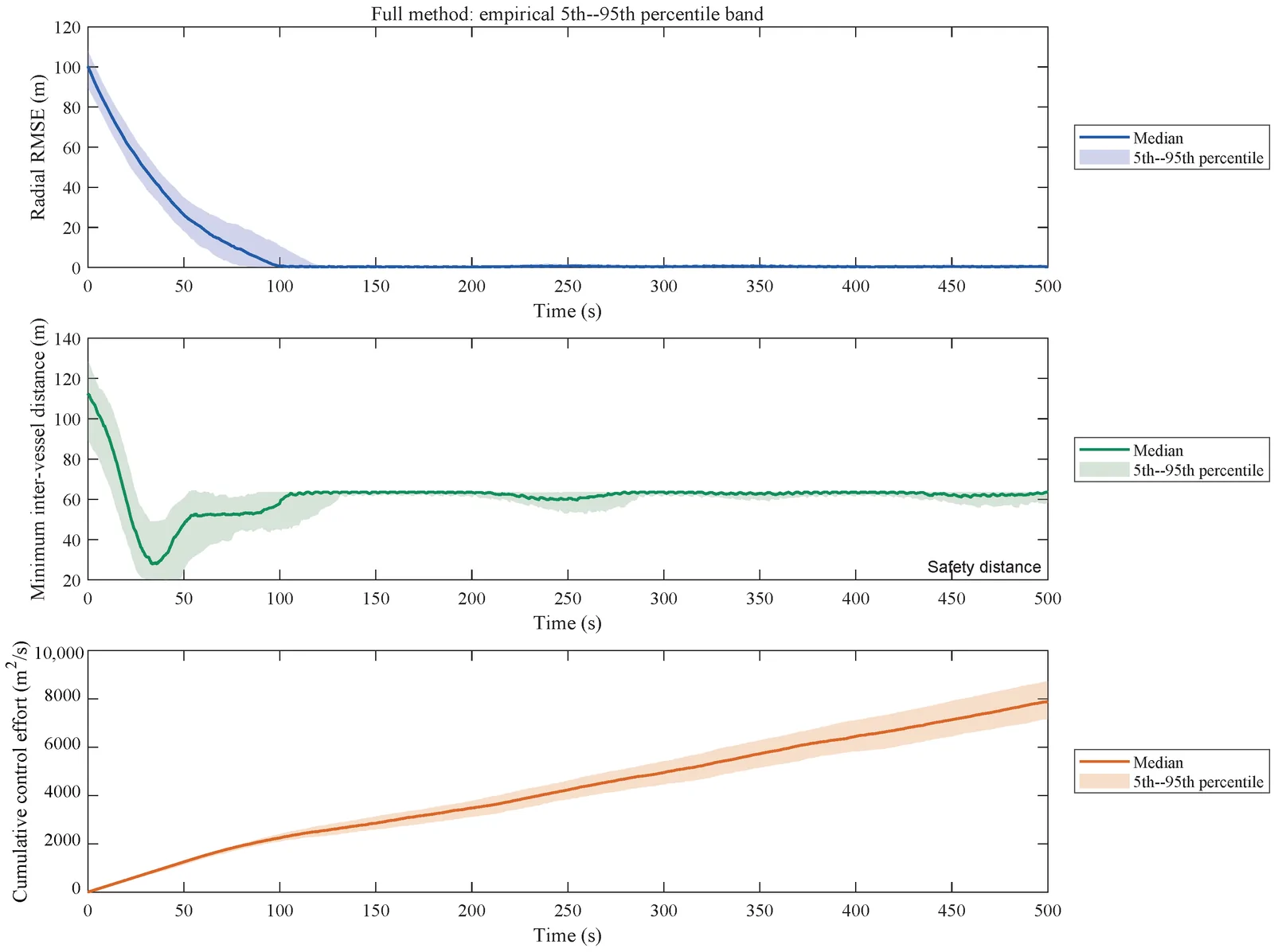
Time histories across 100 Monte Carlo trials: radial Root-Mean-Square Error (RMSE), minimum inter-vessel distance, and cumulative control effort, from top to bottom. Solid curves denote medians, and shaded regions denote empirical 5th–95th percentile bands.

Figure 24 presents the time histories of radial RMSE, minimum inter-vessel distance, and cumulative control effort. The solid curves represent the medians across the 100 trials, and the shaded regions indicate the empirical 5th–95th percentile bands. The radial error decreased rapidly during the initial approach and remained small after encirclement was established. Although the transient responses varied among trials, the radial-error percentile band narrowed substantially as the encirclement configuration formed, indicating consistent tracking performance despite variations in the initial states.

The steady-state radial RMSE was 0.5464±0.1210m, and its maximum across all trials was below 0.912m. The steady-state position RMSE was 2.5363±0.7238m. Position error captures both radial deviations and deviations from the assigned angular positions. Since target maneuvering continuously rotates the reference configuration, angular tracking deviations contribute to a larger position RMSE than radial RMSE. Nevertheless, the 100% encirclement retention confirms that the USVs maintained effective encirclement while continuously adjusting their positions.

**Figure 25:**
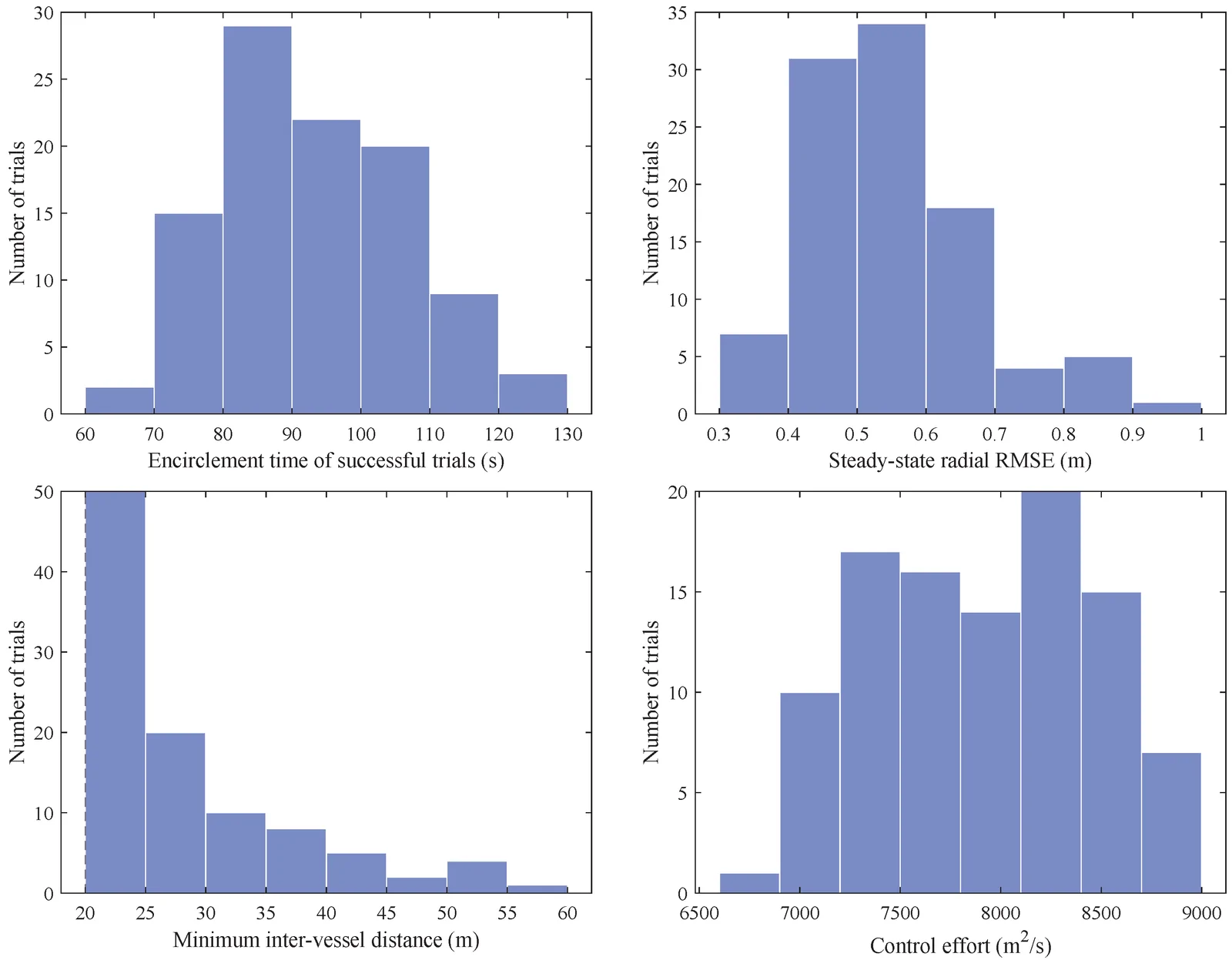
Performance distributions across 100 Monte Carlo trials: encirclement establishment time, steady-state radial RMSE, minimum inter-vessel distance, and control effort. The black dashed vertical line in the lower-left panel indicates the minimum safety distance of 20m.

Figure 25 shows the distributions of the principal performance metrics. The mean encirclement establishment time was 93.42±13.57s, with a median of 93.90s and a 5th–95th percentile interval of [73.89,115.90]s. All trials established encirclement within 127.70s. Under the strict criterion, the mean establishment time was 99.21±13.79s, and the maximum was 131.80s. The 100% success rates under both criteria demonstrate that the trials achieved sustained encirclement with the tighter radial-error tolerance as well as the standard requirement.

Regarding safety, the mean of the trial-wise minimum inter-vessel distances was 27.8812m, and the smallest sampled distance across all trials was 20.0057m. An additional check of relative motion within the integration intervals yielded a minimum distance of 20.00565m, satisfying the prescribed 20m safety threshold. The CBF filter intervened in 42 trials, with an activation ratio averaged across all trials of 0.89% and a maximum trial-wise activation ratio of 5.74%. These results show that the safety filter provided the necessary corrections in relevant configurations while maintaining a low overall intervention ratio.

The minimum inter-vessel distance decreased during the initial configuration adjustment and subsequently increased as encirclement formed. In Figure 24, its median varied approximately between 60 and 64m during the later stage. This is consistent with the adjacent spacing of approximately 63.64m for four equally spaced USVs on a circle of radius 45m. The subsequent fluctuations reflect configuration adjustments induced by target maneuvering, while the minimum distances remained above the safety threshold throughout the trials.

The mean total control effort was $7908.49\pm 526.03\;m^{2}/s$, with a coefficient of variation of approximately 6.65%, indicating relatively consistent overall control requirements across the randomized trials. The mean control efforts over 0–150s and 150–500s were $2867.34\;m^{2}/s$ and $5041.14\;m^{2}/s$, respectively. Dividing these values by the corresponding interval durations gives average control-effort rates of $19.12\;m^{2}/s^{2}$ and $14.40\;m^{2}/s^{2}$, representing a 24.65% reduction in the later stage. After encirclement establishment, the control required for the initial approach and large configuration adjustments decreased. However, continued target acceleration, turning, and reference-configuration rotation still required sustained tracking inputs, explaining the continued growth of cumulative control effort.

**Figure 26:**
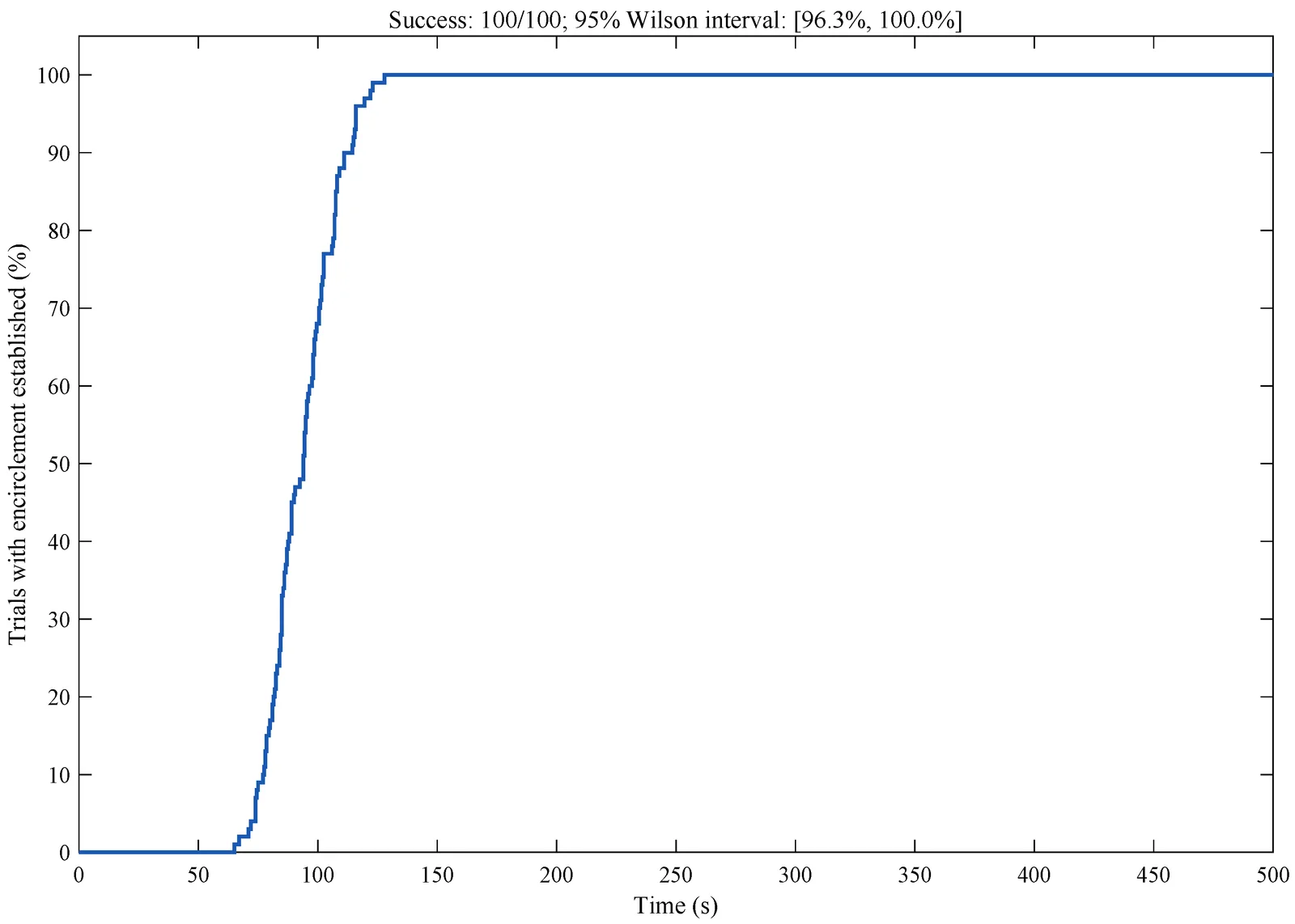
Cumulative proportion of trials with encirclement established across 100 Monte Carlo trials. The success rate is 100%, with a 95% Wilson confidence interval of [96.30%,100%].

Figure 26 further illustrates the cumulative proportion of trials in which encirclement had been established. This proportion reached 68% at 100s, 88% at 110s, and 97% at 120s, reaching 100% at 127.70s. Together with the sustained encirclement retention, these results validate the task-completion reliability, steady-state encirclement accuracy, and safety of the tested tracking-control configuration under the considered randomized conditions.

#### 5.5.2. Verification of the Fixed-Time Convergence Bound

To examine the convergence-time bound of the fixed-time position controller, the initial position errors from the 100 Monte Carlo trials were used to evaluate the ideal error dynamics in Equation (115). An initial-condition-independent upper bound on the settling time is given by

$$T_{b}=\frac{1}{c_{1}\left(1-\alpha \right)}+\frac{1}{c_{2}\left(\beta -1\right)}.$$

Substituting $c_{1}=1.5$, $c_{2}=0.5$, α=0.6, and β=1.4 gives $T_{b}=6.6667\;s$. For each set of initial conditions, the ideal settling time was calculated by numerical quadrature for every position-error component. The maximum component-wise settling time was then taken as the settling time of that error system:

$$T_{\mathrm{ideal}}^{\left(k\right)}=\max_{i,j}\int_{0}^{|e_{i,j}^{\left(k\right)}\left(0\right)|}\frac{d\xi }{c_{1}\xi^{\alpha }+c_{2}\xi^{\beta }},$$

where *k* denotes the trial index, *i* denotes the vessel index, and *j* denotes the Cartesian component of the position error.

**Figure 27:**
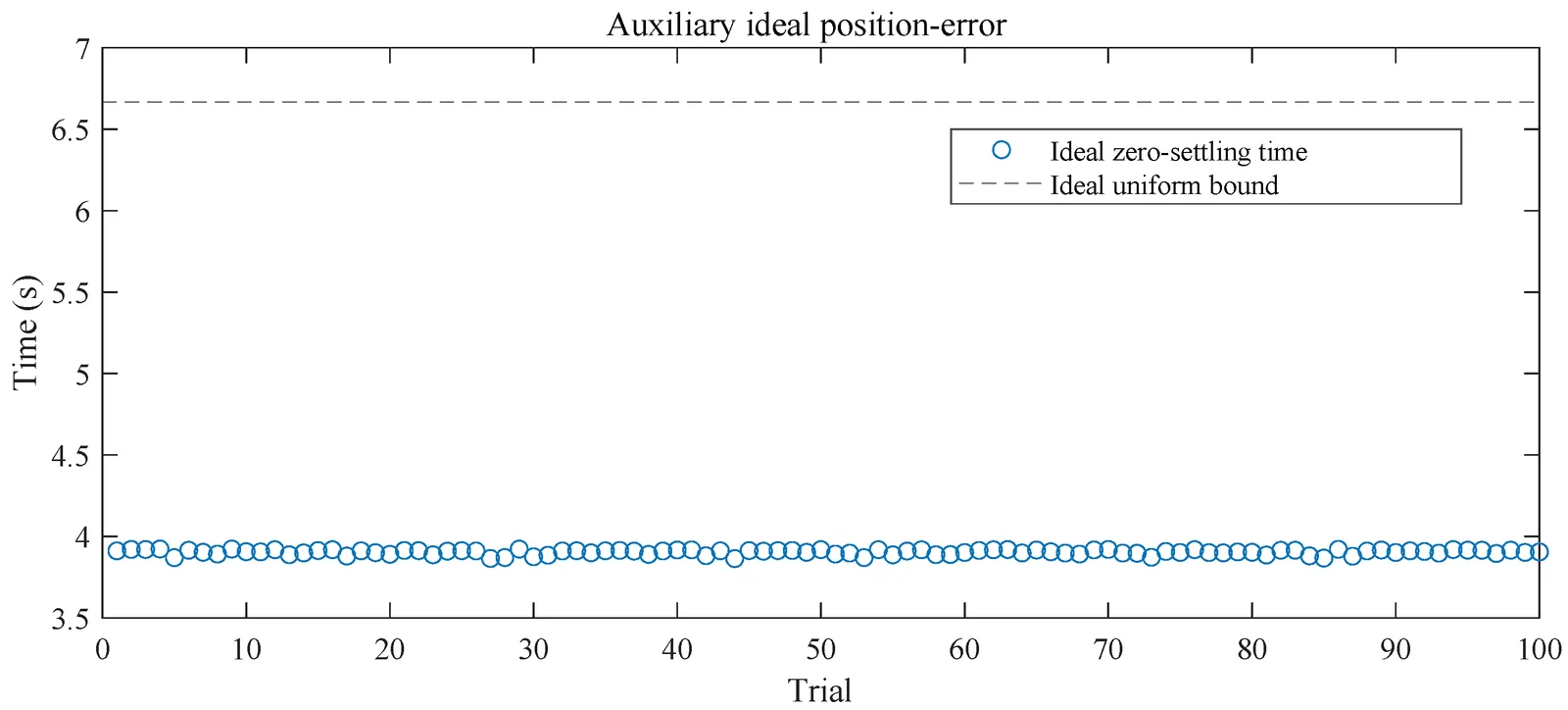
Comparison of ideal position-error settling times under different initial conditions with the uniform theoretical bound. Blue circles denote settling times calculated by numerical quadrature, and the gray dashed line denotes the initial-condition-independent upper bound.

As shown in Figure 27, the ideal settling times for all 100 sets of initial conditions are concentrated around 3.9s, ranging from 3.8641 to 3.9230s. All values remain below the uniform theoretical bound of 6.6667s. The maximum settling time is 2.7437s below the bound and corresponds to approximately 58.84% of it, illustrating the conservatism of the bound. The range of settling times is only 0.0589s, indicating limited variation within the sampled initial conditions. These results provide numerical support for the settling-time bound associated with the ideal position-error subsystem in Equation (115).

## 6. Conclusions

To address the challenges in the dynamic target encirclement process, including difficult cooperative decision-making of USV swarms, strong maneuverability of the target, and the difficulty in satisfying safety constraints, this paper proposes a USV swarm encirclement method integrating biomimetic pursuit-evasion ideas, dynamic game theory and fixed-time safety control. By constructing a pursuit-evasion dynamic game model, adaptive encirclement strategies are generated in response to time-varying target motion states. Combined with fixed-time control and safety constraint mechanisms, the proposed framework improves the fast-tracking performance and collision-free operation of USV swarms. Simulation results reveal that the designed method guides USVs to form stable encirclement formations under ocean current disturbances and continuous target evasive motions. Even with frequent heading and speed adjustments, the target cannot break free from the swarm’s confinement. Moreover, USV position errors converge rapidly and all safety constraints are consistently satisfied, which substantiates the effectiveness and robustness of the proposed algorithm.

Future research directions are summarized as follows: (1) Extend the dynamic game modeling framework to accommodate complex three-dimensional marine environments, obstacle-constrained encirclement, multi-target adversarial scenarios and severe communication limitations, so as to enhance the method’s adaptability to practical marine missions. (2) Integrate reinforcement learning and data-driven optimization to realize online learning and autonomous iteration of game strategies, improving the swarm’s capacity to predict and counteract unknown target motion behaviors. (3) Conduct field ship experiments under real ocean turbulence and practical engineering constraints to further validate the practicability of the proposed control framework.

## Figures and Tables

**Table 1:** Comparison between traditional rule-based encirclement and biomimetic predation-inspired encirclement mechanisms.

Aspect	Traditional Rule-Based Methods	Biomimetic Predation-Inspired Methods
Formation mechanism	Fixed or predefined geometric formation	Dynamic spatial redistribution according to target motion
Response to target maneuver	Reactive adjustment based mainly on tracking errors	Active adjustment of blocking directions according to escape behavior
Cooperative mechanism	Predefined control rules and fixed interaction relationships	Adaptive spatial redistribution based on dynamic game equilibrium
Escape-space suppression	Maintains prescribed relative positions around the target	Progressively compresses the target’s available escape space
Adaptability	Performance may degrade under abrupt target maneuvering or disturbances	Self-organized adjustment according to time-varying pursuit–evasion states
Adversarial capability	Limited explicit representation of active target escape	Naturally captures the coupled interaction between pursuit, blocking, and escape

**Table 2:** Simulation parameters.

Parameter	Value	Description
$c_{1},c_{2}$	1.5,0.6	Fixed-time position control gains
α,β	0.6,1.4	Fixed-time position nonlinear exponents
$c_{3},c_{4}$	1.2,0.5	Fixed-time heading control gains
$\alpha_{\psi },\beta_{\psi }$	0.6,1.4	Fixed-time heading nonlinear exponents
$k_{1},k_{2}$	2.0,1.0	Fixed-time dynamic control gains
m,n	0.7,1.3	Fixed-time dynamic nonlinear exponents
$\lambda_{i}$	10.0	Disturbance observer gain
$k_{s}$	0.8	Safety constraint gain
$k_{\theta }$	0.6	Formation rotation coefficient
$R_{c}$	45m	Desired encirclement radius
$d_{s}$	20m	Minimum safe distance between USVs
$v_{\max}$	2.5m/s	Maximum speed of pursuit USVs
$v_{T}\left(0\right)$	1.5m/s	Initial speed of target vessel
$v_{T}^{\max}$	2.0m/s	Maximum speed of target vessel

**Table 3:** Reward function parameters.

Parameter	Value	Description
$\alpha_{1}$	0.5	Penalty weight for tracking distance error
$\alpha_{2}$	1.0	Penalty weight for formation deviation
$\alpha_{3}$	0.5	Penalty weight for control energy consumption
$\beta_{1}$	0.5	Reward weight for target escape distance
$\beta_{2}$	0.5	Penalty weight for target control energy consumption

**Table 4:** Common simulation settings for the baseline comparison.

Parameter	Value
Number of USVs, *N*	4
Simulation duration, *T*	500s
Sampling interval, Δt	0.1s
Desired encirclement radius, $R_{c}$	45m
Safety distance, $d_{s}$	20m
Command magnitude limit, $u_{\max}$	2.5m/s
Radial tolerance, $\varepsilon_{r}$	5m
Holding duration, $T_{h}$	10s
Strict tolerance and holding duration	2m and 20s
Steady-state evaluation interval	400–500s
Constant current speed, $V_{c}$	0.25m/s
Constant current direction, $\beta_{c}$	π/6
Periodic disturbance amplitude, $A_{w}$	0.12m/s
Angular frequency parameter, $\omega_{w}$	0.08rad/s
Random disturbance standard deviation, $A_{r}$	0.02m/s

**Table 5:** Comparison of encirclement and safety performance. NE indicates that the establishment criterion is not achieved within the simulation horizon; a dash indicates that the metric is not applicable.

Method	$T_{\mathrm{enc}}$ (s)	$T_{\mathrm{enc}}^{\mathrm{strict}}$ (s)	$E_{\mathrm{ss}}$ (m)	$d_{\min}$ (m)	$S_{\mathrm{safe}}$ (%)	$R_{\mathrm{enc}}$ (%)
Proposed method	88.9	92.9	0.533	23.927	100.00	100.00
Consensus formation	110.5	NE	3.949	41.298	100.00	22.18
Leader–follower	NE	NE	6.426	28.250	100.00	–
Artificial potential field	92.3	NE	3.645	48.991	100.00	61.03
Nonlinear encirclement	119.5	455.7	1.485	13.122	83.92	80.32
Predictive encirclement	137.2	144.1	0.358	26.735	100.00	100.00

**Table 6:** Relative changes in encirclement establishment time and steady-state radial error achieved by the proposed method.

Baseline	Establishment Time Reduction (%)	Steady-State Radial Error Reduction (%)
Consensus formation	19.55	86.50
Leader–follower	–	91.70
Artificial potential field	3.68	85.37
Nonlinear encirclement	25.61	64.07
Predictive encirclement	35.20	−49.00

Each reduction is calculated as the baseline value minus the proposed-method value, divided by the baseline value. A negative reduction indicates that the proposed method has a higher value for that metric. A dash indicates that the comparison is not applicable.

**Table 7:** Comparison of control effort and command characteristics.

Method	$J_{u}$ ($m^{2}/s$)	${\bar{J}}_{u}$	$u_{\mathrm{RMS}}$ (m/s)	$P_{\mathrm{sat}}$ (%)
Proposed method	8228.396	0.6583	2.028	47.54
Consensus formation	7269.014	0.5815	1.906	32.45
Leader–follower	7498.220	0.5999	1.936	33.93
Artificial potential field	4951.301	0.3961	1.573	12.35
Nonlinear encirclement	6005.325	0.4804	1.733	3.22
Predictive encirclement	7517.326	0.6014	1.939	37.41

**Table 8:** Control effort over two simulation intervals and the relative increase in total effort of the proposed method over each baseline.

Method	$J_{0-150}$ ($m^{2}/s$)	$J_{150-500}$ ($m^{2}/s$)	Relative Increase (%)
Proposed method	2997.976	5230.420	–
Consensus formation	2857.212	4411.802	13.20
Leader–follower	3026.275	4471.945	9.74
Artificial potential field	2290.437	2660.864	66.19
Nonlinear encirclement	2337.983	3667.341	37.02
Predictive encirclement	2933.659	4583.667	9.46

**Table 9:** Performance of the proposed method under different operating conditions. Establishment time and radial error are reported as mean ± sample standard deviation.

Condition	Establishment Time (s)	Radial Root-Mean-Square Error (m)	Worst-Case Minimum Distance (m)	Mean Normalized Control Effort
Reference condition	71.52±4.39	0.330±0.062	29.6225	0.5064
Six pursuit vessels	96.78±3.76	0.345±0.062	20.0215	0.5115
Eight pursuit vessels	103.88±3.24	0.345±0.062	20.0200	0.5125
Speed ratio of 0.4	56.88±2.26	0.187±0.036	32.8217	0.4277
Speed ratio of 0.8	97.68±7.63	0.523±0.101	30.0315	0.6148
Communication delay of 0.2 s	71.64±4.41	0.461±0.062	29.6960	0.5063
Communication delay of 0.5 s	71.80±4.46	0.674±0.067	29.8246	0.5062
Packet-loss probability of 10%	71.52±4.39	0.338±0.062	29.6228	0.5072
Packet-loss probability of 30%	71.55±4.39	0.363±0.065	29.6814	0.5101

**Table 10:** Comparison of the six methods in the eight-vessel condition.

Method	Encirclement Success (%)	All-Time Safe Trials (%)	Mean Establishment Time (s)	Mean Radial Error (m)	Mean Retention (%)
Proposed method	100	100	103.88	0.345	100.00
Consensus formation	100	0	104.49	2.427	94.47
Leader–follower	15	0	139.23	5.130	5.26
Artificial potential field	100	100	84.63	2.372	93.81
Nonlinear encirclement	100	0	106.20	1.032	96.04
Predictive encirclement	100	30	110.35	0.340	100.00

**Table 11:** Configurations of the ablation study.

Method	Game-Based Decision-Making	Fixed-Time Control	CBF-Based Safety Control	Modification
Full implementation	Retained	Retained	Retained	Full numerical implementation
w/o Game	Removed	Retained	Retained	Uniform circular references without game optimization
w/o Fixed-time	Retained	Replaced	Retained	Linear error feedback replaces fixed-time control
w/o CBF	Retained	Retained	Removed	Saturated nominal commands are applied directly

**Table 12:** Encirclement and safety performance of the ablation configurations. NE indicates that the establishment criterion is not achieved within the simulation horizon; a dash indicates that the metric is not applicable. Safety satisfaction rates are calculated using unrounded distance data.

Method	$T_{\mathrm{enc}}$	$T_{\mathrm{enc}}^{\mathrm{strict}}$	Radial RMSE	Position RMSE	$d_{\min}$	$S_{\mathrm{safe}}$	$R_{\mathrm{enc}}$
	(s)	(s)	(m)	(m)	(m)	(%)	(%)
Full implementation	113.3	131.7	0.205	0.290	20.001	100	100
w/o Game	NE	NE	594.690	639.920	20.305	100	–
w/o Fixed-time	126.5	148.6	0.273	0.386	20.000	100	100
w/o CBF	88.9	114.4	0.171	0.242	19.203	97.141	100

**Table 13:** Control effort of the ablation configurations.

Method	$J_{u}$ ($m^{2}/s$)	${\bar{J}}_{u}$	Saturation Rate (%)
Full implementation	3946.5	0.31572	24.825
w/o Game	1872.6	0.14981	98.590
w/o Fixed-time	4070.1	0.32560	27.480
w/o CBF	3649.9	0.29199	19.775

**Table 14:** Statistical results of 100 Monte Carlo trials for tracking encirclement.

Metric	Mean ± SD	Minimum	Maximum
Encirclement establishment time (s)	93.42±13.57	64.80	127.70
Encirclement confirmation time (s)	103.42±13.57	74.80	137.70
Strict encirclement establishment time (s)	99.21±13.79	69.20	131.80
Steady-state radial RMSE (m)	0.5464±0.1210	0.3150	0.9116
Steady-state position RMSE (m)	2.5363±0.7238	1.3549	4.8850
Minimum inter-vessel distance (m)	27.8812±9.2104	20.0057	55.2772
Safety satisfaction (%)	100±0	100	100
Encirclement retention (%)	100±0	100	100
Control effort ($m^{2}/s$)	7908.49±526.03	6810.10	8887.42
Normalized control effort	0.6327±0.0421	0.5448	0.7110
Control-command RMS (m/s)	1.9874±0.0662	1.8453	2.1080
Saturation ratio (%)	43.51±4.37	34.56	52.69

## Data Availability

The original contributions presented in this study are included in the article. Further inquiries can be directed to the corresponding author.
